# Mechanism and Regulation of DNA-Protein Crosslink Repair by the DNA-Dependent Metalloprotease SPRTN

**DOI:** 10.1016/j.molcel.2016.09.031

**Published:** 2016-11-17

**Authors:** Julian Stingele, Roberto Bellelli, Ferdinand Alte, Graeme Hewitt, Grzegorz Sarek, Sarah L. Maslen, Susan E. Tsutakawa, Annabel Borg, Svend Kjær, John A. Tainer, J. Mark Skehel, Michael Groll, Simon J. Boulton

**Affiliations:** 1 The Francis Crick Institute, 1 Midland Road, London NW1 1AT, UK; 2 Center for Integrated Protein Science at the Department Chemie, Lehrstuhl für Biochemie, Technische Universität München, Lichtenbergstrasse 4, 85747 Garching, Germany; 3 MRC Laboratory of Molecular Biology, Francis Crick Avenue, Cambridge CB2 0QH, UK; 4 Molecular Biophysics and Integrated Bioimaging, Lawrence Berkeley National Laboratory, Berkeley, CA 94720, USA; 5 Department of Molecular and Cellular Oncology, The University of Texas M.D. Anderson Cancer Center, Houston, TX 77030, USA

**Keywords:** DNA-protein crosslinks, DNA repair, DVC1, formaldehyde, hepatocellular carcinoma, progeria, protease, Ruijs-Aalfs syndrome, SPRTN, Spartan, topoisomerase, Wss1

## Abstract

Covalent DNA-protein crosslinks (DPCs) are toxic DNA lesions that interfere with essential chromatin transactions, such as replication and transcription. Little was known about DPC-specific repair mechanisms until the recent identification of a DPC-processing protease in yeast. The existence of a DPC protease in higher eukaryotes is inferred from data in *Xenopus laevis* egg extracts, but its identity remains elusive. Here we identify the metalloprotease SPRTN as the DPC protease acting in metazoans. Loss of SPRTN results in failure to repair DPCs and hypersensitivity to DPC-inducing agents. SPRTN accomplishes DPC processing through a unique DNA-induced protease activity, which is controlled by several sophisticated regulatory mechanisms. Cellular, biochemical, and structural studies define a DNA switch triggering its protease activity, a ubiquitin switch controlling SPRTN chromatin accessibility, and regulatory autocatalytic cleavage. Our data also provide a molecular explanation on how SPRTN deficiency causes the premature aging and cancer predisposition disorder Ruijs-Aalfs syndrome.

## Highlights

• The SPRTN metalloprotease repairs DNA-protein crosslinks
• A DNA switch controls SPRTN’s protease activity
• A ubiquitin switch controls chromatin access of SPRTN
• Structural insights reveal unique features of the SPRTN/Wss1 protease family

## Graphical Abstract

**Figure:**
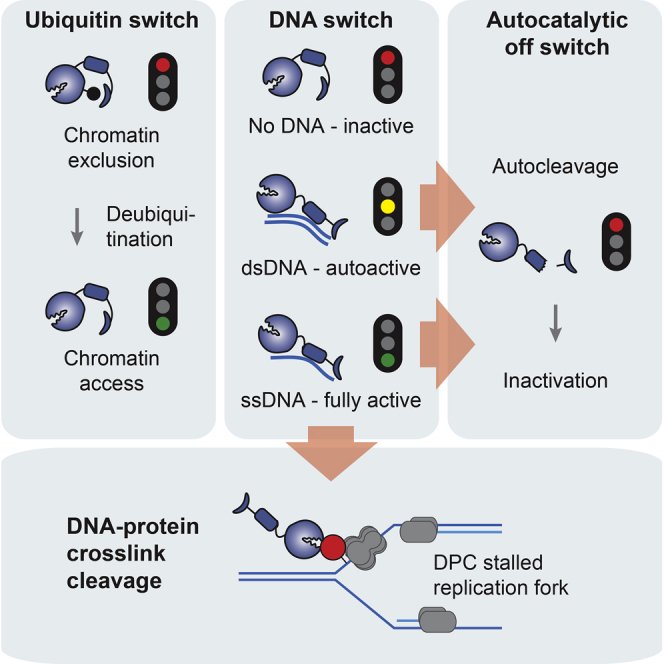

## Teaser

Stingele et al. discover the SPRTN metalloprotease to be crucial for DNA-protein crosslink repair in higher eukaryotes. In addition, several regulatory principles constraining SPRTN’s potentially toxic activity are described: a ubiquitin switch controlling chromatin access, a DNA switch triggering protease activity, and a negative feedback loop based on autocatalytic cleavage.

## Introduction

The integrity of DNA is constantly challenged by structural and chemical alterations (Lindahl, 1993). Hence, restoration of the native DNA sequence and structure by damage-specific repair mechanisms is essential to ensuring genome stability. Germline mutations in crucial DNA repair enzymes result in several premature aging and cancer predisposition syndromes, highlighting the fundamental importance of DNA repair in mammals (Jackson and Bartek, 2009). DNA lesions range from abasic sites, small and bulky adducts, to single- and double-strand breaks, which are repaired by lesion-specific and generally well-understood mechanisms (Friedberg et al., 2014). However, specific repair mechanisms for one particular type of lesion, covalent DNA-protein crosslinks (DPCs), have remained elusive. This is despite DPCs being extremely toxic as they directly block essential chromatin transactions, such as replication and transcription (Fu et al., 2011, Nakano et al., 2012, Nakano et al., 2013).

Covalent crosslinking of proteins to DNA can be caused by various exogenous agents, such as ionizing radiation (IR), UV light, certain metal ions, and, importantly, platinum-based chemotherapeutics such as cisplatin and derivatives (Barker et al., 2005, Stingele and Jentsch, 2015). Moreover, DPCs are induced by endogenously produced reactive metabolites, such as formaldehyde or acetaldehyde. Notably, formaldehyde is directly produced within chromatin as a by-product of the histone demethylation reaction (Shi et al., 2004, Swenberg et al., 2011). Furthermore, abasic sites bear an aldehyde group that efficiently reacts with nucleosome proteins, thereby forming DPCs (Sczepanski et al., 2010). DPCs also are produced enzymatically by the entrapment of normally transient covalent DNA-protein reaction intermediates of enzymes, such as topoisomerases 1 and 2 (TOP1 and TOP2). Distortions within the DNA (e.g., caused by nearby DNA damage) or small molecules such as camptothecin (CPT) or etoposide inhibit religation and result in stable DPC formation (Pommier, 2006). These enzymatic DPCs can be reversed by specific tyrosil-DNA phosphodiesterases (TDP1 and TDP2 acting on TOP1- and TOP2- adducts, respectively), which hydrolyze the covalent bond between the topoisomerase’s active site residue and the DNA (Pommier et al., 2014). Apart from these unique cases of enzymatic DPCs, it has been suggested that DPCs are generally repaired by canonical DNA repair pathways, such as nucleotide excision repair and homologous recombination (Baker et al., 2007, de Graaf et al., 2009, Nakano et al., 2007, Nakano et al., 2009).

Very recently a protease-based DPC repair mechanism was discovered in budding yeast (Stingele et al., 2014) and in *Xenopus laevis* egg extracts (Duxin et al., 2014). In yeast, DPC proteolysis is catalyzed by the metalloprotease Wss1, which permits replication in the presence of DPCs and provides resistance toward DPC-inducing agents. Intriguingly, Wss1 is a DNA-dependent protease that degrades DNA-bound substrates in vitro irrespective of identity. Importantly, in *Xenopus* egg extracts, a DPC-containing plasmid is repaired by a similar mechanism, indicating that protease-based DPC repair is conserved. However, the identity of the DPC protease operating in higher eukaryotes has remained elusive.

Spartan (SPRTN, DVC1) is distantly related to yeast Wss1, displays a similar domain organization, and shares a common evolutionary origin (Stingele et al., 2015). Germline mutations of *SPRTN* are causative for Ruijs-Aalfs syndrome (RJALS), which is characterized by genome instability, premature aging, and early-onset hepatocellular carcinoma (Lessel et al., 2014). Mice deficient for SPRTN are embryonically lethal, and hypomorphic mutant animals display hallmarks of premature aging and genome instability (Maskey et al., 2014). While SPRTN is clearly essential for genome stability, its molecular function remains unclear. Initial studies suggested that SPRTN is important for regulating translesion synthesis (TLS), although with conflicting reports on the actual molecular mechanism (Centore et al., 2012, Davis et al., 2012, Ghosal et al., 2012, Juhasz et al., 2012, Machida et al., 2012, Mosbech et al., 2012). Importantly, however, the severe phenotypes observed in flies, mice, and human cells have been shown to be unrelated to TLS, suggesting that SPRTN maintains genome integrity by an unknown mechanism distinct from TLS (Delabaere et al., 2014, Lessel et al., 2014, Maskey et al., 2014).

Here we identify SPRTN as the elusive DPC protease in higher eukaryotes. Using cellular, biochemical, and structural data, we establish the mechanism of SPRTN’s DNA-dependent proteolytic activity, and we identify several safeguarding mechanisms that act to constrain SPRTN’s potentially toxic activity, including a ubiquitin switch regulating its chromatin accessibility and a negative feedback loop based on autocatalytic cleavage.

## Results

### SPRTN-Deficient Worms Are Hypersensitive to DPC-Inducing Agents

**Figure 1:**
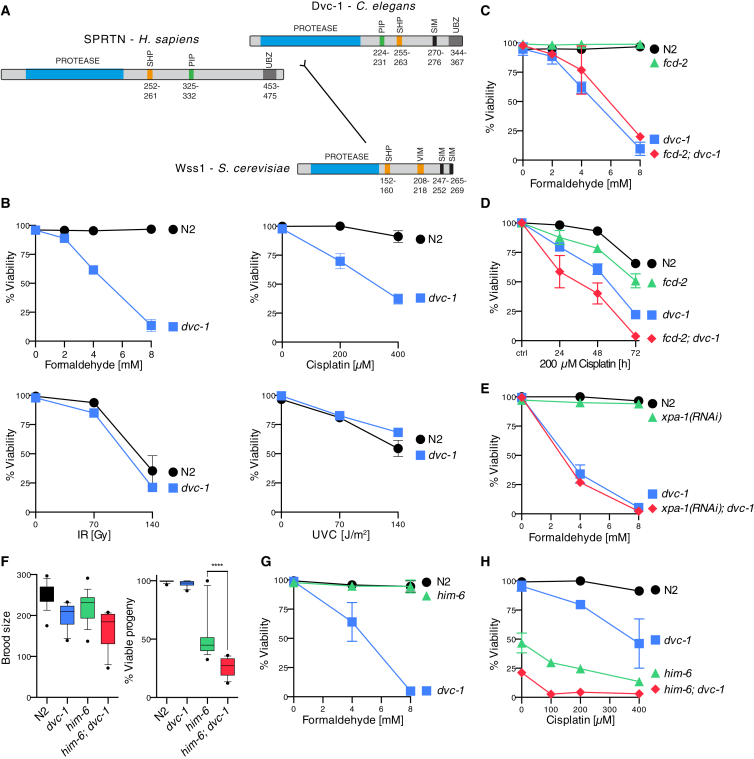
SPRTN/Dvc-1 Provides Resistance toward DPC-Inducing Agents in Worms and Operates Independently of FANCD2/Fcd-2 and Parallel to BLM/Him-6 (A) Domain structures and evolutionary distances of the protease domain of SPRTN/Wss1 protease family members of humans, worms, and budding yeast. SPRTN/Wss1 proteases bear interaction domains for p97/Cdc48 (SHP-box, VIM), recognition modules for ubiquitin (UBZ) or SUMO (SIM), and in metazoans a PCNA-interaction motif (PIP-box). (B) *C. elegans* mutant strains lacking functional SPRTN (*dvc*-*1*) are specifically sensitive to the DPC-inducing agents. Formaldehyde sensitivity was determined in synchronized L1 larvae. Cisplatin, UVC light, and IR sensitivities were assessed by measuring embryonic survival of progeny after exposure of adult animals. Error bars indicate SEM of –two to four independent experiments. (C) FANCD2 is not involved in providing formaldehyde resistance in synchronized L1 larvae. Error bars indicate SEM of two independent experiments. (D) FANCD2 provides resistance to chronic cisplatin exposure by a mechanism distinct to DPC repair by SPRTN. Viability was assessed by determination of embryonic survival of progeny of young adult animals kept on cisplatin-containing plates (200 μM) for the indicated amount of time. Error bars indicate SEM of two independent experiments. (E) Loss of XPA does not result in increased formaldehyde sensitivity in synchronized L1 worms. Error bars indicate SEM of two independent experiments. (F) Loss of SPRTN (*dvc*-*1*) results in viability defects in worms lacking the BLM helicase (*him*-*6*). Data were obtained from at least 16 animals per indicated genotype. Whiskers indicate tenth to 90th percentiles. Statistical significance was tested using an unpaired t test. (G) The BLM helicase (Him-6) is not involved in providing formaldehyde resistance in synchronized L1 larvae. Error bars indicate SEM of two independent experiments. (H) BLM (Him-6) provides resistance to cisplatin exposure by a mechanism parallel to DPC repair by SPRTN. Cisplatin sensitivity was assessed by measuring embryonic survival of progeny after exposure of adult animals. Error bars indicate SEM of two independent experiments. See also Figure S1.

The SPRTN metalloprotease is essential for viability in mammalian cells, which complicates the analysis of its precise molecular function. Despite being closely related to the mammalian enzyme (Figure 1A; evolutionary distances from Stingele et al., 2015), the nematode ortholog of SPRTN (called Dvc-1) is dispensable for viability (Mosbech et al., 2012). Thus, we set out to investigate a potential role for SPRTN in DPC repair and its interaction with canonical DNA repair pathways in worms by assessing sensitivity to DPC-inducing agents. DNA damage sensitivity is typically measured in worms by treating young adult animals, followed by determining viability of their progeny as a proxy for repair defects in the germline, which is the only proliferating tissue in adult animals (Figure S1A). However, this treatment regimen is not suitable for testing formaldehyde sensitivity, because treated adult worms succumb to death at doses that have no effect on progeny viability; this is likely due to formaldehyde being unable to penetrate into the germline, similar to what has been observed with mitomycin C. Thus, we employed an alternative protocol that determines sensitivity by exposing young L1 larvae arrested by starvation (Figure S1A).

Strikingly, SPRTN-deficient L1 larvae (*dvc*-*1*) were extremely sensitive to an acute exposure to formaldehyde when compared to wild-type (WT) (N2) controls (Figure 1B). Furthermore, SPRTN-deficient worms (*dvc*-*1*) were very sensitive to cisplatin (induces both DPCs and inter-strand crosslinks [ICLs]) using the classical treatment regimen. In contrast, SPRTN-deficient worms were resistant to DNA damage induced by UV light or IR, consistent with a specific function in DPC repair (Figure 1B). Notably, SPRTN-deficient worms tolerated Top1 adducts induced by CPT (Figure S1B).

The Fanconi anemia (FA) pathway provides resistance toward crosslinking compounds by coordinating replication-coupled repair of ICLs (Kottemann and Smogorzewska, 2013). Moreover, cells lacking the FA pathway are hypersensitive to reactive aldehydes, such as formaldehyde or acetaldehyde (Langevin et al., 2011, Rosado et al., 2011). However, a deficiency in the FA protein FANCD2 (*fcd*-*2*) in worms did not result in increased formaldehyde sensitivity of synchronized L1 larvae, even if SPRTN (*dvc*-*1*) also was deleted (Figure 1C). Given that the FA pathway is a replication-coupled repair pathway, this result was perhaps not surprising, because cells of synchronized L1 larvae are arrested at the G1/S transition and are devoid of detectable DNA replication (Baugh, 2013). In agreement with previous studies, FANCD2-deficient worms were sensitive to cisplatin when exposed chronically (Figures 1D and S1C). Notably, worms lacking SPRTN were significantly more sensitive to cisplatin than *fcd*-*2* mutant animals, with the double mutant showing an additive, but not synergistic, phenotype. This indicates that FANCD2 and SPRTN operate in genetically distinct DNA repair pathways, which cannot compensate for each other. In turn, this suggests that they repair two different types of damage caused by cisplatin, which are presumably ICLs and DPCs, respectively.

Nucleotide excision repair (NER) has been implicated in replication-independent repair of formaldehyde-induced DPCs in several organisms. However, we did not observe increased formaldehyde sensitivity in worms lacking the crucial NER factor XPA (*xpa*-*1*), even in the absence of SPRTN (Figures 1E, S1D, and S1E). In yeast, homologous recombination acts parallel to DPC proteolysis by Wss1, and loss of the HR factor BLM (*him*-*6* in worms) is synthetically lethal when combined with a Wss1 knockout (Mullen et al., 2011). Consistently, worms lacking both SPRTN and BLM (*dvc*-*1*; *him*-*6*) showed severe viability defects (Figure 1F). Worms lacking HIM-6/BLM were hypersensitive to cisplatin treatment, but not to acute formaldehyde exposure, in arrested L1 larvae, which implies that BLM acted exclusively in dividing cells (Figures 1G and 1H). The *dvc*-*1*; *him*-*6* double-mutant worms exhibited a synthetic effect, as they were unable to tolerate even very low doses of cisplatin. Taken together, our data reveal that SPRTN provides resistance toward DPC-inducing agents in worms by a mechanism acting parallel to BLM and independent of the FA pathway.

### SPRTN-Deficient Mammalian Cells Fail to Repair Formaldehyde-Induced DPCs

**Figure 2:**
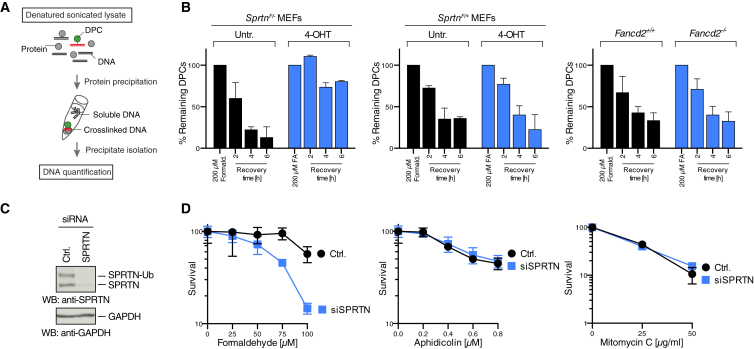
SPRTN-Deficient Mammalian Cells Fail to Repair DPCs and Are Hypersensitive toward DPC-Inducing Agents (A) Schematic representation of the KCl/SDS precipitation assay used to measure DPC repair. Cells are lysed in denaturing conditions (1% SDS), followed by sonication and precipitation of cellular protein by the addition of KCl. Crosslinked DNA co-precipitates with the protein, whereas free DNA remains in the supernatant. The precipitate is washed several times before quantification of soluble and crosslinked DNA. (B) SPRTN-deficient MEFs fail to repair formaldehyde-induced DPCs. *Sprtn*^*F*/−^, *Sprtn*^*F*/+^ (untreated or treated with 4-hydroxy tamoxifen [4-OHT] for 48 hr), *Fancd2*^+/+^, and *Fancd2*^−/−^ MEFs were treated with 200 μM formaldehyde (FA) for 1 hr to induce DPCs and lysed directly or allowed to repair. DPCs were measured as the ratio of crosslinked DNA compared to total DNA. Error bars indicate SEM of two independent experiments. (C and D) Knockdown of SPRTN results in formaldehyde sensitivity in human cells. Relative cell numbers were determined 6 days after U2OS cells transfected with SPRTN or control siRNA were treated with the indicated doses of formaldehyde, aphidicolin, or mytomicin C. Error bars represent SD of two to four replicates. See also Figure S2.

The particular sensitivities of SPRTN-deficient worms suggest a specific function for SPRTN in the repair of DPCs. Hence, we tested this possibility directly by measuring DPC repair capacity in mammalian cells. To this end, we induced DPCs in immortalized conditional *Sprtn*^*F*/*−*^ mouse embryonic fibroblasts (MEFs) by formaldehyde and followed DPC repair using a KCl/SDS precipitation assay (Figure 2A) (Zhitkovich and Costa, 1992). MEFs expressing functional SPRTN efficiently repaired formaldehyde-induced DPCs over time. Strikingly, however, inactivation of the remaining SPRTN allele in *Sprtn*^*F*/−^ MEFs (but not in *Sprtn*^*F*/+^ cells) resulted in an almost complete failure to repair DPCs (Figure 2B). In contrast, no DPC repair defect could be observed in MEFs lacking *Fancd2*.

Next we asked whether the failure to repair DPCs translates to hypersensitivity toward DPC-inducing agents in mammalian cells. Because a complete knockout results in lethality, we utilized knockdown of SPRTN by small interfering RNA (siRNA) in U2OS cells, which indeed resulted in sensitivity toward formaldehyde, but not toward ICL induction by mitomycin C or general replication inhibition by aphidicolin (Figures 2C and 2D).

### A DNA Switch Controls SPRTN’s Protease Activity

**Figure 3:**
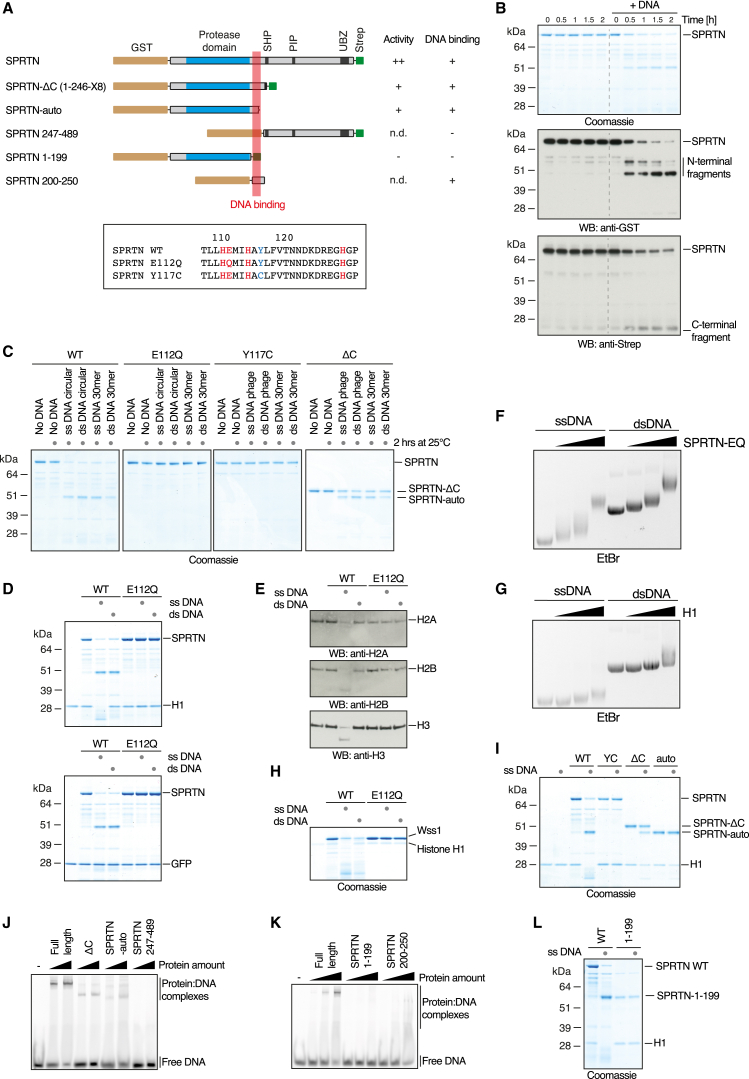
A DNA Switch Controls SPRTN’s Protease Activity (A) Schematic representation of recombinant GST-SPRTN-Strep variants (upper panel). Sequence of SPRTN’s active site with catalytic residues in red and tyrosine-to-cysteine replacement found in RJALS patients in blue (lower panel) are shown. (B) Autocatalytic cleavage of SPRTN is induced by DNA. SPRTN (180 nM, N-terminally GST tagged, C-terminally Strep tagged) was incubated in the absence or presence of circular ssDNA (ΦX174 virion, 10 nM). (C) Autocatalytic cleavage of SPRTN is induced by various types of DNA. GST-SPRTN-Strep (WT, E112Q, or the disease variants Y117C and ΔC, 180 nM) was incubated in the presence of different types of DNA (phage DNA [10 nM], 30-mer oligonucleotides [1.8 μM]) for 2 hr at 25°C. (D and E) SPRTN cleaves DNA-binding proteins in an ssDNA-dependent manner. GST-SPRTN-Strep (WT or the catalytically inactive E112Q variant, 480 nM) was incubated with the indicated substrates (360 nM) in the absence or presence of ss and ds phage DNA (10 nM) for 2 hr at 25°C. (F and G) SPRTN and histone H1 bind similarly to ss and ds phage DNA. Proteins (SPRTN [0.45, 0.9, and 1.8 μM] and H1 [2, 3, and 4 μM]) were incubated with DNA (50 nM) and analyzed on 0.8% agarose gels. (H) Wss1 cleaves histone H1 in an ssDNA-dependent manner. Wss1 (WT or the catalytically inactive E116Q variant, 800 nM) histone H1 (200 nM) were incubated with the indicated type of DNA (10 nM) for 2 hr at 30°C. (I) SPRTN disease variants display defects in ssDNA-dependent substrate cleavage. GST-SPRTN-Strep (WT, Y117C, ΔC or auto, 480 nM) was incubated with histone H1 (360 nM) in the absence or presence of ss phage DNA (10 nM) for 2 hr at 25°C. (J) C-terminally truncated SPRTN variants retain the ability to bind DNA. Indicated proteins (500 nM and 1 μM) were incubated with a fluorescently labeled ss oligonucleotide (250 nM) prior to gel electrophoresis in 6% PAGE gels. (K) SPRTN’s DNA-binding domain resides within aa 200–250. Indicated proteins (0.25, 0.5, and 1 μM) were incubated with a fluorescently labeled ss oligonucleotide (250 nM) prior to gel electrophoresis in 6% PAGE gels. (L) SPRTN deficient for DNA binding is deficient for ssDNA-dependent substrate cleavage. Recombinant GST-SPRTN-Strep (WT or 1–199, 480 nM) was incubated with recombinant histone H1 (360 nM) in the absence or presence of ss phage DNA (10 nM) for 2 hr at 25°C See also Figure S2.

Our finding that SPRTN provides resistance to DPCs and is required for DPC repair suggests that it is indeed the elusive protease required for DPC processing in metazoans. To formally test this possibility in vitro, we purified human SPRTN (N-terminally GST tagged, C-terminally Strep tagged) (Figure 3A) from insect cells, and we assessed it for proteolytic activity toward DNA-associated proteins. In isolation, SPRTN exhibited no detectable proteolytic activity. However, the addition of DNA induced endoproteolytic autocleavage (Figure 3B), which also was observed with the yeast DPC-processing enzyme Wss1 (Stingele et al., 2014). Autoprocessing of SPRTN was induced with different types of single-stranded DNA (ssDNA) and double-stranded DNA (dsDNA), but it was not observed if the active site glutamate residue was mutated to glutamine (E112Q, SPRTN-EQ) (Figures 3C and S2A). In agreement with SPRTN being a metalloprotease, the chelating compound 1,10-phenanthroline (OPA) inhibited autocleavage (Figure S2B).

We next assessed if the DNA-induced proteolytic activity of SPRTN is capable of cleaving DNA-associated proteins irrespective of identity. Indeed, SPRTN efficiently digested the DNA-binding proteins histone H1, H2A, H2B, H3, and Hmg1 in the presence of DNA, but it had no measurable activity toward non-DNA-binding proteins, such as GFP or BSA (Figures 3D, 3E, and S2C–S2G). Strikingly, and in contrast to autocleavage, substrates were digested only in the presence of ssDNA, but not dsDNA. Importantly, both SPRTN and its substrate (histone H1) bound very similarly to the ss and ds phage DNA used for activation (Figures 3F and 3G). Thus, our results indicate that SPRTN’s protease activity is controlled by a DNA-specific switch, which allows the enzyme to operate in two modes: autocleavage only (dsDNA) or substrate and autocleavage (ssDNA). Intriguingly, cleavage of histone H1 by yeast Wss1 displayed the same DNA specificity, suggesting that the DNA switch is a universal feature of this protease family (Figure 3H).

The premature aging and cancer predisposition observed in RJALS patients are caused by mutations of the *SPRTN* gene, resulting in a C-terminally truncated protein (ΔC, amino acid [aa] 1–246 of SPRTN followed by eight amino acids [X8] caused by a frameshift) or a tyrosine-to-cysteine substitution (Y117C, SPRTN-YC) in close proximity to the active site (Figure 3A) (Lessel et al., 2014). Two affected patients were compound heterozygous for SPRTN-ΔC and SPRTN-Y117C, whereas the other reported patient possessed two alleles of the SPRTN-ΔC. To determine how these alterations affect the activity of SPRTN, we tested recombinantly expressed disease variants for DNA-dependent protease activity. Remarkably, SPRTN-Y117C was defective for DNA-dependent autocleavage as well as ssDNA-dependent substrate digestion (Figures 3C and 3I). In contrast, SPRTN-ΔC retained autocleavage activity, producing the same distinct N-terminal fragment as the WT enzyme, which we designate here as SPRTN-auto (Figures 3A and 3C). SPRTN-ΔC also digested substrates in an ssDNA-dependent manner; however, it showed reduced activity compared to WT enzyme (Figures 3I and S2H). To understand if the processing of SPRTN-ΔC into SPRTN-auto changes its activity, we purified the processed fragment (Figure S2I). However, SPRTN-auto was indistinguishable from SPRTN-ΔC with respect to substrate cleavage (Figure 3I).

The DNA-dependent activity of SPRTN-ΔC and SPRTN-auto suggests that these variants retain the ability to bind DNA. Indeed, both proteins shifted ssDNA in an electrophoretic mobility shift assay (EMSA), only to a slightly lesser extent than the full-length protein (Figure 3J). Conversely, the C-terminal part of SPRTN (SPRTN 247–489) did not show any DNA binding (Figure 3J). Next, we mapped the DNA-binding domain of SPRTN further to the region directly C-terminal to the protease domain. The aa 200–250 of SPRTN expressed as a GST-fusion shift DNA, while a SPRTN variant lacking this region (SPRTN 1–199) did not show DNA-binding activity (Figure 3K). Importantly, SPRTN lacking the DNA-binding domain (SPRTN 1–199) was devoid of detectable DNA-dependent protease activity, indicating that DNA binding is required for its activity (Figure 3L).

### Crystal Structure of the Protease Domain of SPRTN’s Fission Yeast Homolog

Our results suggest that the DNA-dependent activity of the SPRTN/Wss1 protease family is critical for its function in vivo. This activity is highly specific and promiscuous at the same time; only DNA-binding proteins are digested in a strictly DNA-dependent manner, yet irrespective of identity. To gain insights into how this feat is achieved, we sought to obtain structural information on this protease family. We focused on the protease domain, as the presumably highly dynamic C-terminal tail containing various protein-protein interaction motifs interferes with crystallization of full-length protein. We screened several constructs of different SPRTN/Wss1 representatives from various organisms for the expression in *E. coli.* While most proteins were insoluble, we were able to purify the protease domain of Wss1b, one of the two SPRTN homologs in *Schizosaccharomyces pombe*. Wss1b was crystallized and its structure determined by single-wavelength anomalous dispersion (SAD) at a resolution of 1.0 Å and R_free_ = 16.8% (PDB: 5JIG) (Table 1). An X-ray fluorescence spectrum of crystals obtained at the synchrotron revealed the presence of nickel as the only heavy-metal atom. An anomalous dataset at the Ni^2+^ edge (z = 1.4854) confirmed that this ion occupies the active site of the enzyme, where it replaced the catalytic zinc presumably during the Ni^2+^-affinity chromatography.

**Figure 4:**
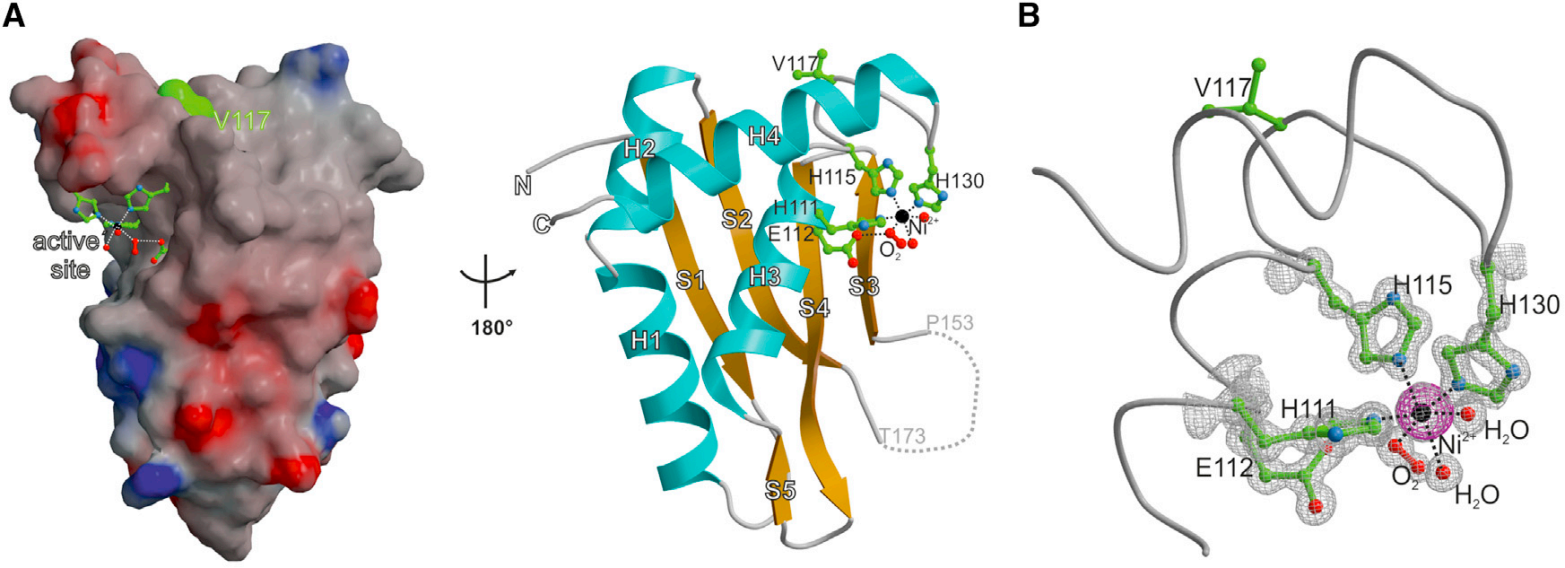
Crystal Structure of the Protease Domain of SPRTN’s Fission Yeast Homolog (A) Structure of the protease domain of *S. pombe* Wss1b (PDB: 5JIG) in a surface (left) or cartoon (right) representation. Active site residues are displayed as sticks. Position 117 mutated in RJALS to a cysteine is highlighted in green. Numbering of residues corresponds to the human sequence. (B) Close-up view of the active site showing the octahedral coordination of Ni^2+^ by His111, His115, His130, as well as one oxygen and two water molecules. The 2F_O_-F_C_ electron density map is contoured to 1σ, whereas the anomalous density (magenta) for Ni^2+^ is contoured to 10σ. Most likely the catalytic zinc atom has been replaced during the Ni^2+^-affinity chromatography step. See also Figure S3.

The overall architecture depicted a compact protease domain consisting of four tightly packed α helices and a four-stranded antiparallel β sheet (Figure 4A). The catalytic center was formed by three histidines, as well as two water molecules and one oxygen molecule, that jointly coordinated the active site metal ion by forming a distorted octahedron (Figure 4B). The glutamate residue E112 polarized a water molecule for the nucleophilic attack of the substrate; in agreement, its mutation to glutamine resulted in a catalytically inactive enzyme (Figure 3). Moreover, we solved the structure of the EQ mutant (PDB: 5LN5) (Figure S3A), which confirmed that this mutation does not result in general structural alterations, thereby validating our biochemical analysis. Intriguingly, the catalytic center comprising the metal-binding motif was highly solvent exposed. This together with the absence of an obvious substrate-binding cleft or region could explain the promiscuity of SPRTN/Wss1 proteases with respect to substrate identity. A structure-based search for homologous topologies using the DALI-server revealed structures with *Z* scores < 9. All hits displayed a sequence identity of <10% and differed in at least one active site residue.

Intriguingly, the structure revealed that position 117 mutated in RJALS is in close proximity to the active site residues, but it does not seem to be involved in metal binding (Figure 4B). This position is only conserved in higher eukaryotes, suggesting that it acquired an important function only later in evolution (Figure S3B). Interestingly, this residue is solvent exposed and is followed in metazoans by a conserved insertion. This lobe is positioned next to the active site and might be required for stable substrate binding. The change to a cysteine residue at position 117 could result in a tilting of this lobe, thereby interfering with substrate binding.

### DNA Binding Induces Conformational Changes in SPRTN

**Figure 5:**
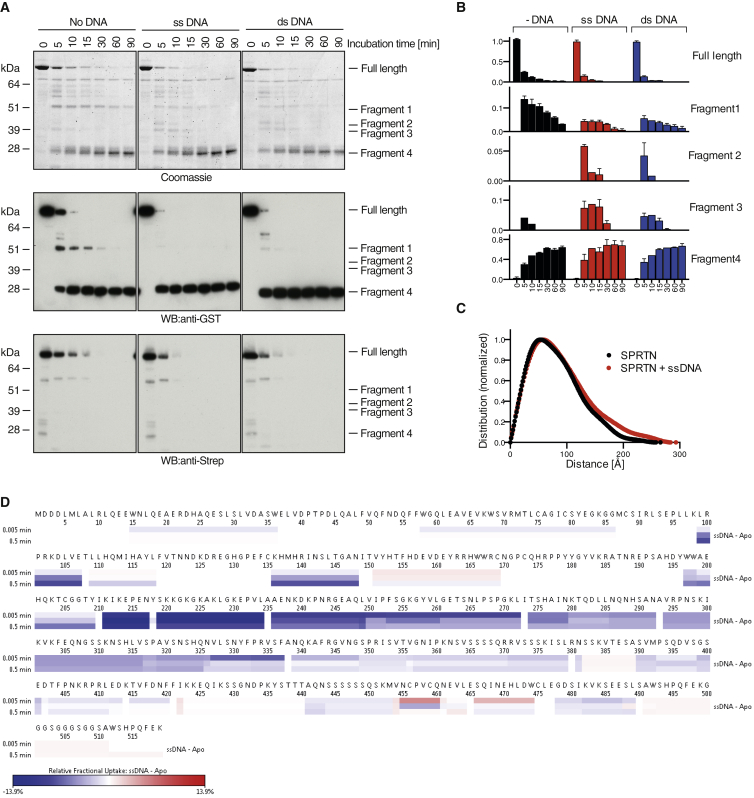
DNA Binding Induces a Conformational Change within SPRTN (A) SPRTN undergoes a conformational change upon DNA binding. Catalytically inactive GST-SPRTN-Strep E112Q was subjected to limited proteolytic digestion by trypsin in the presence or absence of ssDNA or dsDNA. (B) Quantification of specific proteolytic fragments observed in (A). Values represent mean ± SEM of three independent experiments. (C) SAXS analysis indicates that ssDNA binding increases the flexibility of SPRTN. Electron pair distribution shows an increase in Rg and Dmax upon ssDNA (15-mer) binding. (D) Heatmap showing H/D exchange mass spectrometry indicating differences in deuterium incorporation between SPRTN and SPRTN + ssDNA. Regions of increased protection are shown in blue and increased exposure in red. Deuterium labeling was carried out at three time points (0.3, 3, and 30 s) in triplicates. See also Figure S4.

The exposition of the active site within the protease fold argues that the catalytic center might require structural shielding in the context of the full-length protein in order to prohibit unwanted proteolysis. In order to test if a conformational change is involved in the DNA-dependent activation of SPRTN, we probed the overall configuration of the protease by a limited proteolysis assay in the presence or absence of DNA. Strikingly, a distinctly different cleavage pattern could be observed when catalytically inactive SPRTN-EQ was digested by trypsin in the presence of DNA (Figures 5A and 5B). In the absence of DNA, digestion resulted in the formation of one major intermediate (fragment 1). In contrast, the production and/or stability of this fragment was dramatically reduced in the presence of DNA, with two distinct intermediates (fragments 2 and 3) being formed instead. Moreover, SPRTN generally was digested quicker in the presence of DNA, indicating a generally more open conformation. Ultimately, SPRTN was degraded entirely with only the GST tag remaining (fragment 4). Interestingly, the conformational change in the presence of DNA appeared to be more complete with ssDNA compared with dsDNA.

To further characterize the conformational change of SPRTN induced by DNA, we collected small angle X-ray scattering (SAXS) data on SPRTN-EQ in the absence or presence of ssDNA (15-mer) (Figures 5C, S4A, and S4B). The data indicated that the DNA-free protein is flexible, but that the addition of DNA increases flexibility significantly. The Rg of the DNA-bound SPRTN increased by 8–10 Å and the Dmax increased by 30 Å compared to DNA-free protein. Although we cannot formally exclude the possibility that the ssDNA sticks out, it is unlikely as (1) the ssDNA is small relative to the protein, (2) the ssDNA would likely be disordered and contribute less to the SAXS signal, and (3) a minimal ssDNA was used that shows an effect nevertheless. Porod analysis of the DNA-free protein indicated significant levels of flexibility, with a Porod Exponent of 2.7 (Rambo and Tainer, 2011). In the presence of DNA, the Porod Exponent changed to 2.5, indicating an increase in flexibility. This increase in flexibility also was observed in the Dimensionless Kratky, with the ssDNA-bound protein decreasing in peak height (Figure S4C) (Reyes et al., 2014). These results are consistent with the limited proteolysis data and with a model of an opening of the enzyme upon DNA binding.

To gain further insights into the regions of SPRTN involved in the conformational change, we performed hydrogen/deuterium (H/D) exchange mass spectrometry on SPRTN-EQ in the absence and presence of ssDNA (Figures 5D and S5D). Intriguingly, the region around the DNA-binding domain (residues 200–250) became strongly protected (i.e., less exposed to the solvent) in the presence of DNA, most likely due to direct DNA binding. In addition, the active site was less exposed, probably because it engaged with a second SPRTN molecule as a substrate. In contrast, the C-terminal part of the protein tended to be rather more solvent exposed in the presence of DNA. Collectively, these results suggest that DNA binding induces a subtle but significant conformational change that enables the active site to engage with substrates.

### A Ubiquitin Switch Controls SPRTN’s Access to Chromatin

**Figure 6:**
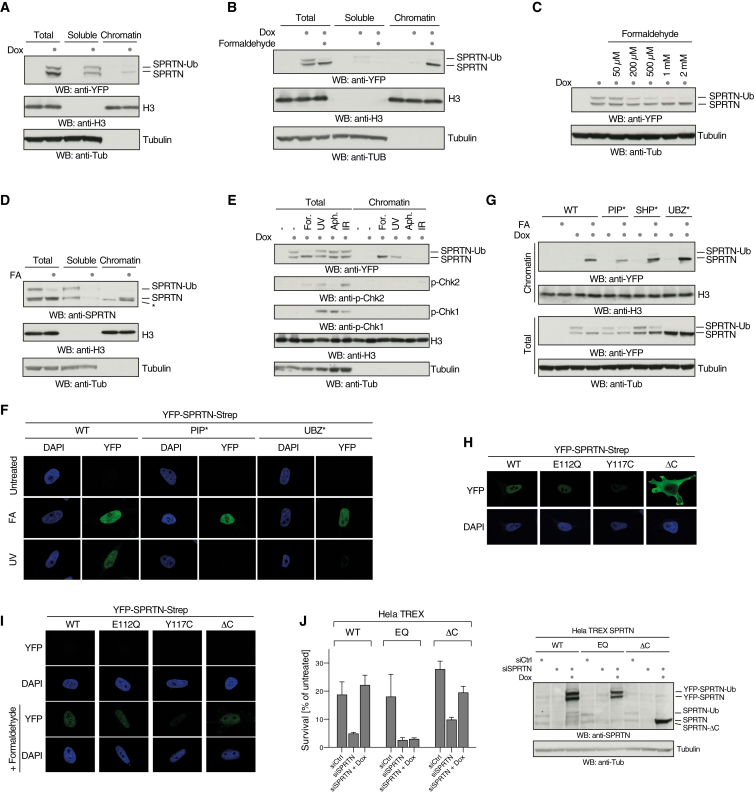
Chromatin Access of SPRTN Is Controlled by a DPC-Triggered Ubiquitin Switch (A) Mono-ubiquitinated SPRTN is excluded from chromatin. Doxycycline-inducible YFP-SPRTN-Strep HeLa Flp-In TRex cells were either lysed directly in SDS-containing loading dye (total) or subjected to fractionation in soluble and chromatin components. (B) Formaldehyde treatment induces deubiquitination of SPRTN coinciding with a complete relocalization to chromatin. Doxycycline-inducible YFP-SPRTN-Strep HeLa Flp-In TRex cells were treated with 1 mM formaldehyde (FA) for 2 hr. (C) SPRTN is deubiquitinated upon formaldehyde exposure in a dose-dependent manner. Doxycycline-inducible YFP-SPRTN-Strep HeLa Flp-In TRex cells were treated for 2 hr with the indicated dose of formaldehyde. (D) Endogenous SPRTN is deubiquitinated and relocalizes to chromatin formaldehyde exposure. U2OS cells were treated with 1 mM formaldehyde (FA) for 2 hr. Asterisk indicates an unspecific band. (E) Deubiquitination of SPRTN is specifically triggered by DNA-protein crosslinks. Doxycycline-inducible YFP-SPRTN-Strep HeLa Flp-In TRex cells were treated with formaldehyde (FA, 1 mM, 2 hr), UVC light (UV, 20 J/m^2^, 2 hr before lysis), aphidicolin (Aph, 1 μM, 2 hr), or IR (3 Gy, 2 hr before lysis). (F) SPRTN is differentially recruited to chromatin depending on the type of DNA damage. Doxycycline-induced YFP-SPRTN-Strep HeLa Flp-In TRex cells were treated with formaldehyde (FA, 0.5 mM) or UV (20 J/m^2^) and subjected to pre-extraction, prior to fixation and immunofluorescence. (G) SPRTN deubiquitination and chromatin recruitment upon DPC induction is independent of binding to PCNA, p97, or ubiquitin. Doxycycline-inducible YFP-SPRTN-Strep HeLa Flp-In TRex cells expressing the indicated SPRTN variants were treated with 1 mM formaldehyde (FA) for 2 hr. (H) SPRTN-ΔC displays an aberrant subcellular localization. Doxycycline-induced YFP-SPRTN-Strep HeLa Flp-In TRex cells were analyzed using immunofluorescence. (I) SPRTN-ΔC is recruited to chromatin upon the induction of DPCs. Doxycycline-induced YFP-SPRTN-Strep HeLa Flp-In TRex cells were treated with formaldehyde (FA, 0.5 mM) and subjected to pre-extraction, prior to fixation and immunofluorescence. (J) SPRTN-ΔC complements the formaldehyde sensitivity of SPRTN-deficient cells only partially. HeLa Flp-In TRex cells bearing the indicated doxycycline-inducible YFP-SPRTN-Strep alleles were transfected with siRNA against endogenous SPRTN and incubated in the absence or presence of doxycycline for 48 hr. Cells were then treated for 48 hr with 100 μM formaldehyde, and cell numbers were determined after an additional 4-day incubation. Values indicate cell numbers relative to untreated cells. Error bars represent SD of two to four replicates. Knockdown and doxycycline induction were confirmed by western blotting. Please note that autocleavage bands appear at similar positions as endogenous SPRTN in cells expressing WT YFP-SPRTN. See also Figure S5.

DNA binding and the associated conformational change appear to be an essential step for SPRTN activation. Thus, we sought to understand how chromatin recruitment and DNA binding are controlled in vivo. SPRTN is present in cells in two forms, unmodified and mono-ubiquitinated (Mosbech et al., 2012), and a fraction of SPRTN is constitutively present on chromatin. We noticed that chromatin-associated YFP-tagged SPRTN consists only of the unmodified species, suggesting that the mono-ubiquitination regulates chromatin binding (Figure 6A). Strikingly, DPC induction by formaldehyde resulted in an almost complete deubiquitination of SPRTN coinciding with a relocalization of the entire SPRTN pool to chromatin (Figure 6B). That the mono-ubiquitinated form was indeed deubiquitinated was indicated by the fact that the amount of unmodified SPRTN increased in correspondence to the loss of modified SPRTN (Figure S5A). Moreover, the loss of modified SPRTN could not be explained by proteasomal degradation, because it still occurred in the presence of MG132 (Figure S5B). The deubiquitination of SPRTN upon DPC induction was induced in a dose- and time-dependent manner (Figures 6C and S5C). Importantly, endogenous SPRTN also was deubiquitinated upon formaldehyde exposure, which triggered its relocalization to chromatin (Figures 6D and S5D). Notably, purified mono-ubiquitinated and unmodified YFP-tagged SPRTN displayed very similar autocleavage kinetics, indicating that the modification did not influence SPRTN’s activity (Figures S5E and S5F).

Using mass spectrometry, we identified four ubiquitination sites in SPRTN’s C terminus (lysines 341, 376, 414, and 435), which were strongly reduced upon formaldehyde treatment and absent in an SPRTN-UBZ^∗^ mutant, which lacked mono-ubiquitination (Figure S5G) (Mosbech et al., 2012). However, an SPRTN variant with these lysines mutated to arginines (SPRTN-4KR) was still mono-ubiquitinated (Figure S5H). Mutation of six further lysines in the vicinity (361, 384, 407, 423, 424, and 427) failed to abolish mono-ubiquitination. We conclude that the modification can jump to alternative lysines, suggesting that the actual site of modification is not crucial for its function. The SPRTN-10KR was unstable and expressed at low levels, which precluded additional mutational efforts (Figure S5H).

Notably, deubiquitination appears to be specific for formaldehyde treatment, as other types of DNA damage, induced by UV, IR, or aphidicolin, did not result in strong deubiquitination of SPRTN (Figure 6E). Nonetheless, unmodified SPRTN was recruited to chromatin upon UV exposure, as has been published previously (Centore et al., 2012), but not by IR or aphidicolin (Figure 6E). Interestingly, SPRTN bears several protein-protein interaction motifs in its C-terminal tail, allowing it to associate with PCNA (PIP-box), the AAA-ATPase p97 (SHP-box), and ubiquitin (UBZ) (Figure 1A). UV-induced chromatin recruitment of SPRTN depended entirely on its PIP-box and on its UBZ domain (Figures 6F and S5I) (Centore et al., 2012). In contrast, recruitment upon formaldehyde treatment appeared to be mostly independent of PCNA binding and did not require its UBZ domain (Figures 6F and 6G). This is in line with the essential cellular function of SPRTN being independent of its C-terminal region. SPRTN variants lacking the interaction motifs for PCNA, p97, or ubiquitin binding in its C terminus rescue the growth defect of *Sprtn*^−/−^ cells (Maskey et al., 2014). Moreover, patients lacking the entire C-terminal domain of SPRTN (SPRTN-ΔC) are viable, whereas a complete SPRTN knockout is lethal (Lessel et al., 2014). The SPRTN-ΔC variant displayed significantly higher expression levels and was mislocalized in cells (Figure 6H). Nonetheless, some SPRTN-ΔC could be recruited to chromatin upon formaldehyde treatment, consistent with its ability to bind DNA in vitro (Figures 6I and S5J). In agreement, SPRTN-ΔC could partially complement the formaldehyde sensitivity caused by the loss of SPRTN (Figures 6J–6L). Thus, despite lacking the protein-protein interaction motifs present in SPRTN’s C-terminal part, SPRTN-ΔC retained partial functionality, perhaps explaining the viability of the patients.

Taken together, our data reveal that SPRTN is recruited to chromatin in the presence of DPCs, a process that is tightly linked to its deubiquitination and mechanistically distinct from its recruitment to UV damage. We propose that rapid deubiquitination upon DPC induction ensures that SPRTN is only localized to chromatin when its proteolytic activity is required.

### Autocatalytic Cleavage Negatively Regulates SPRTN at Damage Sites

**Figure 7:**
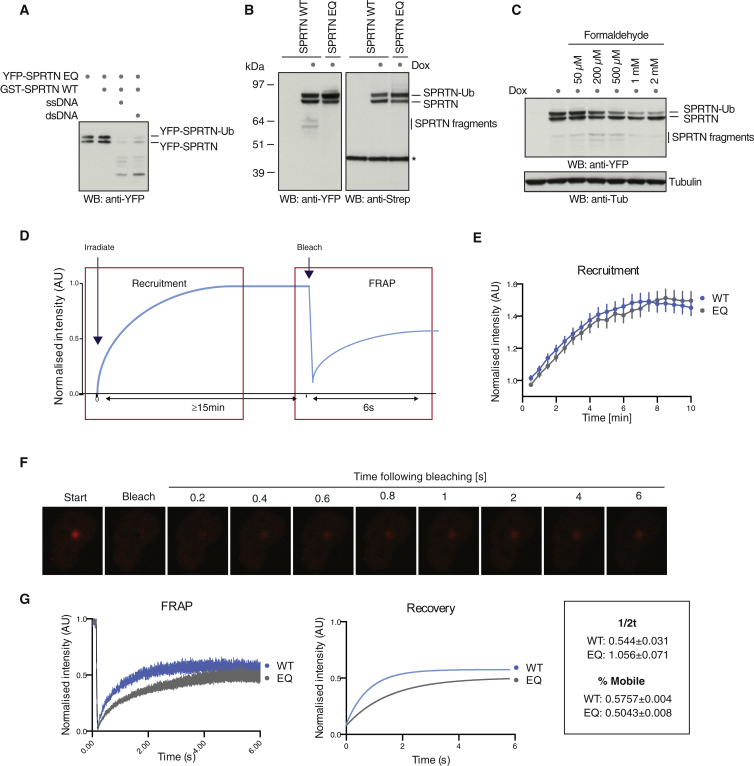
Autocleavage Controls SPRTN Dynamics at Sites of DNA Damage (A) SPRTN autocleavage occurs in *trans*. Recombinant GST-SPRTN-Strep WT and catalytically inactive YFP-E112Q-Strep were incubated in the absence or presence of ss and ds phage DNA (10 nM) for 2 hr at 25°C. (B) SPRTN autocleavage occurs in cells. Doxycycline-inducible YFP-SPRTN-Strep HeLa Flp-In TRex cells expressing the indicated SPRTN variants were lysed and subjected to SDS-PAGE followed by western blotting against the N-terminal YFP and the C-terminal Strep tag. The asterisk indicates an unspecific band serving as loading control. (C) SPRTN autocleavage is triggered by formaldehyde. Doxycycline-inducible YFP-SPRTN-Strep HeLa Flp-In TRex cells were treated with the indicated dose of formaldehyde for 2 hr. (D) Schematic representation shows laser microirradiation and fluorescence recovery after photobleaching (FRAP) experiments. (E) Recruitment of YFP-SPRTN-Strep (WT or EQ) in HeLa Flp-In TRex cells after laser microirradiation. Data are from ≥20 cells ± SEM normalized to pre-irradiation fluorescence. (F) Representative images of HeLa Flp-In TRex cells expressing WT YFP-SPRTN-Strep from FRAP time course at indicated time following bleaching. Bleaching was achieved with 0.1-s pulse of 405-nM laser (scale bar, 10 μm). (G) FRAP from HeLa Flp-In TRex cells expressing YFP-SPRTN-Strep (WT or EQ) data are from ≥15 cells ± SEM normalized to pre-bleach fluorescence (left panel). Fitted exponential fluorescence recovery of FRAP data is shown (right panel).

SPRTN’s recruitment to chromatin appears to be highly regulated, suggesting the existence of a mechanism that ensures that SPRTN is eventually turned off. Intrigued by the different properties of autocleavage and substrate cleavage by SPRTN in vitro (Figure 3), we speculated that this might have a regulatory function in vivo. First we asked whether autocleavage occurs in *trans* or in *cis.* GST-tagged WT SPRTN was able to process catalytically inactive YFP-tagged SPRTN-EQ in the presence of DNA, showing that autocleavage occurs in *trans* (Figure 7A). Notably, SPRTN-EQ was processed in the presence of ssDNA and dsDNA, indicating that this is a true autocleavage event. Next we tested whether autocleavage occurs in cells. Indeed, N-terminal fragments could be observed in cells expressing YFP-SPRTN-Strep. These fragments were absent in cells expressing a catalytically inactive SPRTN variant, suggesting that they are produced by autocatalytic cleavage (Figure 7B). Interestingly, the levels of autoproteolytic fragments increased when cells were exposed to formaldehyde, implying that autocleavage is functionally linked to DPC repair by SPRTN (Figure 7C).

To determine if autocleavage has a regulatory role, we conducted live-cell experiments to study the dynamics of SPRTN recruitment to sites of laser-inflicted DNA damage (Figure 7D), to which it previously has been shown to be recruited (Davis et al., 2012). Importantly, recruitment was independent of PCNA binding, as it was for formaldehyde-induced damage (Figure S6A). SPRTN-WT and SPRTN-EQ were recruited with identical kinetics to the site of damage (Figures 7E and S6B). However, the significantly slower recovery after bleaching revealed that SPRTN-EQ remained much more stably associated with the damage site once recruited (Figures 7F and 7G). We conclude that autocleavage plays a crucial role in removing SPRTN from sites of DNA damage, which likely restricts unwanted proteolysis on chromatin.

## Discussion

Its remarkable DNA-dependent proteolytic activity renders SPRTN ideal for efficient processing of crosslinked proteins, irrespective of their identity. Needless to say, this is a very toxic activity with the potential to degrade any chromatin protein if not properly controlled. Our structural data further highlighted the need to restrain the protease activity of the SPRTN/Wss1 family since the catalytic center is solvent exposed and bears few signs of specificity-generating features (Figure 4).

We discovered several molecular mechanisms, switches, that restrain SPRTN’s activity and, consequently, control DPC repair in metazoans. The ubiquitin switch appears to be the most upstream control mechanism of SPRTN activity. Mono-ubiquitinated SPRTN is excluded from chromatin; however, the induction of DPCs by formaldehyde triggers its deubiquitination, thus allowing chromatin relocalization. Hence, this switch regulates SPRTN by adjusting the level of chromatin-accessible SPRTN in correspondence to the amount of DPC damage. Unmodified SPRTN is able to access chromatin and bind DNA, which triggers another regulatory mechanism, the DNA switch. Intriguingly, the DNA switch can be activated in two distinct modes depending on the type of DNA to which SPRTN is bound. The dsDNA binding renders the protease active, but only with respect to autocleavage. In contrast, ssDNA binding also induces substrate cleavage. Our data indicate two means by which the DNA switch regulates SPRTN. First, DNA binding causes SPRTN to adopt a more open conformation. Notably, the structural change induced by ssDNA compared to dsDNA is more stable, which may allow SPRTN to efficiently bind and, thus, process substrate proteins. Second, DNA serves as a scaffold that brings the enzyme in close proximity to its substrate, thereby increasing the mean residence time. This allows completion of the proteolysis reaction, despite SPRTN’s low affinity toward substrates, which is underlined by the enzymes inability to cleave non-DNA-associated proteins, even if activated by DNA. Thus, the low specificity and the concomitant low affinity of the protease serve two purposes: enabling SPRTN to process a variety of substrates but also restraining unwanted proteolysis.

Finally, we identified an additional safeguarding mechanism that negatively regulates SPRTN’s activity, the autocatalytic off switch. Induction of DPCs by formaldehyde in cells not only results in SPRTN activation, as inferred from its deubiquitination and relocalization to chromatin, but also in increased autocleavage, which is crucial for the eventual release of the enzyme (Figure 7). Intriguingly, autocleavage is most apparent with intermediate doses of formaldehyde compared to high doses. In contrary, deubiquitination is induced with a linear dose response. This perhaps reflects a balance between turning the SPRTN pathway on and off. High levels of DPCs require the entire pool of SPRTN for repair and, thus, little autocleavage is observed. Intermediate levels of formaldehyde activate the pathway, but they also result in autocleavage adjusting the amount of active enzyme corresponding to the amount of DPC damage. Furthermore, dsDNA binding induces exclusively autocleavage, thus efficiently insulating undamaged chromatin to unwanted cleavage by SPRTN. Conversely, this suggests that ssDNA needs to be present at sites of DPCs in order to allow proteolysis.

Intriguingly, the two scenarios inducing replication-coupled DPC proteolysis involve ssDNA being present in close vicinity. DPCs located on the leading strand stall progression of the replicative helicase, thereby triggering DPC proteolysis (Duxin et al., 2014). Conversely, lagging strand DPCs can be bypassed by the helicase but stall DNA synthesis by the DNA polymerase, which again triggers DPC proteolysis. An important issue requiring further attention is how this stalling is signaled, resulting in the recruitment and activation of DPC proteases. Classical checkpoint signaling does not seem to be strongly involved, as formaldehyde does not induce Chk1 activation and only results in very low Chk2 phosphorylation (Figure 5). Chk2 activation is presumably triggered by double-strand breaks resulting from cleavage of DPC-stalled forks. In agreement, SPRTN deficiency in mouse cells is accompanied by Chk2, but not Chk1, activation (Maskey et al., 2014). Thus, there has to be a different signaling mechanism in place to induce the deubiquitination of SPRTN. Indeed, a specific ubiquitination signal seems to be required, as a dominant-negative ubiquitin mutant inhibits DPC repair in *Xenopus* (Duxin et al., 2014). Determining the nature of this signal together with the enzymes (E3 ligase and deubiquitinating enzyme) regulating SPRTN mono-ubiquitination will be paramount to understanding the complex signaling mechanisms orchestrating DPC repair.

Our data strongly suggest that DPC repair is the main function of SPRTN. In turn it seems likely that faulty DPC repair is the molecular defect underlying RJALS. The two reported patient alleles have differing effects on SPRTN’s activity. SPRTN-ΔC retains residual activity in vitro and in vivo, probably explaining the viability of the patients. Additionally, the loss of the C terminus seems to interfere with proper subcellular localization and regulated chromatin recruitment. The second disease variant SPRTN-Y117C is catalytically inactive in vitro and appears to be less stable, as indicated by its low expression levels in human cells. As a consequence, the patients develop early-onset hepatocellular carcinoma and severe premature aging (Lessel et al., 2014). Intriguingly, the liver is the major detoxifying organ where the bulk of metabolic processes producing reactive aldehydes occur. Thus, it seems likely that cells in the liver face significantly more DPCs compared to other tissues. Interestingly, some of the pathologies observed in RJALS are difficult to explain by a replicative role of SPRTN. The vast majority of liver cells are in a quiescent state, suggesting that few DPCs will challenge cells during replication. Moreover, premature cataract has been observed in RJALS and in a hypomorphic SPRTN mouse model, which is a general sign of failure to maintain postmitotic tissue homeostasis (Maskey et al., 2014, Ruijs et al., 2003). A replication-independent function of SPRTN also is indicated by results in flies, where SPRTN is recruited to chromatin independently of replication (Delabaere et al., 2014). Consistently, we found that arrested L1 worm larvae, in which no replication is occurring, are extremely sensitive to acute formaldehyde exposure if they lack SPRTN. It seems plausible that cells may not risk repairing DPCs exclusively in S-phase, where a failure to complete repair has dramatic consequences. However, further work will be required to elucidate the replication-independent function of SPRTN.

The fact that SPRTN is essential in mammals suggests that cells are challenged with significant levels of spontaneous DPCs at any given time. In contrast, ICLs seem to occur less frequently, as components of the FA pathway are generally dispensable for viability. Similarly, DPC repair seems to be more important than ICL repair for providing tolerance to the crosslinking compound cisplatin (Figure 1D), indicating that DPCs contribute significantly to its cytotoxic activity. Notably, cisplatin derivatives (carboplatin and oxaliplatin) are widely used to treat ovarian and colon cancer. Hence, interfering with DPC repair by inhibiting SPRTN may represent a potential therapeutic opportunity that could be exploited to sensitize quickly dividing cancer cells to chemotherapy. At any rate, the emerging data on DPC repair by the SPRTN/Wss1 DPC protease family highlight the importance of this DNA repair pathway for genome integrity and human health.

## Experimental Procedures

### DNA-Dependent Autocleavage Assays

Reactions were performed at 25°C in 20 μl containing 6 μl SPRTN (600 nM in 50 mM HEPES [pH 7.5], 250 mM NaCl, and 10% glycerol), 2 μl DNA (concentrations indicated in figure legends, in Tris-EDTA (TE) or water), and 12 μl H_2_O. Several types of DNA were used for activation: circular ssDNA (ΦX174 virion, New England Biolabs), circular dsDNA (ΦX174 RF I, New England Biolabs), and 30-bp ss and ds oligonucleotides (5′- TAGCAAGGCACTGGTAGAATTCGGCAGCGT-3′). Reactions were stopped by the addition of 4× lithium dodecyl sulfate (LDS) sample buffer (Thermo Fisher Scientific) supplemented with β-mercaptoethanol and boiling at 95°C for 5 min, resolved on 4%–12% Bis-Tris gradient gels, and stained with InstantBlue.

### DNA-Dependent Cleavage of DNA-Binding Proteins

Reactions were performed at 25°C in 20 μl containing 4 μl GST-SPRTN-Strep WT or variants (2.4 μM in 50 mM HEPES [pH 7.5], 250 mM NaCl, and 10% glycerol), 2 μl substrate (3.6 μM in 50 mM HEPES [pH 7.5], 250 mM NaCl, and 10% glycerol), 2 μl DNA (100 nM in TE), and 12 μl H_2_O. Either circular ssDNA (ΦX174 virion, New England Biolabs) or circular dsDNA (ΦX174 RF I, New England Biolabs) was used for activation. Reactions were stopped by the addition of 4× LDS sample buffer (Thermo Fisher Scientific) supplemented with β-mercaptoethanol and boiling at 95°C for 5 min, resolved on 4%–12% Bis-Tris gradient gels, and stained with InstantBlue.

## Author Contributions

J.S. and S.J.B. conceived and supervised the study and wrote the paper. J.S., R.B., and G.H. performed experiments. F.A. and M.G. performed structural analysis. S.L.M. and J.M.S. performed H/D exchange mass spectrometry. S.E.T. and J.A.T. performed SAXS analysis. G.S. generated reagents. A.B. and S.K. produced insect cell pellets.

## Figures and Tables

**Table 1:** X-Ray Data Collection and Refinement Statistics of the Wss1 Structure from *S. pombe*

	Sp_Wss1b (17–151) Anomalous	Sp_Wss1b (17–151)	Sp_Wss1b (17–151) E112Q
**Crystal Parameters**			

Space group	P2_1_2_1_2_1_	P2_1_2_1_2_1_	P2_1_
Cell constants	a = 40.1 Å	a = 40.3 Å	a = 41.2 Å
	b = 41.4 Å	b = 41.3 Å	b = 57.3 Å
	c = 68.3 Å	c = 68.5 Å	c = 50.6 Å
			β = 113.0
Wss1b/AU^a^	1	1	1

**Data Collection**			

Beam line	X06DA, SLS	X06DA, SLS	X06DA, SLS
Wavelength (Å)	1.4854	0.8	1.0
Resolution range (Å)^b^	30–1.8 (1.9–1.8)	30–1.0 (1.1–1.0)	30–1.75 (1.85–1.75)
Number of observations	75,642	545,122	72,610
Number of unique reflections^c^	15,941^d^	61,720^e^	20,977^e^
Completeness (%)^b^	95.2 (92.4)	98.7 (99.7)	95.3 (94.6)
R_merge_ (%)^b^^,^^f^	4.6 (23.6)	5.9 (39.8)	4.1 (43.1)
I/σ (I)^b^	18.7 (5.1)	21.0 (4.9)	14.1 (2.7)

**Refinement (REFMAC5)**			

Resolution range (Å)		15.0–1.0	15.0–1.75
Number of refl. working set		58,634	19,928
Number of refl. test set		3,086	1,049
Number of non-hydrogen		1,209	2,058
Number of of Ni^2+^		1	2
Solvent/ions		225	145
R_work_/R_free_ (%)^g^		0.143/0.168	0.173/0.195
RMSD bond (Å)/(°)^h^		0.009/1.4	0.005/1.0
Average B-factor (Å^2^)		11.7	39.9
Ramachandran plot (%)^i^		99.1/0.9/0.0	97.7/2.3/0.0
PDB accession code		5JIG	5LN5

Refl., reflections.

a Asymmetric unit.

b The values in parentheses for resolution range, completeness, R_merge_, and I/σ (I) correspond to the highest resolution shell.

c Data reduction was carried out with XDS and from a single crystal.

d Friedel pairs were treated as individual reflections.

e Friedel pairs were treated as identical reflections.

f R_merge_(I) = Σ_hkl_Σ_j_ | I(hkl)_j_ - < I(hkl) > | / Σ_hkl_ Σ_j_ I(hkl)_j_, where I(hkl)_j_ is the j^th^ measurement of the intensity of reflection hkl and < I(hkl) > is the average intensity.

g R = Σ_hkl_ | |F_obs_| - |F_calc_| |/Σ_hkl_ |F_obs_|, where R_free_ is calculated without a sigma cutoff for a randomly chosen 5% of reflections, which were not used for structure refinement, and R_work_ is calculated for the remaining reflections.

h Deviations from ideal bond lengths/angles (RMSD, root-mean-square deviation).

i Number of residues in favored region/allowed region/outlier region.

## Accession Numbers

The accession numbers for the structural data reported in this paper are PDB: 5JIG (Wss1 WT) and 5LN5 (Wss1 EQ).

## Supplemental Information

Document S1. Supplemental Experimental Procedures and Figures S1–S6

Document S2. Article plus Supplemental Information

## References

[bib1] Baker D.J., Wuenschell G., Xia L., Termini J., Bates S.E., Riggs A.D., O’Connor T.R. (2007). Nucleotide excision repair eliminates unique DNA-protein cross-links from mammalian cells. J. Biol. Chem..

[bib2] Barker S., Weinfeld M., Murray D. (2005). DNA-protein crosslinks: their induction, repair, and biological consequences. Mutat. Res..

[bib3] Baugh L.R. (2013). To grow or not to grow: nutritional control of development during Caenorhabditis elegans L1 arrest. Genetics.

[bib4] Centore R.C., Yazinski S.A., Tse A., Zou L. (2012). Spartan/C1orf124, a reader of PCNA ubiquitylation and a regulator of UV-induced DNA damage response. Mol. Cell.

[bib5] Davis E.J., Lachaud C., Appleton P., Macartney T.J., Näthke I., Rouse J. (2012). DVC1 (C1orf124) recruits the p97 protein segregase to sites of DNA damage. Nat. Struct. Mol. Biol..

[bib6] de Graaf B., Clore A., McCullough A.K. (2009). Cellular pathways for DNA repair and damage tolerance of formaldehyde-induced DNA-protein crosslinks. DNA Repair (Amst.).

[bib7] Delabaere L., Orsi G.A., Sapey-Triomphe L., Horard B., Couble P., Loppin B. (2014). The Spartan ortholog maternal haploid is required for paternal chromosome integrity in the Drosophila zygote. Curr. Biol..

[bib8] Duxin J.P., Dewar J.M., Yardimci H., Walter J.C. (2014). Repair of a DNA-protein crosslink by replication-coupled proteolysis. Cell.

[bib9] Friedberg E.C., Elledge S.J., Lehmann A.R., Lindahl T., Muzi-Falconi M. (2014). DNA Repair, Mutagenesis, and Other Responses to DNA Damage.

[bib10] Fu Y.V., Yardimci H., Long D.T., Ho T.V., Guainazzi A., Bermudez V.P., Hurwitz J., van Oijen A., Schärer O.D., Walter J.C. (2011). Selective bypass of a lagging strand roadblock by the eukaryotic replicative DNA helicase. Cell.

[bib11] Ghosal G., Leung J.W., Nair B.C., Fong K.W., Chen J. (2012). Proliferating cell nuclear antigen (PCNA)-binding protein C1orf124 is a regulator of translesion synthesis. J. Biol. Chem..

[bib12] Jackson S.P., Bartek J. (2009). The DNA-damage response in human biology and disease. Nature.

[bib13] Juhasz S., Balogh D., Hajdu I., Burkovics P., Villamil M.A., Zhuang Z., Haracska L. (2012). Characterization of human Spartan/C1orf124, an ubiquitin-PCNA interacting regulator of DNA damage tolerance. Nucleic Acids Res..

[bib14] Kottemann M.C., Smogorzewska A. (2013). Fanconi anaemia and the repair of Watson and Crick DNA crosslinks. Nature.

[bib15] Langevin F., Crossan G.P., Rosado I.V., Arends M.J., Patel K.J. (2011). Fancd2 counteracts the toxic effects of naturally produced aldehydes in mice. Nature.

[bib16] Lessel D., Vaz B., Halder S., Lockhart P.J., Marinovic-Terzic I., Lopez-Mosqueda J., Philipp M., Sim J.C., Smith K.R., Oehler J. (2014). Mutations in SPRTN cause early onset hepatocellular carcinoma, genomic instability and progeroid features. Nat. Genet..

[bib17] Lindahl T. (1993). Instability and decay of the primary structure of DNA. Nature.

[bib18] Machida Y., Kim M.S., Machida Y.J. (2012). Spartan/C1orf124 is important to prevent UV-induced mutagenesis. Cell Cycle.

[bib19] Maskey R.S., Kim M.S., Baker D.J., Childs B., Malureanu L.A., Jeganathan K.B., Machida Y., van Deursen J.M., Machida Y.J. (2014). Spartan deficiency causes genomic instability and progeroid phenotypes. Nat. Commun..

[bib20] Mosbech A., Gibbs-Seymour I., Kagias K., Thorslund T., Beli P., Povlsen L., Nielsen S.V., Smedegaard S., Sedgwick G., Lukas C. (2012). DVC1 (C1orf124) is a DNA damage-targeting p97 adaptor that promotes ubiquitin-dependent responses to replication blocks. Nat. Struct. Mol. Biol..

[bib21] Mullen J.R., Das M., Brill S.J. (2011). Genetic evidence that polysumoylation bypasses the need for a SUMO-targeted Ub ligase. Genetics.

[bib22] Nakano T., Morishita S., Katafuchi A., Matsubara M., Horikawa Y., Terato H., Salem A.M., Izumi S., Pack S.P., Makino K., Ide H. (2007). Nucleotide excision repair and homologous recombination systems commit differentially to the repair of DNA-protein crosslinks. Mol. Cell.

[bib23] Nakano T., Katafuchi A., Matsubara M., Terato H., Tsuboi T., Masuda T., Tatsumoto T., Pack S.P., Makino K., Croteau D.L. (2009). Homologous recombination but not nucleotide excision repair plays a pivotal role in tolerance of DNA-protein cross-links in mammalian cells. J. Biol. Chem..

[bib24] Nakano T., Ouchi R., Kawazoe J., Pack S.P., Makino K., Ide H. (2012). T7 RNA polymerases backed up by covalently trapped proteins catalyze highly error prone transcription. J. Biol. Chem..

[bib25] Nakano T., Miyamoto-Matsubara M., Shoulkamy M.I., Salem A.M., Pack S.P., Ishimi Y., Ide H. (2013). Translocation and stability of replicative DNA helicases upon encountering DNA-protein cross-links. J. Biol. Chem..

[bib26] Pommier Y. (2006). Topoisomerase I inhibitors: camptothecins and beyond. Nat. Rev. Cancer.

[bib27] Pommier Y., Huang S.Y., Gao R., Das B.B., Murai J., Marchand C. (2014). Tyrosyl-DNA-phosphodiesterases (TDP1 and TDP2). DNA Repair (Amst.).

[bib28] Rambo R.P., Tainer J.A. (2011). Characterizing flexible and intrinsically unstructured biological macromolecules by SAS using the Porod-Debye law. Biopolymers.

[bib29] Reyes F.E., Schwartz C.R., Tainer J.A., Rambo R.P. (2014). Methods for using new conceptual tools and parameters to assess RNA structure by small-angle X-ray scattering. Methods Enzymol..

[bib30] Rosado I.V., Langevin F., Crossan G.P., Takata M., Patel K.J. (2011). Formaldehyde catabolism is essential in cells deficient for the Fanconi anemia DNA-repair pathway. Nat. Struct. Mol. Biol..

[bib31] Ruijs M.W.G., van Andel R.N.J., Oshima J., Madan K., Nieuwint A.W.M., Aalfs C.M. (2003). Atypical progeroid syndrome: an unknown helicase gene defect?. Am. J. Med. Genet. A..

[bib32] Sczepanski J.T., Wong R.S., McKnight J.N., Bowman G.D., Greenberg M.M. (2010). Rapid DNA-protein cross-linking and strand scission by an abasic site in a nucleosome core particle. Proc. Natl. Acad. Sci. USA.

[bib33] Shi Y., Lan F., Matson C., Mulligan P., Whetstine J.R., Cole P.A., Casero R.A., Shi Y. (2004). Histone demethylation mediated by the nuclear amine oxidase homolog LSD1. Cell.

[bib34] Stingele J., Jentsch S. (2015). DNA-protein crosslink repair. Nat. Rev. Mol. Cell Biol..

[bib35] Stingele J., Schwarz M.S., Bloemeke N., Wolf P.G., Jentsch S. (2014). A DNA-dependent protease involved in DNA-protein crosslink repair. Cell.

[bib36] Stingele J., Habermann B., Jentsch S. (2015). DNA-protein crosslink repair: proteases as DNA repair enzymes. Trends Biochem. Sci..

[bib37] Swenberg J.A., Lu K., Moeller B.C., Gao L., Upton P.B., Nakamura J., Starr T.B. (2011). Endogenous versus exogenous DNA adducts: their role in carcinogenesis, epidemiology, and risk assessment. Toxicol. Sci..

[bib38] Zhitkovich A., Costa M. (1992). A simple, sensitive assay to detect DNA-protein crosslinks in intact cells and in vivo. Carcinogenesis.

