# Supplementary material for: Mechanism and Regulation of DNA-Protein Crosslink Repair by the DNA-Dependent Metalloprotease SPRTN
Source: Mol Cell. 2016 Nov 17;64(4):688–703. doi: 10.1016/j.molcel.2016.09.031 (PMC5128726; doi:10.1016/j.molcel.2016.09.031)
Supplement: Document S1. Supplemental Experimental Procedures and Figures S1–S6 [file mmc1.pdf]

Molecular Cell, Volume *64*

Supplemental Information

Mechanism and Regulation of DNA-Protein Crosslink Repair by the DNA-Dependent Metalloprotease SPRTN

Julian Stingele, Roberto Bellelli, Ferdinand Alte, Graeme Hewitt, Grzegorz Sarek, Sarah L. Maslen, Susan E. Tsutakawa, Annabel Borg, Svend Kjær, John A. Tainer, J. Mark Skehel, Michael Groll, and Simon J. Boulton

Figure S1 (related to Figure 1)

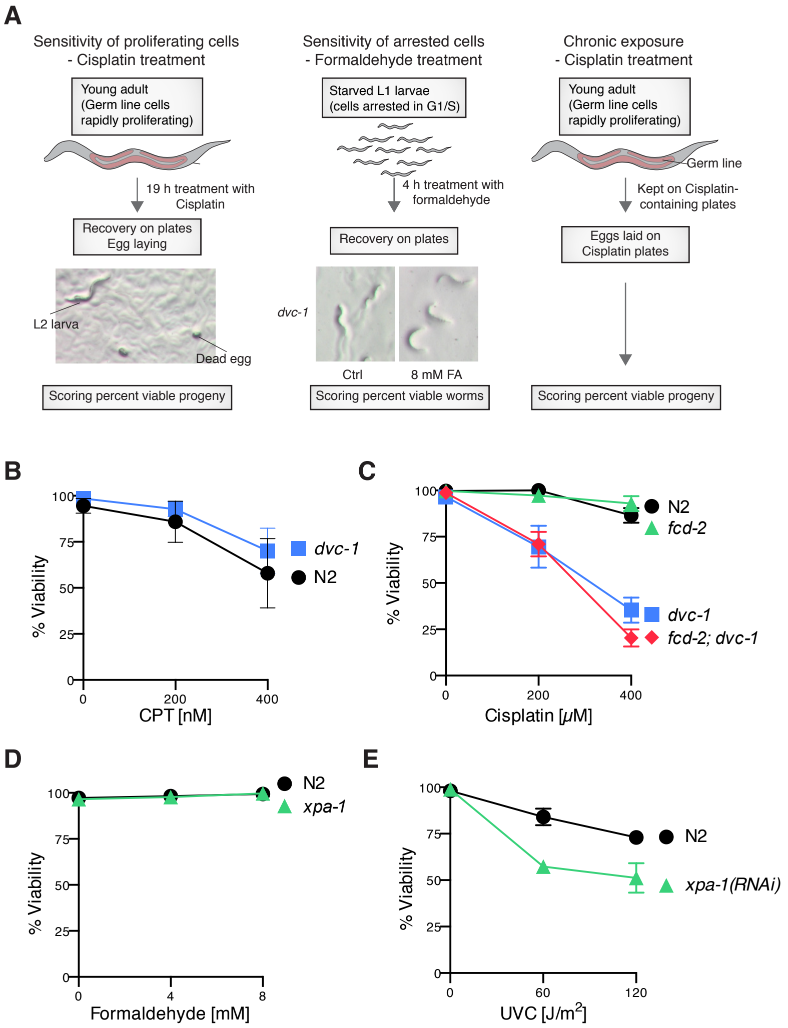

Figure S1 (related to Figure 1).

(A) Schematic representation of treatment regimens used to determine sensitivity towards DNA damage inducing agents in *C. elegans*.

(B) *C. elegans* mutant strains lacking functional SPRTN (*dvc-1*) are not sensitive towards camptothecin. Sensitivity was assessed by measuring embryonic survival of progeny after exposure of adult animals to the indicated doses. Error bars indicate SEM of two independent experiments.

(C) Progeny of FANCD2-deficient worms (*fcd-2*) do not show increased viability defects after exposure to cisplatin even in the absence of SPRTN (dvc-1) using the standard treatment regimen. Cisplatin sensitivity was assessed by measuring embryonic survival of progeny after exposure of young adult animals to the indicated doses. Error bars indicate SEM of 2 independent experiments.

(D) L1 larvae with a mutated *xpa-1* allele are not sensitive towards formaldehyde. Error bars indicated SEM of 2 independent experiments.

(E) Depletion of XPA (Xpa-1) by RNAi results in UV sensitivity in *C. elegans*. Error bars indicate SEM of 2 independent experiments.

Figure S2 (related to Figure 3)

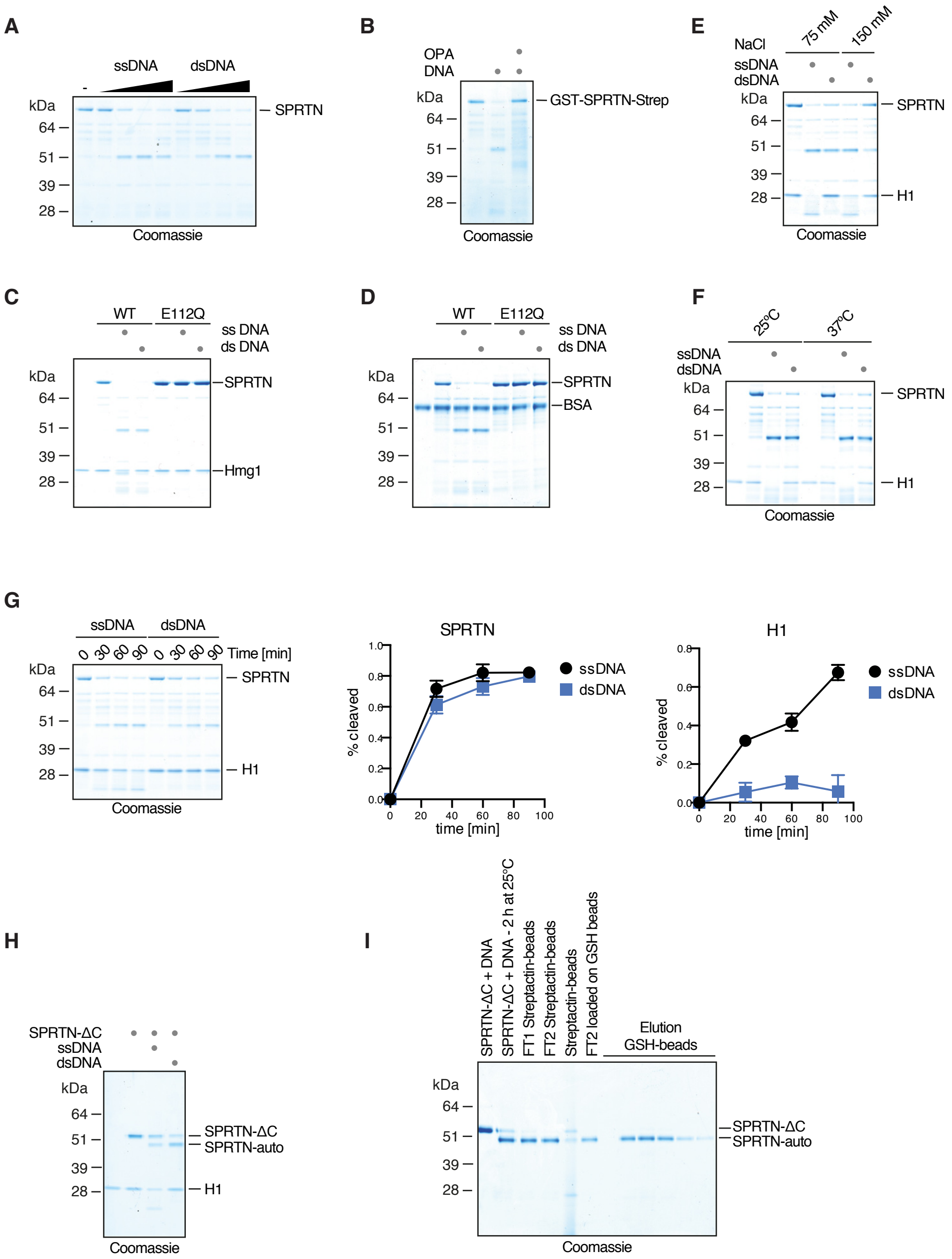

Figure S2 (related to Figure 3).

(A) Autocatalytic cleavage of SPRTN is induced similarly by single- and double-stranded DNA. Recombinant GST-SPRTN-Strep (180 nM) was incubated with different concentrations (0, 0.01, 0.1, 1 and 10 nM) of DNA (single- or double-stranded circular phage DNA). Reactions were stopped by addition of SDS-containing loading dye after a 2 h incubation at 25°C.

(B) DNA-dependent autocatalytic cleavage of SPRTN is inhibited by the metalloprotease inhibitor 1,10-phenanthroline (OPA). Recombinant GST-SPRTN-Strep (180 nM) was incubated in the absence or presence of DNA (single-stranded circular phage DNA, 10 nM) and with or without OPA (3 mM). Reactions were stopped by addition of SDS-containing loading dye after a 2 h incubation at 25°C.

(C-D) SPRTN cleaves DNA binding proteins in a single-stranded DNA-dependent manner. Recombinant GST-SPRTN-Strep (WT or the catalytically inactive E112Q variant, 480 nM) was incubated with the indicated recombinant substrates (360 nM) in the absence or presence of single- and double stranded phage DNA (10 nM). Reactions were stopped by addition of SDS-containing loading dye after a 2 h incubation at 25°C.

(E-F) Specificity of single-stranded DNA dependent substrate cleavage is independent of salt concentration and temperature. Recombinant GST-SPRTN-Strep (480 nM) was incubated with the recombinant histone H1 (360 nM) in the absence or presence of single- and double stranded phage DNA (10 nM). Reactions contained 75 mM NaCl if not indicated otherwise. Reactions were stopped by addition of SDS-containing loading dye after a 2 h incubation at 25°C (E) or in the indicated temperature (F).

(G) Kinetic analysis of auto- and substrate cleavage. Recombinant GST-SPRTN-Strep (240 nM) was incubated with the recombinant histone H1 (360 nM) in the absence or presence of single- and double stranded phage DNA (10 nM). Reactions contained 75 mM NaCl if not indicated otherwise. Reactions were stopped by addition of SDS-containing loading dye after the indicated time at 25°C. Left panel shows a representative gel; right panel shows quantification of two independent experiments.

(H) Residual substrate cleavage by SPRTN-ΔC is specifically induced by single-stranded DNA. Recombinant GST-SPRTN-Strep ΔC (480 nM) was incubated with recombinant Histone H1 (360 nM) in the absence or presence of single- and double stranded phage DNA (10 nM). Reactions were stopped by addition of SDS-containing loading dye after a 2 h incubation at 25°C.

(I) Purification of SPRTN-auto. GST-SPRTN-Strep ΔC was subjected to autocleavage by addition of DNA, which results in removal of the C-terminal Strep-tag. The reaction was stopped after 2 hours by DNA digestion through addition of micrococcal nuclease. Undigested SPRTN-ΔC was removed by passing the reaction twice over Streptactin-beads. The second flow-through (FT2) was collected and passed over GSH-beads. Finally, SPRTN-auto was eluted using reduced glutathione.

Figure S3 (related to Figure 4)

A

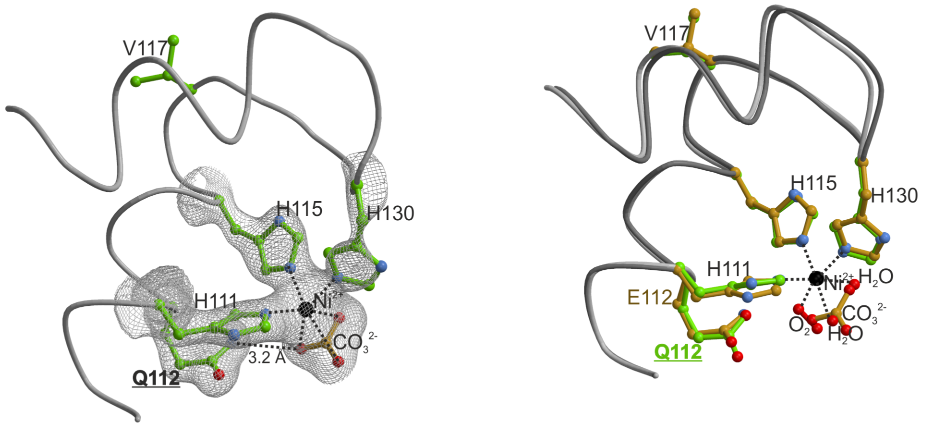

B

```
                        100        110        120        130
H. sapiens              LRPRKDLVETLLHEMIHAYLFVTNNDKDREGHGPEFCKH
M. musculus             LRPRKDLVETLLHEMIHAYLFVTNNDKDREGHGPEFCKH
D. melanogaster         LRPRKDLVETLLHEMIHAYCFVLNIREGNGGHGPNFKRI
C. elegans              LRPRSDLVETLLHEMIHAYLFVKERNRDRDGHGPQFQAH
S. pombe b              FRDYKTVKSTLIHELTHNVHG.........EHDSSFWEL
S. pombe a              WLPFEFVMDVFLHELCHIWQG.........PHDRRFFSH
S. cerevisiae           FLPMECIMGTMLHELTHNLFG.........PHDKKFYNK
A. thaliana             FLSYHEILDTMLHELCHNAHG.........PHNASFYKL
```

Figure S3 (related to Figure 4).

(A) Close-up view of the active site of the *pombe* Wss1-E112Q mutant (PDB: 5LN5) illustrated in cartoon representation. The $2F_O$-F_C-electron density map (grey) of the active site is contoured to 1σ (left panel). Carbon atoms of His111, His115, His130 as well as Val117 are displayed in green. The general architecture of the active site is unaffected by the EQ mutation, as indicated by the overlay of WT and E112Q Wss1 (right panel). Notable, in the E112Q-mutant a carbonate completes the octahedral coordination of the Ni^{2+} atom.

(B) Alignment of active site sequences of members of the Wss1/SPRTN protease familiy. Catalytic residues are in red, Tyr117, which is mutated in Ruijs-Aalfs syndrome, is shown in blue and conserved residues are shaded in grey. Numbering corresponds to the human sequence.

Figure S4 (related to Figure 5)

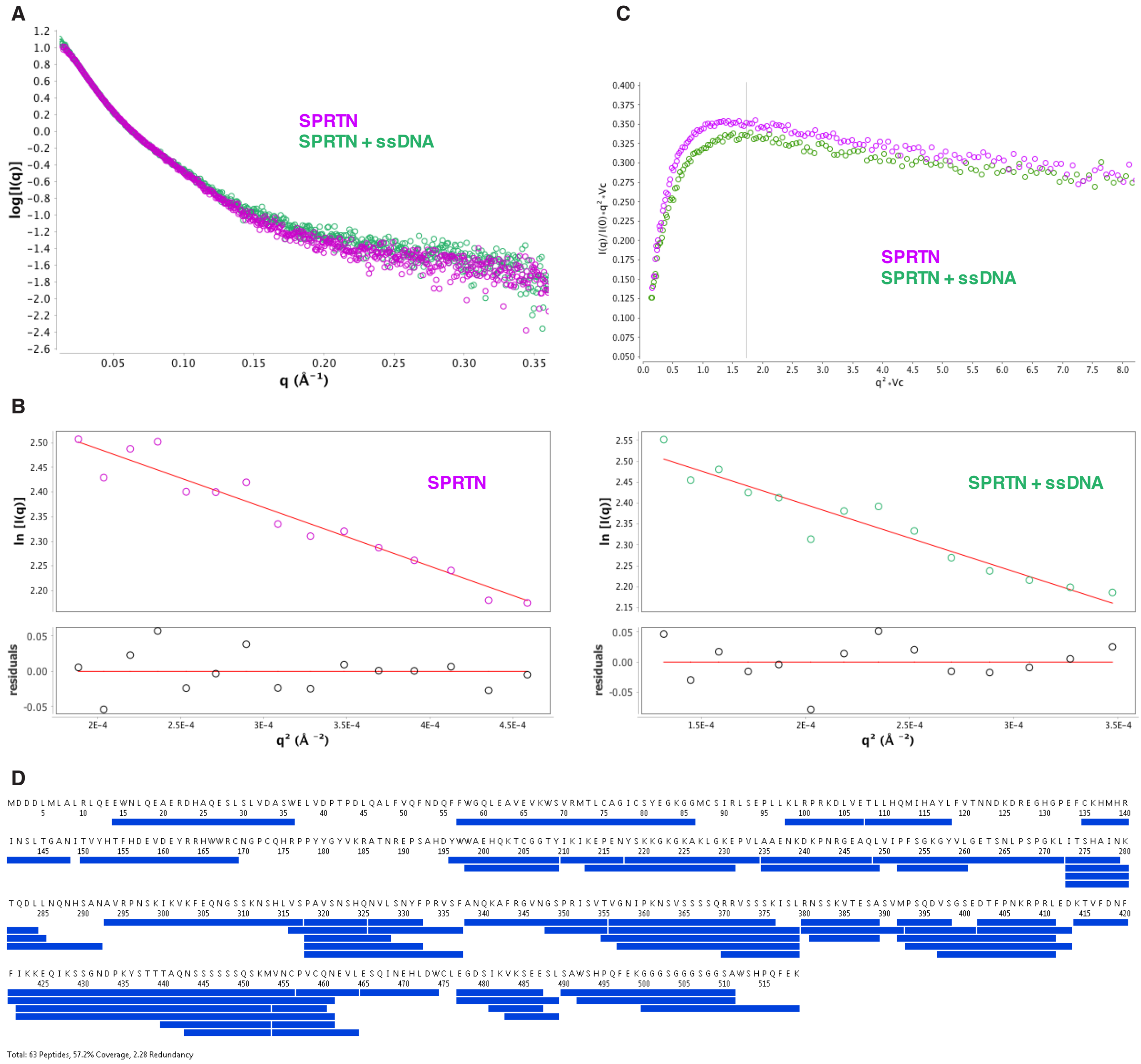

Figure S4 (related to Figure 5).

(A) SAXS curve. SAXS analysis indicates that binding of ssDNA increases the flexibility GST-SPRTN.

(B) Guinier analysis does not show aggregation.

(C) In the dimensionless Kratky, the decrease in peak height in the ssDNA-bound sample is consistent with increased flexibility.

(D) Sequence coverage of SPRTN obtained in hydrogen/deuterium exchange mass spectrometry experiments.

Figure S5 (related to Figure 6)

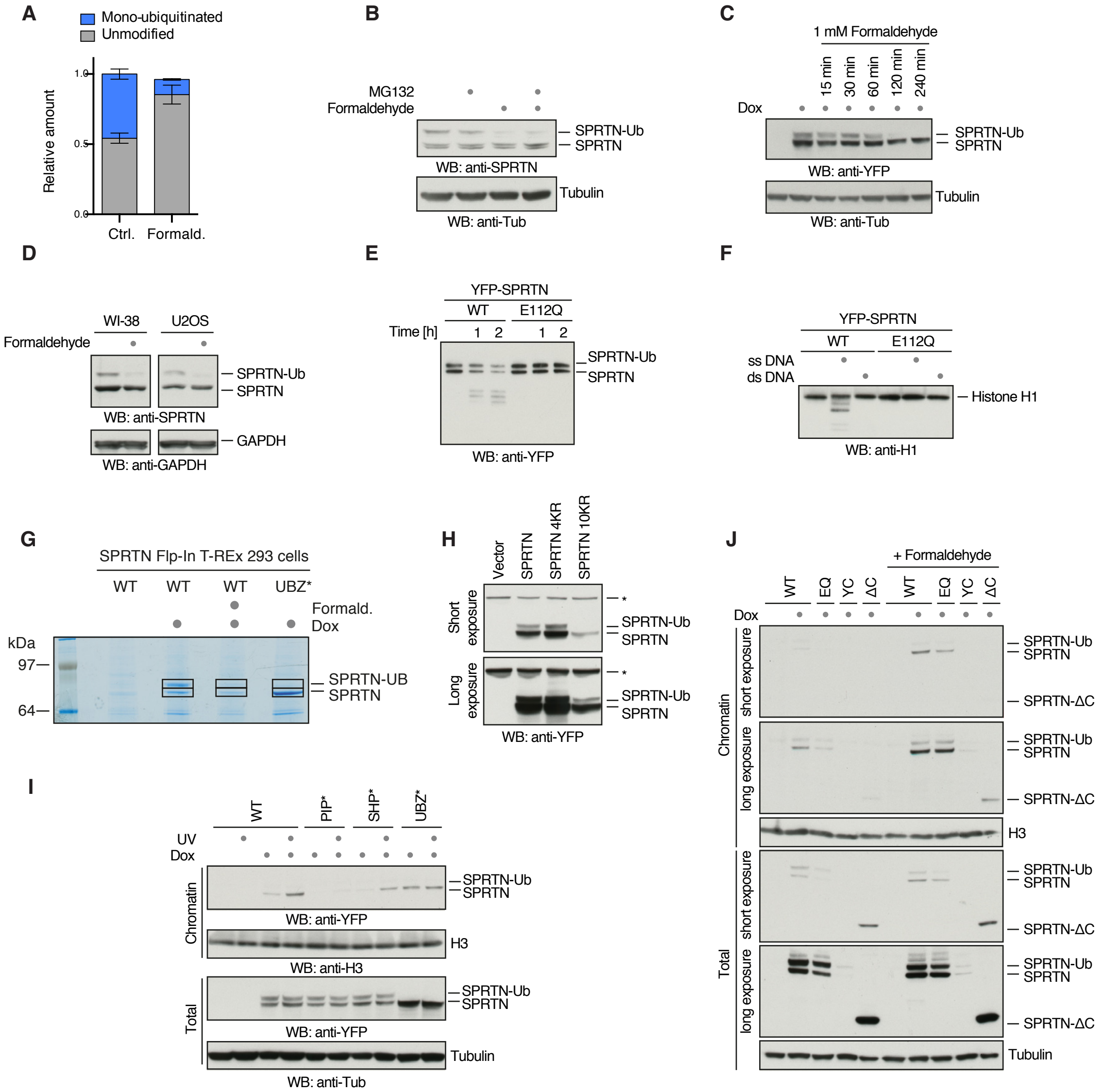

Figure S5 (related to Figure 6).

(A) Quantification of mono-ubiquitinated and unmodified SPRTN-WT before and after exposure to 1 mM formaldehyde. Western blots of three independent experiments were quantified with error bars representing SEM.

(B) SPRTN deubiquitination is independent of proteasomal degradation. U2OS cells were untreated or treated with 20 μM MG132 for 2 hours prior to addition of 1 mM formaldehyde (FA) for 2 hours. Cells were lysed directly in SDS-containing loading dye and subjected to SDS-PAGE followed by Western blotting using the indicated antibodies.

(C) SPRTN is deubiquitinated upon DPC induction by formaldehyde in a time-dependent manner. Doxycycline-inducible YFP-SPRTN-Strep HeLa Flp-In TRex cells were treated for the indicated amount of time with 1 mM formaldehyde prior to lysis in SDS-containing loading dye followed by SDS-PAGE and Western blotting using the indicated antibodies.

(D) Endogenous SPRTN is deubiquitinated upon DPC induction by formaldehyde. U2OS and WI-38 cells were treated with 1 mM formaldehyde (FA) for 2 hours. Cells were lysed directly in SDS-containing loading dye and subjected to SDS-PAGE followed by Western blotting using the indicated antibodies.

(E) Mono-ubiquitinated and unmodified SPRTN autocleave with similar kinetics. The indicated YFP-SPRTN-Strep variants were purified from 293 T-REx cells using Streptactin beads. Purified proteins were then incubated in the presence or absence of single-stranded phage DNA (10 nM) at 25ºC for the indicated amount of time. Reactions were stopped by addition of SDS-containing loading dye and analyzed by SDS-PAGE and Western blotting using the indicated antibodies.

(F) YFP-SPRTN-Strep cleaves histone H1 in a single-stranded DNA-dependent manner. Purified YFP-SPRTN-Strep, as in (E), (WT or the catalytically inactive E112Q variant) was incubated with recombinant histone H1 (100 nM) in the absence or presence of single- and double stranded phage DNA (10 nM). Reactions were stopped by addition of SDS-containing loading dye after a 4 h incubation at 25°C and analyzed by SDS-PAGE and Western blotting.

(G) Indicated YFP-SPRTN-Strep variants were purified from doxycycline-inducible YFP-SPRTN-Strep 293 Flp-In TRex using GFP-Trap agarose and subjected to mass spectrometry analysis. Deubiquitination was induced by a 2 hour formaldehyde exposure (1 mM).

(H) Analysis of SPRTN ubiquitination in KR variants. Plasmids coding for WT SPRTN or the indicated variants were transiently transfected in 293 Flp-In cells. Cells were lysed 24 hours after transfection and analyzed by Western blotting. Asterisk indicates an unspecific band serving as loading control.

(I) SPRTN's relocalization to recruitment upon UV-induced DNA damage depends on its binding to PCNA and ubiquitin. Doxycycline-inducible YFP-SPRTN-Strep HeLa Flp-In TRex cells expressing the indicated SPRTN variants were treated with UVC light (20 J/m^2) 2 hours before lysis in SDS-containing loading dye (total) or fractionation in soluble and chromatin components.

(J) SPRTN-ΔC is able to relocalize to chromatin upon DPC-induction by formaldehyde. Doxycycline-inducible YFP-SPRTN-Strep HeLa Flp-In TRex cells expressing the indicated SPRTN variants were treated with 1 mM formaldehyde (FA) for 2 hours prior to lysis in SDS-containing loading dye (total) or fractionation in soluble and chromatin components.

Figure S6 (related to Figure 7)

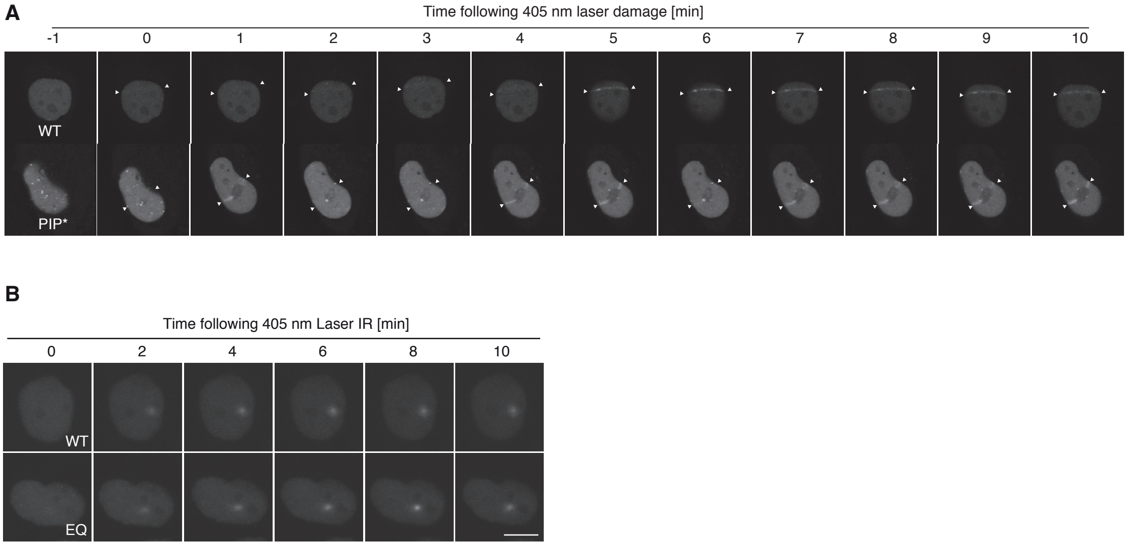

Figure S6 (related to Figure 7).

(A) Representative images of HeLa Flp-In TRex cells expressing WT or PIP* YFP-SPRTN-Strep after microirradiation (scale bar = 10 μm).

(B) Representative images of HeLa Flp-In TRex cells expressing WT or EQ YFP-SPRTN-Strep after microirradiation (scale bar = 10 μm).

EXTENDED EXPERIMENTAL PROCEDURES

C. elegans Strains and Methods

Strains were maintained using standard techniques on OP50 seeded MYOB plates supplemented with nystatin. N2 (Bristol) wild type, *fcd-2(tm1298)*, *him-6(ok412)* and *xpa-1(ok698)* mutant strains were as described previously. The *dvc-1(ok260)* mutant allele was obtained from Caenorhabditis Genetics Center, and outcrossed six times to N2 worms. All strains bearing *dvc-1(ok260)*, *fcd-2(tm1298) or him-6(ok412)* alleles were maintained as heterozygotes balanced by the *nT1(GFP)* balancer. Homozygotic mutants were selected by picking worms not expressing GFP in the pharynx.

C. elegans DNA damage sensitivity assays

Cisplatin. A cisplatin stock solution (2 mM, P4394, Sigma) was prepared in 150 mM NaCl. *Standard treatment*: Randomly picked young adult animals were treated with the indicated dose of cisplatin in 2 ml total volume M9 medium containing 300 µl of a stationary culture of OP50 bacteria at room temperature for 19 h on a slow shaker protected from light. Animals were washed three times in M9-T (M9 + 100 µl/l Triton X-100) and recovered for 1 h on OP50 seeded MYOB plates. Animals (typically 12-20 per dose and genotype) were then allowed to lay eggs on OP50 seeded MYOB plates for 4 hours. Dead eggs were counted 20 h after removing the parent animals; living animals were counted 24 h later (typically progeny of 12-15 animals was analyzed per dose and genotype). *Chronic treatment*: Randomly picked young adult animals were placed on MYOB plates containing 200 µM cisplatin or control plates (six per biological replicate). Worms were moved every 24 hours to new drug-containing plates. Embryonic survival of progeny was then determined by determining the number of hatched eggs on the 0-24, 24-48 and 48-72 hour plates.

Formaldehyde. Synchronized L1 larvae populations were obtained by bleaching gravid adult worms in M9 containing 0.5 N NaOH and 1 % sodium hypochlorite for 7 minutes. After three washes with M9, eggs were incubated in M9 for 17.5 h on a slowly rotating wheel. Synchronized L1 larvae were then splitted into several tubes and treated with the indicated dose of formaldehyde (28906, Thermofisher). After 4 hours of incubation L1 larvae were washed twice with M9 and transferred to OP50 seeded MYOB plates. Dead and living worms (typically 40-400 per dose and genotype) were scored after an incubation over night at 23 °C.

UV. Randomly picked young adult animals were exposed to the indicated dose of UVC light on OP50 seeded MYOB plates. After irradiation plates were incubated for 24 h 23 °C. Animals (typically 12-20 per dose and genotype) were then allowed to lay eggs on OP50 seeded MYOB plates for 4 hours. Dead eggs were counted 20 h after removing the parent animals; living animals were counted 24 h later (typically progeny of 12-15 animals was analyzed per dose and genotype).

Ionizing radiation. Randomly picked young adult animals were exposed to the indicated dose of ionizing radiation on OP50 seeded MYOB plates in a Cs-137 irradiator. After irradiation plates were incubated for 24 h 23 °C. Animals (typically 12-20 per dose and genotype) were then allowed to lay eggs on OP50 seeded MYOB plates for 4 hours. Dead eggs were counted 20 h after removing the parent animals; living animals were counted 24 h later (typically progeny of 12-15 animals was analyzed per dose and genotype).

Cell culture and generation of stable cell lines.

U2OS, WI-38, HeLa and MEF cells were grown in Dulbecco's modified Eagle Medium (DMEM) supplemented with 10% (v/v) fetal bovine serum (FBS), 100 Units/ml penicillin, 100 mg/mL streptomycin and 1% L-glutamine (GIBCO, Invitrogen). *Sprtn*$^{F/-}$ and *Sprtn*$^{F/+}$ MEFs were provided by Yuichi Machida (Mayo Clinic). *Fandc2*$^{-/-}$ and *Fandc2*$^{+/+}$ MEFs have been described previously (Adelman et al., 2013). HeLa and 293 cells expressing SPRTN WT and mutants were generated using the Flp-In-T-Rex system (Invitrogen) according to manufacturer's instructions and grown in DMEM supplemented with tetracycline free FBS (Clontech). Protein expression was induced by

overnight (16h) incubation with doxycycline (final concentration 1 mg/mL). Cells were transfected with Lipofectamine 2000 (Invitrogen) according to manufacturer's instructions.

Detection of formaldehyde-induced DNA-protein crosslinks

SPRTN knockout was induced in immortalized *Sprtn*$^{F/-}$ MEFs (clone H7) by treatment with 2 μM 4-hydroxy tamoxifen for 48 hours with untreated cells serving as control (Maskey et al., 2014). DPC were induced by treating cells with 200 μM formaldehyde for 1 hour. Cells were washed twice before recovery in fresh media. DPCs were measured using a KCl/SDS precipitation assay essentially as described before (Zhitkovich and Costa, 1992). To this end, cells were lysed at the respective time points by scraping in 400 μl denaturing lysis buffer (2 % SDS, 20 mM Tris/HCl pH 7.5), frozen in liquid nitrogen and stored at -80°C until further processing. After collecting all samples, lysates were thawed at 55°C for 5 min and sonicated (5 cycles, 30"/30"). Cellular protein was then precipitated by adding 400 μl 200 mM KCl, 20 mM Tris pH 7.5 and incubation on ice for 5 min. The precipitate was pelleted by centrifugation at 4°C at maximum speed using a microcentrifuge. Supernatant was saved and used for quantifying soluble DNA. The pellet was resuspended in 400 μl 200 mM KCl, 20 mM Tris pH 7.5 and resolved by shaking at 55°C for 5 min. The solution was cooled on ice for 5 min, and precipitate was again pelleted by centrifugation at maximum speed for 5 min. This wash procedure was repeated three times prior to final resuspension in 400 μl 200 mM KCl, 20 mM Tris pH 7.5. Proteins were digested by adding 0.2 mg/ml Proteinase K and incubation at 55°C for 45 min. After addition of 10 μl ultrapure BSA (50 mg/ml, Ambion), the solution was cooled on ice for 5 min followed again by centrifugation. The final supernatant, containing the initially crosslinked DNA, and the supernatant from the first wash were treated with 0.2 mg/ml RNase for 30 min at 37°C. DNA concentrations were determined using the Qubit dsDNA HS assay. The amount of DPCs was calculated as the ratio between DNA precipitated by SDS/KCl to total DNA (SDS/KCl precipitated plus soluble DNA). Relative values were obtained by subtracting basal DPC levels and normalization to values after FA addition.

Sensitivity assays mammalian cells

U2OS cells were transfected with SPRTN-specific (5'-UCAAGGAACCAGAGAAUUA-3') or control siRNA using Lipofectamine RNAimax. 48 hours after transfection cells were treated with different concentrations of DNA damaging agents in 6-well plates.

The media was exchanged 24 hours later and cells incubated for further 5 days. HeLa Flp-In TRex cells bearing the doxycycline-inducible YFP-SPRTN-Strep alleles were transfected with siRNA against endogenous SPRTN (5'- GUCAGGAAGUUCUGGUUAA-3') and incubated in the absence or presence of doxycycline for 48 hours. Cells were then treated for 48 hours with 100 μM formaldehyde and counted after an additional 4 day incubation. Cell numbers were then determined using a Countess automated cell counter.

Chromatin fractionation and immunofluorescence staining.

Chromatin fractionation experiments were performed as described before (Bellelli et al., 2014). In brief, cells in the mid-exponential phase of growth were collected by scraping in ice-cold 1X phosphate-buffered saline (PBS). Cell pellets were then directly resuspended in 1X Laemli buffer or incubated for 10 min in ice-cold CSK buffer (10 mM PIPES, pH 6.8, 100mM NaCl, 300 mM sucrose, 1mM MgCl2, 1 mM EGTA, 1mM DTT, 1 mM phenylmethylsufonyl fluoride, 10 µg/ml aprotinin) containing 0.5% Triton X-100. Chromatin-bound and soluble proteins were separated by low speed centrifugation (3,000 rpm, 3 min at 4°C). For each fraction, protein amounts deriving from comparable number of cells were analysed by SDS-PAGE and Western blotting.

For indirect immunofluorescence, cells were pre-extracted in CSK buffer containing 0.5% Triton X-100 (5 min on ice) and/or fixed in 4% paraformaldehyde, permeabilized with 0.5% Triton X-100 (5 min on ice), and then incubated with anti-GFP antibody (Abcam) for 1h at room temperature. Coverslips were washed and incubated with Alexa Fluor 488 goat anti-rabbit antibody (Invitrogen) for 30 min at room

temperature. After 5 min of DAPI counterstaining, coverslips were mounted in Glycerol/PBS (1:1) and pictures were acquired with a FV1000 Olympus confocal microscope.

Expression and Purification of Recombinant GST-SPRTN-STREP

A human SPRTN-Strep gene codon-optimized for expression in insect cells was purchased (ThermoFisher) and subcloned into pDEST20 plasmid. Viruses expressing GST-SPRTN-Strep variants were obtained using the Bac-to-Bac system. pDEST20 SPRTN plasmids were transformed into DH10Bac or DH10EMBacY *E coli* cells for transposition into the bacmid. After 48h, blue/white selection of colonies was used to identify the recombinants. The bacmids were extracted from a 2 ml overnight culture and checked by PCR. Postitive bacmids were transfected into *Spodoptera frugiperda (*Sf21) cells using Fugene HD as transfection reagent. 0.8 x 10^6 cells were plated in a 6-well plate and let to attach at 27°C for one hour. In the meantime, 500 ng of bacmid and 5 μl of Fugene HD were added to 1 ml of Sf900 III medium, free of antibiotics/supplements. The medium was removed and the DNA-lipid mix added. After 5 h at 27°C, the DNA/lipid mix was removed and 2 ml of Sf900III medium supplemented with Fungizone was added. After 3 days at 27°C, 1.5 ml of the P1 virus was added to 25 ml Sf21 culture at 10^6 cells/ml. From day 1 to day 3, the culture was monitored for signes of infection (swollen cells). The spun supernatant (P2 virus) was titered using qPCR. The P2 pellet was checked for expression of GST-SPRTN-STREP. If positive, the P2 virus was used to infect Sf21 at high density (over 5x10^6 cells/ml) at MOI 3 in Sf900III medium supplemented with Glucose, Lactalbumin and Yeastolate. After 3 days, the culture was harvested and stored at -80°C. Cell pellets were lysed on ice in 0.5 - 2 ml lysis buffer (50 mM HEPES pH 7.5, 1 M NaCl, 1 % IGEPAL CA-630, 1 mM $MgCl_2$, 10 % glycerol, 0.04 mg/ml Pefabloc SC, cOmplete EDTA-free protease inhibitor cocktail tablets (1 tablet/50 ml)) per 10^7 cells. After addition of 4 U Benzonase/ml and sonication (2 x 20 pulses) with a large flat tip using a Branson Sonifier 450 (duty cycle 80%, output control 8), lysates were incubated for 30 min on ice. Lysates, typically 45 ml, were cleared by centrifugation at 4°C and applied to batch purification columns containing 1 ml bed volume of Strep-Tactin Superflow (Qiagen) resin, which had been prewashed using 4 x 1 ml lysis buffer. The flowthrough was discarded and the beads were washed with 4 CVs lysis buffer and 2 CVs wash buffer (50 mM HEPES pH 7.5, 250 mM NaCl, 10 % glycerol). Finally, proteins were eluted using 6 x 0.5 CVs of elution buffer (50 mM HEPES pH 7.5, 250 mM NaCl, 10 % glycerol, 10 mM d-Desthiobiotin). Fraction 2-5 were pooled and dialyzed twice against 50 mM HEPES pH 7.5, 250 mM NaCl, 10 % glycerol, before freezing in liquid nitrogen and storage at -80°C. GST-SPRTN 200-250 was expressed in *E. coli* using according to standard expression and purification protocols.

Purification of GST-SPRTN-auto

GST-SPRTN-auto was generated by incubating 1 mg of recombinant GST-SPRTN-ΔC-Strep with 16 μg single stranded DNA (ΦX174 virion, NEB) at 25 °C for 2 h to induce autocleavage. DNA was then digested by addition of 16.5 U/ml Benzonase (Millipore) and MgCl2 (final concentration 1 mM). After an incubation for 20 minutes on ice NaCl was added to a final concentration of 1 M. The solution was then passed twice through 0.5 ml bead volume Strep-Tactin Superflow resin to remove uncleaved GST-SPRTN-ΔC-Strep. The second flow through was incubated with 0.25 ml Glutathione Sepharose 4 Fast Flow (GE) for 1 h at 4 °C and applied to a disposable batch purification column. The resin was washed with 50 mM HEPES pH 7.5, 250 mM NaCl, 10 % glycerol before elution of GST-SPRTN-auto with 6 times 0.125 ml elution buffer (50 mM HEPES pH 7.5, 250 mM NaCl, 10 % glycerol, 10 mM reduced glutathione). Fractions containing GST-SPRTN-auto were pooled and dialyzed twice against 50 mM HEPES pH 7.5, 250 mM NaCl, 10 % glycerol.

Expression and Purification of Recombinant Wss1 (*S. cerevisiae*)

Wss1 was purified as described previously (Stingele et al., 2014).

DNA binding assays

Electrophoretic mobility shift assays (EMSAs) were used to analyze DNA binding of recombinant proteins. *Oligos:* Proteins were prepared at different concentrations in 250 mM NaCl, 50 mM HEPES pH 7.5, 10 % glycerol and mixed with an equal volume of DNA solution (0.5 μM fluorescently-labeled DNA (Alexa488-5'- ACGCTGCCGAATTCTACCAGTGCCTTGCTA-3'), 0.2 mg/ml BSA, 15 μM HEPES pH 7.5). Following an incubation for 20 min on ice, protein-DNA complexes were resolved on 6 % retardation gels (ThermoFisher) at 80 V for 80 min at 4°C and visualized on a Typhoon imager. Contrast of scanned images was adjusted using Adobe Photoshop software. *Phage DNA:* Proteins were prepared at different concentrations in 250 mM NaCl, 50 mM HEPES pH 7.5, 10 % glycerol and mixed with an equal volume of DNA solution (50 nM single- or double stranded ΦX174 DNA in TE). Following an incubation for 20 min on ice, protein-DNA complexes were resolved on 0.8 % agarose gels (containing EtBr) at 125 V for 300 min at 4°C.

Expression and Purification of Recombinant Wss1 (*S. pombe*)

Schizosaccharomyces pombe carries two homologous *Wss1*-genes, which we termed *SpWss1a* and *SpWss1b*. SpWss1a (UniProt-ID: Q9P7B5) corresponds to Wss1 of *Saccharomyces cerevisiae*; SpWss1b (UniProt-ID: O94580) possesses an additional N-terminal ubiquitin-like domain. Both SpWss1a and SpWss1b contain a WLM-domain (Wss1 like metalloprotease). Beside the full-length genes (applied primers: Wss1_Sp_for CTAGGATCCGAGTTGAAATTTAGTTGCAGAGG; Wss1_Sp_rev CTACTGCAGTTACTCCTTTTGGACTTTACTACC), truncated constructs were amplified by PCR-techniques based on secondary structure predictions calculated with *Jalview* to remove terminal, putative flexible regions that might be deleterious for crystallization tendency. Resulting gene versions were cloned into the plasmid pRSETA_His_Tev using *Bam*HI and *Pst*I restriction sites, expressed and tested for soluble expression in the *E. coli* strain BL21 (DE3). For three liter large scale cultivations, the expression strain was grown to an OD_{600} of 0.5 to 0.7. At this stage, the temperature of the culture was adjusted to 20 °C in a cold water bath and IPTG was added to a final concentration of 0.5 mM. After overnight expression at room temperature, cells were harvested by centrifugation. Protein purifications were carried out with ÄKTA™ chromatography platforms. For this purpose, the supernatant originating from the preceding cell disruption by using a French Press System was loaded on a HisTrap™ FF Ni-NTA column. After that, the column was washed with 100 mM Tris/HCl (pH 8.0), 500 mM NaCl, 20 mM imidazole, the protein was eluted by applying a linear gradient with a final concentration of 100 % 100 mM Tris/HCl (pH 8.0), 500 mM NaCl, 500 mM imidazole. In order to increase the crystallization tendency of the target protein, the affinity tag was removed by enzymatic digestion. To this end, the protein was transferred into 10 mM Tris/HCl (pH 8.0) and TEV-protease was added to the target protein in a mass ratio of 1:100 until cleavage was completed. An additional Ni-NTA chromatography was used to separate the cleaved protein from the tag and the His-tagged TEV protease. Size exclusion chromatography (Superdex75) with 50 mM Tris/HCl pH 8.0 and 200 mM NaCl yielded pure Wss1 protein of at least 10 mg, which was stored at -20 °C for further use.

Crystallization and Structure Determination of SpWss1b (17 - 151)

Initial crystallization trials were carried out for all constructs, however, only the SpWss1b short WLM-domain (17 – 151, sequence numbering according to human SPRTN) resulted in ordered crystals. The protein concentration varied from 10-17 mg/ml and crystal droplets consisted of 1 volume of reservoir solution (100 mM Tris/HCl (pH 7.5), 20% PEG 3350) and 1 volume of protein suspension. SpWss1b (17 – 151) crystallized after few days with a typical size of about 150 × 70 × 30 μm^3, whereas crystals of the E112Q mutant (108 - 282) grew within three months. Crystals were cryoprotected by a 1:1 mixture of mother liquor and 20% (v/v) glycerol and subsequently supercooled in a stream of nitrogen gas at 100 K. The structure of Wss1 was determined by single-wavelength anomalous dispersion (SAD) at a resolution of 1.0 Å. To this end, an anomalous data set had been collected at the SLS synchrotron (Villigen, Switzerland). A fluorescence energy scan prior to anomalous data set collection at 1.8 Å resolution (Table 1) identified the central metal ion not as Zn, but as Ni. It seems very likely that the Ni-ion occurs as an artifact of the Ni-IMAC purification procedure. Data processing for SpWss1b (17 – 151) yielded the orthorhombic space group $P2_12_12_1$

with the unit cell axes of a = 40.3 Å, b = 41.3 Å, c = 68.5 Å. SHELXD located 1 strong heavy atom site and subsequent SHARP-SAD phasing as well as SOLOMON solvent flattening resulted in appropriate phases of about 1.8 Å (Bricogne et al., 2003; Sheldrick, 2010). The calculated electron density revealed well defined secondary structure elements including defined side chains, thus allowing automated protein chain tracing and model building with ARP/wARP (Langer et al., 2008). Positional refinement with REFMAC (Murshudov et al., 1997; Vagin et al., 2004) further improved phases, so that missing structural parts could be completed. Subsequently, the model was refined against the native dataset collected at 1.0 Å resolution (Table 1). Finally, Translation/Libration/Screw vibrational motion refinement yielded current crystallographic values of R_{crys} = 14.3 %, R_{free} = 16.9 %, r.m.s. bond length = 0.009 Å, and r.m.s. angles = 1.4°. The geometry of the whole molecule, including the two N-terminal amino acids (Gly and Ser), originating from the *Bam*HI restriction site, is well defined in the electron density map, except for a loop of 9 amino acids (Pro66 – Thr76; numbers according to full-length SpWss1b), which is structurally distorted. Table 1 gives an overview of the refinement statistics. Notably, His111, His115, His130, as well as 2 water molecules and 1 oxygen molecule coordinate the Ni^{2+} ion by forming a distorted octahedron. The E112Q-mutant of SpWss1b (17 – 151) crystallized in the space group $P2_1$ within 3 month. The structure was determined by molecular replacement using the coordinates of SpWss1b (17 – 151) for Patterson Search calculations at a resolution of 1.75 Å. The asymmetric unit contains 2 molecules. Final refinement yielded crystallographic values of R_{crys} = 17.3 %, R_{free} = 19.5 %, r.m.s. bond length = 0.005 Å, and r.m.s. angles = 1.0° (Table 1). Interestingly, the Ni^{2+} atom in the mutant is complexed to a CO_3^{2-}, which completes the octahedral coordination of the ion.

Analysis of conformational changes

Limited proteolysis. Conformational changes of SPRTN upon DNA-binding were determined using a limited proteolysis assay. Reactions were performed in 20 μl containing 6 μl catalytic inactive GST-SPRTN-EQ-Strep (1.2 μM in 50 mM HEPES pH 7.5, 250 mM NaCl, 10 % glycerol), 2 μl DNA (100 nM single-stranded circular DNA (ΦX174 virion, NEB) or double-stranded circular DNA (ΦX174 RF I, NEB) in TE), 10 μl H_2O and 2 μl Trypsin Gold (Promega, 5 ng/μl in 50 mM HEPS pH 7.5). Reactions were stopped at the indicated time points by addition of 4 x LDS sample buffer (ThermoFisher) supplemented with β-mercaptoethanol and boiling at 95°C for 10 min. Samples were resolved on 12% Bis-Tris gradient gels, stained with InstantBlue and scanned on a Licor Odyssey imager. Bands were quantified using ImageJ. Contrast of scanned images was adjusted using Adobe Photoshop software to highlight low abundant cleavage fragments. Alternatively, gels were subjected to western blotting with GST-or Strep-specific antibodies.

SAXS analysis. SAXS data was collected on catalytically inactive GST-SPRTN-EQ-Strep in the presence and absence of DNA. SPRTN protein was exchanged into 50 mM HEPES pH 7.5, 250 mM KCl, and 1% glycerol with a final concentration 0.7 mg/ml. For the DNA complex, SPRTN (370 ul 0.63 mg/ml protein) was mixed 1:1.2 molar ratio with 15mer ssDNA (5'-ACGCTGCCGAATTCT-3'); diluted with 2 mL 50 mM HEPES pH 7.5, 75 mM KCl, and 1% glycerol; and concentrated to a similar concentration as DNA-free SPRTN, based on final volume and I(0). Data was collected on a Pilatus 2M detector at the SIBYLS beamline 12.3.1 at the Advanced Light Source (Classen et al., 2013; Dyer et al., 2014). The sample to detector distance was 1.6 m. Data was collected at 11 keV at 10° C. Scattering data were analyzed with the program SCÅTTER (https://bl1231.als.lbl.gov/scatter/), except for the electron pair distribution plot which was calculated with the Gnom program (Svergun, 1992). For protein and protein/DNA respectively, the real space Rg was 65 and 73 Å, I(0) was 16 and 15 (arbitrary detector units), and the Dmax was 265 and 293 Å. The Guinier Rg was 59 and 69 Å and I(0) was 15 and 15 (arbitrary detector units), respectively. The molecular mass calculated from the experimental data was respectively 140 and 150 kD for protein and protein/DNA, within 20% error with the GST fusion (theoretical molecular weight 85 kD) dimerizing and in the case of the complex, a dimer with two 4.5 kD ssDNA. The Porod exponent was 2.7 for DNA-free protein and 2.5 for DNA-bound protein.

Hydrogen/deuterium exchange mass spectrometry. Deuterium exchange reactions of SPRTN were initiated by diluting the protein in D_2O (99.8% D_2O ACROS, Sigma, UK) in 50 mM Tris, 250mM NaCl,1mM TCEP pH 7.5 buffer to give a final D_2O percentage of 90%. For all experiments, deuterium labelling was carried out at 23°C (unless otherwise stated) at three time points (3 sec on ice (0.3 sec), 3 sec, and 30 sec in triplicate. The labelling reaction was quenched by the addition of chilled 2.4% v/v formic acid in 2 M guanidinium hydrochloride and immediately frozen in liquid nitrogen. Samples were stored at -80°C prior to analysis. The quenched protein samples were rapidly thawed and subjected to proteolytic cleavage by pepsin followed by reversed phase HPLC separation. Briefly, the protein was passed through an Enzymate BEH immobilized pepsin column, 2.1 x 30 mm, 5 μm (Waters, UK) at 200 μL/min for 2 min and the peptic peptides trapped and desalted on a 2.1 x 5 mm C18 trap column (Acquity BEH C18 Van-guard pre-column, 1.7 μm, Waters, UK). Trapped peptides were subsequently eluted over 12 min using a 5-36% gradient of acetonitrile in 0.1% v/v formic acid at 40 μL/min. Peptides were separated on a reverse phase column (Acquity UPLC BEH C18 column 1.7 μm, 100 mm x 1 mm (Waters, UK). Peptides were detected on a SYNAPT G2-Si HDMS mass spectrometer (Waters, UK) acquiring over a m/z of 300 to 2000, with the standard electrospray ionization (ESI) source and lock mass calibration using [Glu1]-fibrino peptide B (50 fmol/μL). The mass spectrometer was operated at a source temperature of 80°C and a spray voltage of 2.6 kV. Spectra were collected in positive ion mode. Peptide identification was performed by MS^e (Silva et al., 2005) using an identical gradient of increasing acetonitrile in 0.1% v/v formic acid over 12 min. The resulting MS^e data were analyzed using Protein Lynx Global Server software (Waters, UK) with an MS tolerance of 5 ppm. Mass analysis of the peptide centroids was performed using DynamX sotware (Waters, UK). Only peptides with a score >6.4 were considered. The first round of analysis and identification was performed automatically by the DynamX software, however, all peptides (deuterated and non-deuterated) were manually verified at every time point for the correct charge state, presence of overlapping peptides, and correct retention time. Deuterium incorporation was not corrected for back-exchange and represents relative, rather than absolute changes in deuterium levels. Changes in H/D amide exchange in any peptide may be due to a single amide or a number of amides within that peptide. All time points in this study were prepared at the same time and individual time points were acquired on the mass spectrometer on the same day.

Laser microirradiation induced DNA damage

In order to detect local recruitment of SPRTN to laser induced DPCs YFP-SPRTN-Strep-expressing HeLa Flp-In TRex cells were seeded on 35 mm glass bottom dish (Ibidi, 81158). Cells were pre-sensitized for 48h with 10 μM BrdU and expression of YFP-tagged protein was induced with 24h of 1 μg/ml doxycycline. Immediately prior to imaging DMEM was exchanged for C02 independent media (ThermoFisher Scientific #18045-045). Cells were transferred to Olympus FV1000 confocal LSM with heated stage. Laser microirradition was performed with a 405 nm laser focused through 40x objective (400mW at objective, 50 scans). Time-course was acquired on the same system, imaging every 30s for 10 min.

FRAP

DNA damage was generated using laser microirradiation on an Olympus FV1000 confocal LSM as described above. FRAP experiments were performed on damage sites after initial recruitment had plateaued ≥15 min following induction. FRAP and image acquisition was performed on the same system. Bleaching was performed following 15 frames of pre-bleach acquisition with a 0.1 s pulse from 405 nm Laser, images were acquired for a further 600 frames (1 frame = 0.01 s). Analysis was performed on ≥15 cells using FRAP profiler plugin for imageJ (http://rsb.info.nih.gov/ij/). 1/2t and %mobile were calculated using GraphPad prism.

Antibodies ab76949

Polyclonal anti-SPRTN antibody was a gift from John Rouse, anti-GST (ab92), anti-H3 (ab10799), anti-GAPDH (ab8245), anti-H1.10 (ab11079), anti-H2B (ab1790) and anti-Strep (ab76949) antibodies were purchased from Abcam, anti-Tub (T6074) and anti-GFP (11814460001, used for detection of

YFP) (11079) from Sigma, anti-Chk2 (phospho-T68, 2661S) from NEB, anti-Chk1 (phospho-S345, 2348L) from Cell Signaling, anti-H2A (07-146) from Millipore.

SUPPLEMENTARY REFERENCES

Adelman, C.A., Lolo, R.L., Birkbak, N.J., Murina, O., Matsuzaki, K., Horejsi, Z., Parmar, K., Borel, V., Skehel, J.M., Stamp, G., *et al.* (2013). HELQ promotes RAD51 paralogue-dependent repair to avert germ cell loss and tumorigenesis. Nature *502*, 381-384.

Bellelli, R., Castellone, M.D., Guida, T., Limongello, R., Dathan, N.A., Merolla, F., Cirafici, A.M., Affuso, A., Masai, H., Costanzo, V., *et al.* (2014). NCOA4 transcriptional coactivator inhibits activation of DNA replication origins. Molecular cell *55*, 123-137.

Bricogne, G., Vonrhein, C., Flensburg, C., Schiltz, M., and Paciorek, W. (2003). Generation, representation and flow of phase information in structure determination: recent developments in and around SHARP 2.0. Acta Crystallographica Section D: Biological Crystallography *59*, 2023-2030.

Classen, S., Hura, G.L., Holton, J.M., Rambo, R.P., Rodic, I., McGuire, P.J., Dyer, K., Hammel, M., Meigs, G., Frankel, K.A., *et al.* (2013). Implementation and performance of SIBYLS: a dual endstation small-angle X-ray scattering and macromolecular crystallography beamline at the Advanced Light Source. J Appl Crystallogr *46*, 1-13.

Dyer, K.N., Hammel, M., Rambo, R.P., Tsutakawa, S.E., Rodic, I., Classen, S., Tainer, J.A., and Hura, G.L. (2014). High-throughput SAXS for the characterization of biomolecules in solution: a practical approach. Methods in molecular biology *1091*, 245-258.

Langer, G., Cohen, S.X., Lamzin, V.S., and Perrakis, A. (2008). Automated macromolecular model building for X-ray crystallography using ARP/wARP version 7. Nat Protoc *3*, 1171-1179.

Maskey, R.S., Kim, M.S., Baker, D.J., Childs, B., Malureanu, L.A., Jeganathan, K.B., Machida, Y., van Deursen, J.M., and Machida, Y.J. (2014). Spartan deficiency causes genomic instability and progeroid phenotypes. Nature communications *5*, 5744.

Murshudov, G.N., Vagin, A.A., and Dodson, E.J. (1997). Refinement of macromolecular structures by the maximum-likelihood method. Acta Crystallogr D Biol Crystallogr *53*, 240-255.

Sheldrick, G.M. (2010). Experimental phasing with SHELXC/D/E: combining chain tracing with density modification. Acta Crystallogr D Biol Crystallogr *66*, 479-485.

Silva, J.C., Denny, R., Dorschel, C.A., Gorenstein, M., Kass, I.J., Li, G.Z., McKenna, T., Nold, M.J., Richardson, K., Young, P., *et al.* (2005). Quantitative proteomic analysis by accurate mass retention time pairs. Anal Chem *77*, 2187-2200.

Stingele, J., Schwarz, M.S., Bloemeke, N., Wolf, P.G., and Jentsch, S. (2014). A DNA-Dependent Protease Involved in DNA-Protein Crosslink Repair. Cell *158*, 327-338.

Svergun, D. (1992). Determination of the regularization parameter in indirect-transform methods using perceptual criteria. Journal of applied crystallography *25*, 495-503.

Vagin, A.A., Steiner, R.A., Lebedev, A.A., Potterton, L., McNicholas, S., Long, F., and Murshudov, G.N. (2004). REFMAC5 dictionary: organization of prior chemical knowledge and guidelines for its use. Acta Crystallogr D Biol Crystallogr *60*, 2184-2195.
Zhitkovich, A., and Costa, M. (1992). A simple, sensitive assay to detect DNA-protein crosslinks in intact cells and in vivo. Carcinogenesis *13*, 1485-1489.
