# Supplementary material for: Mechanism and Regulation of DNA-Protein Crosslink Repair by the DNA-Dependent Metalloprotease SPRTN
Source: Mol Cell. 2016 Nov 17;64(4):688–703. doi: 10.1016/j.molcel.2016.09.031 (PMC5128726; doi:10.1016/j.molcel.2016.09.031)
Supplement: Document S2. Article plus Supplemental Information [file mmc2.pdf]

Article

Molecular Cell

Mechanism and Regulation of DNA-Protein Crosslink Repair by the DNA-Dependent Metalloprotease SPRTN

Graphical Abstract

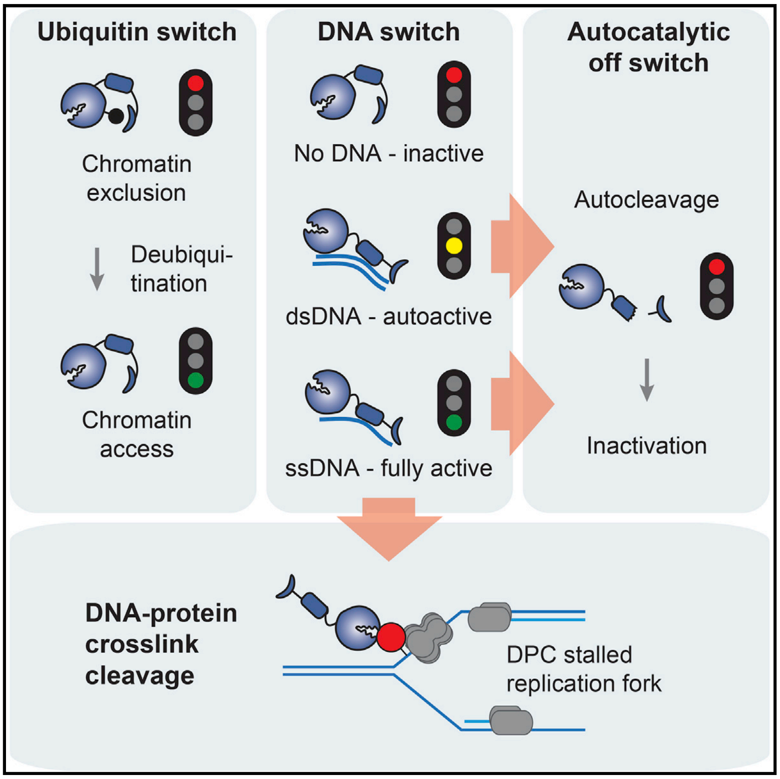

Authors

Julian Stingele, Roberto Bellelli, Ferdinand Alte, ..., J. Mark Skehel, Michael Groll, Simon J. Boulton

Correspondence

simon.boulton@crick.ac.uk

In Brief

Stingele et al. discover the SPRTN metalloprotease to be crucial for DNA-protein crosslink repair in higher eukaryotes. In addition, several regulatory principles constraining SPRTN's potentially toxic activity are described: a ubiquitin switch controlling chromatin access, a DNA switch triggering protease activity, and a negative feedback loop based on autocatalytic cleavage.

Highlights

- The SPRTN metalloprotease repairs DNA-protein crosslinks
- A DNA switch controls SPRTN's protease activity
- A ubiquitin switch controls chromatin access of SPRTN
- Structural insights reveal unique features of the SPRTN/Wss1 protease family

Accession Numbers

5JIG
5LN5

Stingele et al., 2016, Molecular Cell *64*, 688–703
November 17, 2016 © 2016 The Author(s). Published by Elsevier Inc.
http://dx.doi.org/10.1016/j.molcel.2016.09.031

Mechanism and Regulation of DNA-Protein Crosslink Repair by the DNA-Dependent Metalloprotease SPRTN

Julian Stingele,[1] Roberto Bellelli,[1] Ferdinand Alte,[2] Graeme Hewitt,[1] Grzegorz Sarek,[1] Sarah L. Maslen,[3] Susan E. Tsutakawa,[4] Annabel Borg,[1] Svend Kjær,[1] John A. Tainer,[4,5] J. Mark Skehel,[3] Michael Groll,[2] and Simon J. Boulton[1,6,*]
[1]The Francis Crick Institute, 1 Midland Road, London NW1 1AT, UK
[2]Center for Integrated Protein Science at the Department Chemie, Lehrstuhl für Biochemie, Technische Universität München, Lichtenbergstrasse 4, 85747 Garching, Germany
[3]MRC Laboratory of Molecular Biology, Francis Crick Avenue, Cambridge CB2 0QH, UK
[4]Molecular Biophysics and Integrated Bioimaging, Lawrence Berkeley National Laboratory, Berkeley, CA 94720, USA
[5]Department of Molecular and Cellular Oncology, The University of Texas M.D. Anderson Cancer Center, Houston, TX 77030, USA
[6]Lead Contact
*Correspondence: simon.boulton@crick.ac.uk
http://dx.doi.org/10.1016/j.molcel.2016.09.031

SUMMARY

Covalent DNA-protein crosslinks (DPCs) are toxic DNA lesions that interfere with essential chromatin transactions, such as replication and transcription. Little was known about DPC-specific repair mechanisms until the recent identification of a DPC-processing protease in yeast. The existence of a DPC protease in higher eukaryotes is inferred from data in *Xenopus laevis* egg extracts, but its identity remains elusive. Here we identify the metalloprotease SPRTN as the DPC protease acting in metazoans. Loss of SPRTN results in failure to repair DPCs and hypersensitivity to DPC-inducing agents. SPRTN accomplishes DPC processing through a unique DNA-induced protease activity, which is controlled by several sophisticated regulatory mechanisms. Cellular, biochemical, and structural studies define a DNA switch triggering its protease activity, a ubiquitin switch controlling SPRTN chromatin accessibility, and regulatory autocatalytic cleavage. Our data also provide a molecular explanation on how SPRTN deficiency causes the premature aging and cancer predisposition disorder Ruijs-Aalfs syndrome.

INTRODUCTION

The integrity of DNA is constantly challenged by structural and chemical alterations (Lindahl, 1993). Hence, restoration of the native DNA sequence and structure by damage-specific repair mechanisms is essential to ensuring genome stability. Germline mutations in crucial DNA repair enzymes result in several premature aging and cancer predisposition syndromes, highlighting the fundamental importance of DNA repair in mammals (Jackson and Bartek, 2009). DNA lesions range from abasic sites, small and bulky adducts, to single- and double-strand breaks, which are repaired by lesion-specific and generally well-understood mechanisms (Friedberg et al., 2014). However, specific repair mechanisms for one particular type of lesion, covalent DNA-protein crosslinks (DPCs), have remained elusive. This is despite DPCs being extremely toxic as they directly block essential chromatin transactions, such as replication and transcription (Fu et al., 2011; Nakano et al., 2012, 2013).

Covalent crosslinking of proteins to DNA can be caused by various exogenous agents, such as ionizing radiation (IR), UV light, certain metal ions, and, importantly, platinum-based chemotherapeutics such as cisplatin and derivatives (Barker et al., 2005; Stingele and Jentsch, 2015). Moreover, DPCs are induced by endogenously produced reactive metabolites, such as formaldehyde or acetaldehyde. Notably, formaldehyde is directly produced within chromatin as a by-product of the histone demethylation reaction (Shi et al., 2004; Swenberg et al., 2011). Furthermore, abasic sites bear an aldehyde group that efficiently reacts with nucleosome proteins, thereby forming DPCs (Sczepanski et al., 2010). DPCs also are produced enzymatically by the entrapment of normally transient covalent DNA-protein reaction intermediates of enzymes, such as topoisomerases 1 and 2 (TOP1 and TOP2). Distortions within the DNA (e.g., caused by nearby DNA damage) or small molecules such as camptothecin (CPT) or etoposide inhibit religation and result in stable DPC formation (Pommier, 2006). These enzymatic DPCs can be reversed by specific tyrosil-DNA phosphodiesterases (TDP1 and TDP2 acting on TOP1- and TOP2- adducts, respectively), which hydrolyze the covalent bond between the topoisomerase's active site residue and the DNA (Pommier et al., 2014). Apart from these unique cases of enzymatic DPCs, it has been suggested that DPCs are generally repaired by canonical DNA repair pathways, such as nucleotide excision repair and homologous recombination (Baker et al., 2007; de Graaf et al., 2009; Nakano et al., 2007, 2009).

Very recently a protease-based DPC repair mechanism was discovered in budding yeast (Stingele et al., 2014) and in

 © 2016 The Author(s). Published by Elsevier Inc.
This is an open access article under the CC BY license (http://creativecommons.org/licenses/by/4.0/).

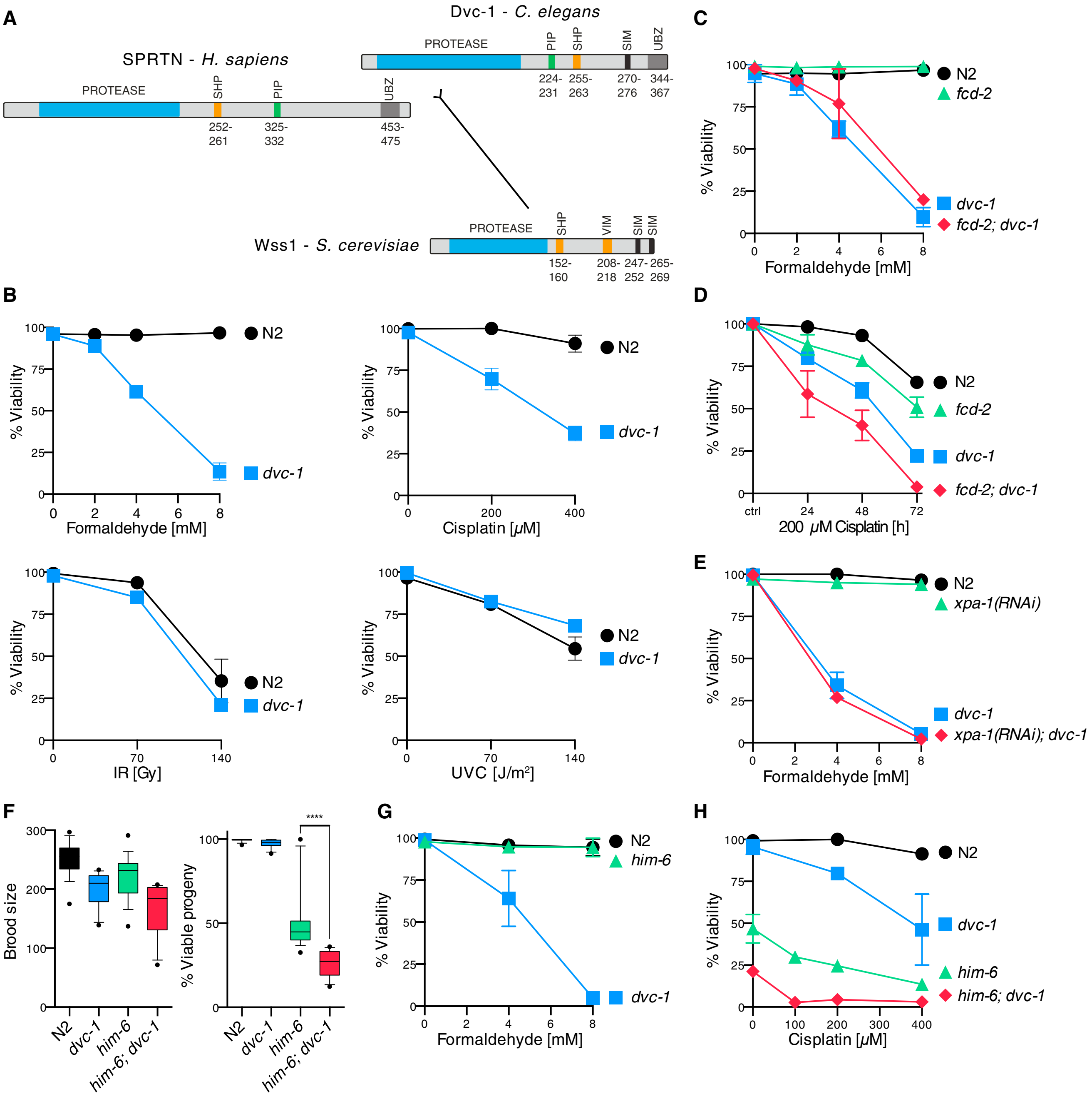

Figure 1. SPRTN/Dvc-1 Provides Resistance toward DPC-Inducing Agents in Worms and Operates Independently of FANCD2/Fcd-2 and Parallel to BLM/Him-6

(A) Domain structures and evolutionary distances of the protease domain of SPRTN/Wss1 protease family members of humans, worms, and budding yeast. SPRTN/Wss1 proteases bear interaction domains for p97/Cdc48 (SHP-box, VIM), recognition modules for ubiquitin (UBZ) or SUMO (SIM), and in metazoans a PCNA-interaction motif (PIP-box).

(B) *C. elegans* mutant strains lacking functional SPRTN (*dvc-1*) are specifically sensitive to the DPC-inducing agents. Formaldehyde sensitivity was determined in synchronized L1 larvae. Cisplatin, UVC light, and IR sensitivities were assessed by measuring embryonic survival of progeny after exposure of adult animals. Error bars indicate SEM of –two to four independent experiments.

(C) FANCD2 is not involved in providing formaldehyde resistance in synchronized L1 larvae. Error bars indicate SEM of two independent experiments.

(D) FANCD2 provides resistance to chronic cisplatin exposure by a mechanism distinct to DPC repair by SPRTN. Viability was assessed by determination of embryonic survival of progeny of young adult animals kept on cisplatin-containing plates (200 μM) for the indicated amount of time. Error bars indicate SEM of two independent experiments.

(E) Loss of XPA does not result in increased formaldehyde sensitivity in synchronized L1 worms. Error bars indicate SEM of two independent experiments.

(legend continued on next page)

Xenopus laevis egg extracts (Duxin et al., 2014). In yeast, DPC proteolysis is catalyzed by the metalloprotease Wss1, which permits replication in the presence of DPCs and provides resistance toward DPC-inducing agents. Intriguingly, Wss1 is a DNA-dependent protease that degrades DNA-bound substrates in vitro irrespective of identity. Importantly, in *Xenopus* egg extracts, a DPC-containing plasmid is repaired by a similar mechanism, indicating that protease-based DPC repair is conserved. However, the identity of the DPC protease operating in higher eukaryotes has remained elusive.

Spartan (SPRTN, DVC1) is distantly related to yeast Wss1, displays a similar domain organization, and shares a common evolutionary origin (Stingele et al., 2015). Germline mutations of *SPRTN* are causative for Ruijs-Aalfs syndrome (RJALS), which is characterized by genome instability, premature aging, and early-onset hepatocellular carcinoma (Lessel et al., 2014). Mice deficient for SPRTN are embryonically lethal, and hypomorphic mutant animals display hallmarks of premature aging and genome instability (Maskey et al., 2014). While SPRTN is clearly essential for genome stability, its molecular function remains unclear. Initial studies suggested that SPRTN is important for regulating translesion synthesis (TLS), although with conflicting reports on the actual molecular mechanism (Centore et al., 2012; Davis et al., 2012; Ghosal et al., 2012; Juhasz et al., 2012; Machida et al., 2012; Mosbech et al., 2012). Importantly, however, the severe phenotypes observed in flies, mice, and human cells have been shown to be unrelated to TLS, suggesting that SPRTN maintains genome integrity by an unknown mechanism distinct from TLS (Delabaere et al., 2014; Lessel et al., 2014; Maskey et al., 2014).

Here we identify SPRTN as the elusive DPC protease in higher eukaryotes. Using cellular, biochemical, and structural data, we establish the mechanism of SPRTN's DNA-dependent proteolytic activity, and we identify several safeguarding mechanisms that act to constrain SPRTN's potentially toxic activity, including a ubiquitin switch regulating its chromatin accessibility and a negative feedback loop based on autocatalytic cleavage.

RESULTS

SPRTN-Deficient Worms Are Hypersensitive to DPC-Inducing Agents

The SPRTN metalloprotease is essential for viability in mammalian cells, which complicates the analysis of its precise molecular function. Despite being closely related to the mammalian enzyme (Figure 1A; evolutionary distances from Stingele et al., 2015), the nematode ortholog of SPRTN (called Dvc-1) is dispensable for viability (Mosbech et al., 2012). Thus, we set out to investigate a potential role for SPRTN in DPC repair and its interaction with canonical DNA repair pathways in worms by assessing sensitivity to DPC-inducing agents. DNA damage sensitivity is typically measured in worms by treating young adult animals, followed by determining viability of their progeny as a proxy for repair defects in the germline, which is the only proliferating tissue in adult animals (Figure S1A). However, this treatment regimen is not suitable for testing formaldehyde sensitivity, because treated adult worms succumb to death at doses that have no effect on progeny viability; this is likely due to formaldehyde being unable to penetrate into the germline, similar to what has been observed with mitomycin C. Thus, we employed an alternative protocol that determines sensitivity by exposing young L1 larvae arrested by starvation (Figure S1A).

Strikingly, SPRTN-deficient L1 larvae (*dvc-1*) were extremely sensitive to an acute exposure to formaldehyde when compared to wild-type (WT) (N2) controls (Figure 1B). Furthermore, SPRTN-deficient worms (*dvc-1*) were very sensitive to cisplatin (induces both DPCs and inter-strand crosslinks [ICLs]) using the classical treatment regimen. In contrast, SPRTN-deficient worms were resistant to DNA damage induced by UV light or IR, consistent with a specific function in DPC repair (Figure 1B). Notably, SPRTN-deficient worms tolerated Top1 adducts induced by CPT (Figure S1B).

The Fanconi anemia (FA) pathway provides resistance toward crosslinking compounds by coordinating replication-coupled repair of ICLs (Kottemann and Smogorzewska, 2013). Moreover, cells lacking the FA pathway are hypersensitive to reactive aldehydes, such as formaldehyde or acetaldehyde (Langevin et al., 2011; Rosado et al., 2011). However, a deficiency in the FA protein FANCD2 (*fcd-2*) in worms did not result in increased formaldehyde sensitivity of synchronized L1 larvae, even if SPRTN (*dvc-1*) also was deleted (Figure 1C). Given that the FA pathway is a replication-coupled repair pathway, this result was perhaps not surprising, because cells of synchronized L1 larvae are arrested at the G1/S transition and are devoid of detectable DNA replication (Baugh, 2013). In agreement with previous studies, FANCD2-deficient worms were sensitive to cisplatin when exposed chronically (Figures 1D and S1C). Notably, worms lacking SPRTN were significantly more sensitive to cisplatin than *fcd-2* mutant animals, with the double mutant showing an additive, but not synergistic, phenotype. This indicates that FANCD2 and SPRTN operate in genetically distinct DNA repair pathways, which cannot compensate for each other. In turn, this suggests that they repair two different types of damage caused by cisplatin, which are presumably ICLs and DPCs, respectively.

Nucleotide excision repair (NER) has been implicated in replication-independent repair of formaldehyde-induced DPCs in several organisms. However, we did not observe increased formaldehyde sensitivity in worms lacking the crucial NER factor XPA (*xpa-1*), even in the absence of SPRTN (Figures 1E, S1D, and S1E). In yeast, homologous recombination acts parallel to

(F) Loss of SPRTN (*dvc-1*) results in viability defects in worms lacking the BLM helicase (*him-6*). Data were obtained from at least 16 animals per indicated genotype. Whiskers indicate tenth to 90th percentiles. Statistical significance was tested using an unpaired t test.
(G) The BLM helicase (Him-6) is not involved in providing formaldehyde resistance in synchronized L1 larvae. Error bars indicate SEM of two independent experiments.
(H) BLM (Him-6) provides resistance to cisplatin exposure by a mechanism parallel to DPC repair by SPRTN. Cisplatin sensitivity was assessed by measuring embryonic survival of progeny after exposure of adult animals. Error bars indicate SEM of two independent experiments.
See also Figure S1.

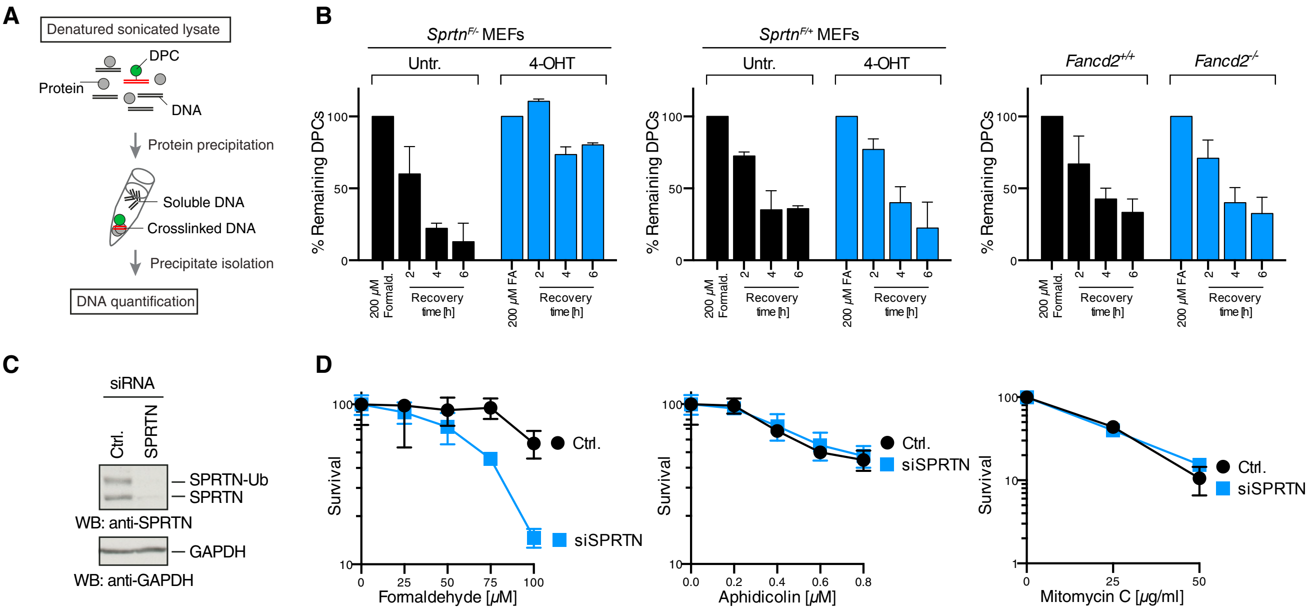

Figure 2. SPRTN-Deficient Mammalian Cells Fail to Repair DPCs and Are Hypersensitive toward DPC-Inducing Agents

(A) Schematic representation of the KCl/SDS precipitation assay used to measure DPC repair. Cells are lysed in denaturing conditions (1% SDS), followed by sonication and precipitation of cellular protein by the addition of KCl. Crosslinked DNA co-precipitates with the protein, whereas free DNA remains in the supernatant. The precipitate is washed several times before quantification of soluble and crosslinked DNA.

(B) SPRTN-deficient MEFs fail to repair formaldehyde-induced DPCs. *Sprtn*$^{F/-}$, *Sprtn*$^{F/+}$ (untreated or treated with 4-hydroxy tamoxifen [4-OHT] for 48 hr), *Fancd2*$^{+/+}$, and *Fancd2*$^{-/-}$ MEFs were treated with 200 µM formaldehyde (FA) for 1 hr to induce DPCs and lysed directly or allowed to repair. DPCs were measured as the ratio of crosslinked DNA compared to total DNA. Error bars indicate SEM of two independent experiments.

(C and D) Knockdown of SPRTN results in formaldehyde sensitivity in human cells. Relative cell numbers were determined 6 days after U2OS cells transfected with SPRTN or control siRNA were treated with the indicated doses of formaldehyde, aphidicolin, or mytomicin C. Error bars represent SD of two to four replicates.

See also Figure S2.

DPC proteolysis by Wss1, and loss of the HR factor BLM (*him-6* in worms) is synthetically lethal when combined with a Wss1 knockout (Mullen et al., 2011). Consistently, worms lacking both SPRTN and BLM (*dvc-1*; *him-6*) showed severe viability defects (Figure 1F). Worms lacking HIM-6/BLM were hypersensitive to cisplatin treatment, but not to acute formaldehyde exposure, in arrested L1 larvae, which implies that BLM acted exclusively in dividing cells (Figures 1G and 1H). The *dvc-1*; *him-6* double-mutant worms exhibited a synthetic effect, as they were unable to tolerate even very low doses of cisplatin. Taken together, our data reveal that SPRTN provides resistance toward DPC-inducing agents in worms by a mechanism acting parallel to BLM and independent of the FA pathway.

SPRTN-Deficient Mammalian Cells Fail to Repair Formaldehyde-Induced DPCs

The particular sensitivities of SPRTN-deficient worms suggest a specific function for SPRTN in the repair of DPCs. Hence, we tested this possibility directly by measuring DPC repair capacity in mammalian cells. To this end, we induced DPCs in immortalized conditional *Sprtn*$^{F/-}$ mouse embryonic fibroblasts (MEFs) by formaldehyde and followed DPC repair using a KCl/SDS precipitation assay (Figure 2A) (Zhitkovich and Costa, 1992). MEFs expressing functional SPRTN efficiently repaired formaldehyde-induced DPCs over time. Strikingly, however, inactivation of the remaining SPRTN allele in *Sprtn*$^{F/-}$ MEFs (but not in *Sprtn*$^{F/+}$ cells) resulted in an almost complete failure to repair DPCs (Figure 2B). In contrast, no DPC repair defect could be observed in MEFs lacking *Fancd2*.

Next we asked whether the failure to repair DPCs translates to hypersensitivity toward DPC-inducing agents in mammalian cells. Because a complete knockout results in lethality, we utilized knockdown of SPRTN by small interfering RNA (siRNA) in U2OS cells, which indeed resulted in sensitivity toward formaldehyde, but not toward ICL induction by mitomycin C or general replication inhibition by aphidicolin (Figures 2C and 2D).

A DNA Switch Controls SPRTN's Protease Activity

Our finding that SPRTN provides resistance to DPCs and is required for DPC repair suggests that it is indeed the elusive protease required for DPC processing in metazoans. To formally test this possibility in vitro, we purified human SPRTN (N-terminally GST tagged, C-terminally Strep tagged) (Figure 3A) from insect cells, and we assessed it for proteolytic activity toward DNA-associated proteins. In isolation, SPRTN exhibited no detectable proteolytic activity. However, the addition of DNA induced endoproteolytic autocleavage (Figure 3B), which also was observed with the yeast DPC-processing enzyme Wss1 (Stingele et al., 2014). Autoprocessing of SPRTN was induced with different types of single-stranded DNA (ssDNA) and

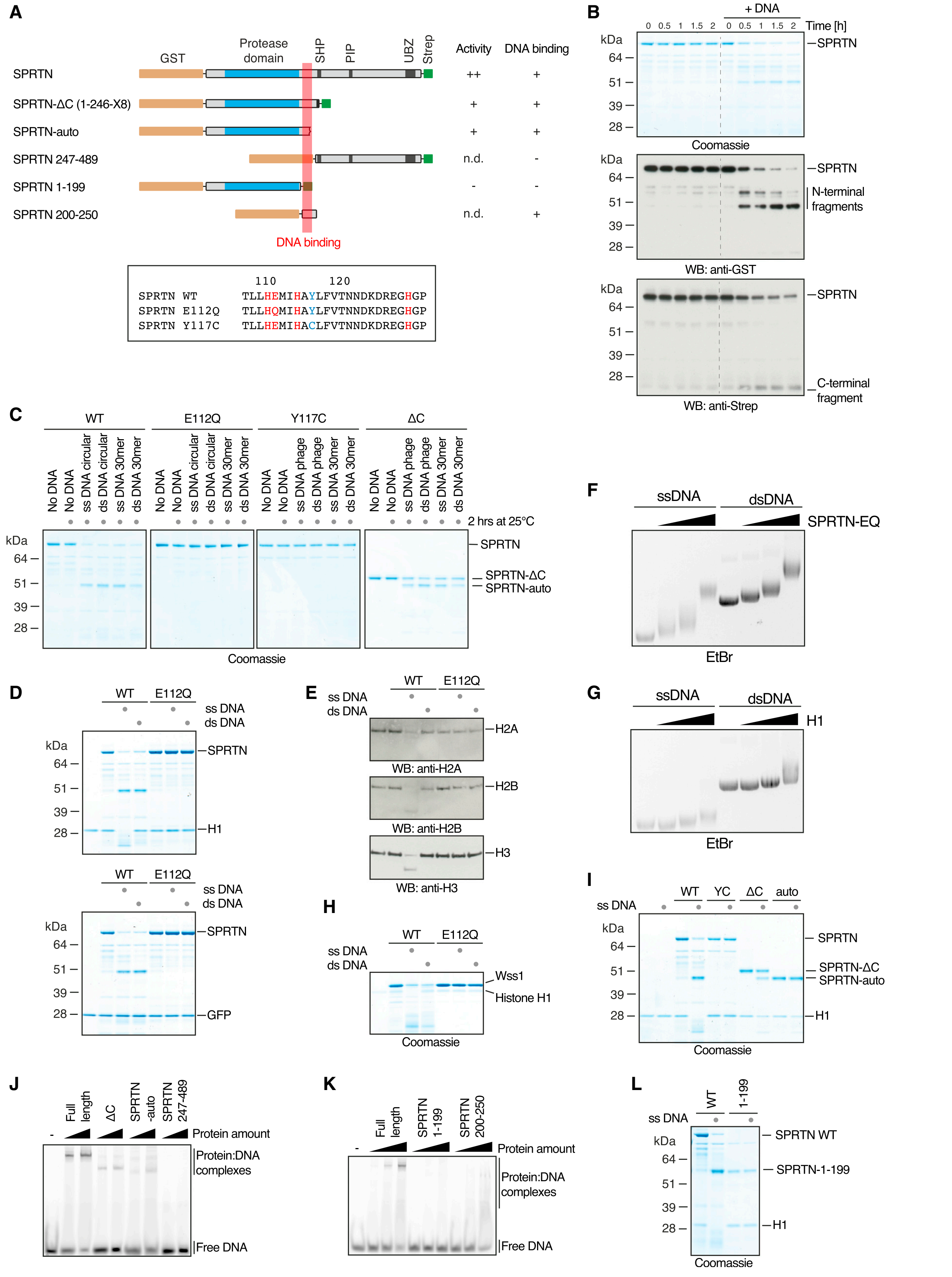

(legend on next page)

double-stranded DNA (dsDNA), but it was not observed if the active site glutamate residue was mutated to glutamine (E112Q, SPRTN-EQ) (Figures 3C and S2A). In agreement with SPRTN being a metalloprotease, the chelating compound 1,10-phenanthroline (OPA) inhibited autocleavage (Figure S2B).

We next assessed if the DNA-induced proteolytic activity of SPRTN is capable of cleaving DNA-associated proteins irrespective of identity. Indeed, SPRTN efficiently digested the DNA-binding proteins histone H1, H2A, H2B, H3, and Hmg1 in the presence of DNA, but it had no measurable activity toward non-DNA-binding proteins, such as GFP or BSA (Figures 3D, 3E, and S2C–S2G). Strikingly, and in contrast to autocleavage, substrates were digested only in the presence of ssDNA, but not dsDNA. Importantly, both SPRTN and its substrate (histone H1) bound very similarly to the ss and ds phage DNA used for activation (Figures 3F and 3G). Thus, our results indicate that SPRTN's protease activity is controlled by a DNA-specific switch, which allows the enzyme to operate in two modes: autocleavage only (dsDNA) or substrate and autocleavage (ssDNA). Intriguingly, cleavage of histone H1 by yeast Wss1 displayed the same DNA specificity, suggesting that the DNA switch is a universal feature of this protease family (Figure 3H).

The premature aging and cancer predisposition observed in RJALS patients are caused by mutations of the *SPRTN* gene, resulting in a C-terminally truncated protein (ΔC, amino acid [aa] 1–246 of SPRTN followed by eight amino acids [X8] caused by a frameshift) or a tyrosine-to-cysteine substitution (Y117C, SPRTN-YC) in close proximity to the active site (Figure 3A) (Lessel et al., 2014). Two affected patients were compound heterozygous for SPRTN-ΔC and SPRTN-Y117C, whereas the other reported patient possessed two alleles of the SPRTN-ΔC. To determine how these alterations affect the activity of SPRTN, we tested recombinantly expressed disease variants for DNA-dependent protease activity. Remarkably, SPRTN-Y117C was defective for DNA-dependent autocleavage as well as ssDNA-dependent substrate digestion (Figures 3C and 3I). In contrast, SPRTN-ΔC retained autocleavage activity, producing the same distinct N-terminal fragment as the WT enzyme, which we designate here as SPRTN-auto (Figures 3A and 3C). SPRTN-ΔC also digested substrates in an ssDNA-dependent manner; however, it showed reduced activity compared to WT enzyme (Figures 3I and S2H). To understand if the processing of SPRTN-ΔC into SPRTN-auto changes its activity, we purified the processed fragment (Figure S2I). However, SPRTN-auto was indistinguishable from SPRTN-ΔC with respect to substrate cleavage (Figure 3I).

The DNA-dependent activity of SPRTN-ΔC and SPRTN-auto suggests that these variants retain the ability to bind DNA. Indeed, both proteins shifted ssDNA in an electrophoretic mobility shift assay (EMSA), only to a slightly lesser extent than the full-length protein (Figure 3J). Conversely, the C-terminal part of SPRTN (SPRTN 247–489) did not show any DNA binding (Figure 3J). Next, we mapped the DNA-binding domain of SPRTN further to the region directly C-terminal to the protease domain. The aa 200–250 of SPRTN expressed as a GST-fusion shift DNA, while a SPRTN variant lacking this region (SPRTN 1–199) did not show DNA-binding activity (Figure 3K). Importantly, SPRTN lacking the DNA-binding domain (SPRTN 1–199) was devoid of detectable DNA-dependent protease activity, indicating that DNA binding is required for its activity (Figure 3L).

Crystal Structure of the Protease Domain of SPRTN's Fission Yeast Homolog

Our results suggest that the DNA-dependent activity of the SPRTN/Wss1 protease family is critical for its function in vivo. This activity is highly specific and promiscuous at the same time; only DNA-binding proteins are digested in a strictly DNA-dependent manner, yet irrespective of identity. To gain insights into how this feat is achieved, we sought to obtain structural information on this protease family. We focused on the protease domain, as the presumably highly dynamic C-terminal tail containing various protein-protein interaction motifs interferes with crystallization of full-length protein. We screened several

Figure 3. A DNA Switch Controls SPRTN's Protease Activity

(A) Schematic representation of recombinant GST-SPRTN-Strep variants (upper panel). Sequence of SPRTN's active site with catalytic residues in red and tyrosine-to-cysteine replacement found in RJALS patients in blue (lower panel) are shown.

(B) Autocatalytic cleavage of SPRTN is induced by DNA. SPRTN (180 nM, N-terminally GST tagged, C-terminally Strep tagged) was incubated in the absence or presence of circular ssDNA (ΦX174 virion, 10 nM).

(C) Autocatalytic cleavage of SPRTN is induced by various types of DNA. GST-SPRTN-Strep (WT, E112Q, or the disease variants Y117C and ΔC, 180 nM) was incubated in the presence of different types of DNA (phage DNA [10 nM], 30-mer oligonucleotides [1.8 μM]) for 2 hr at 25°C.

(D and E) SPRTN cleaves DNA-binding proteins in an ssDNA-dependent manner. GST-SPRTN-Strep (WT or the catalytically inactive E112Q variant, 480 nM) was incubated with the indicated substrates (360 nM) in the absence or presence of ss and ds phage DNA (10 nM) for 2 hr at 25°C.

(F and G) SPRTN and histone H1 bind similarly to ss and ds phage DNA. Proteins (SPRTN [0.45, 0.9, and 1.8 μM] and H1 [2, 3, and 4 μM]) were incubated with DNA (50 nM) and analyzed on 0.8% agarose gels.

(H) Wss1 cleaves histone H1 in an ssDNA-dependent manner. Wss1 (WT or the catalytically inactive E116Q variant, 800 nM) histone H1 (200 nM) were incubated with the indicated type of DNA (10 nM) for 2 hr at 30°C.

(I) SPRTN disease variants display defects in ssDNA-dependent substrate cleavage. GST-SPRTN-Strep (WT, Y117C, ΔC or auto, 480 nM) was incubated with histone H1 (360 nM) in the absence or presence of ss phage DNA (10 nM) for 2 hr at 25°C.

(J) C-terminally truncated SPRTN variants retain the ability to bind DNA. Indicated proteins (500 nM and 1 μM) were incubated with a fluorescently labeled ss oligonucleotide (250 nM) prior to gel electrophoresis in 6% PAGE gels.

(K) SPRTN's DNA-binding domain resides within aa 200–250. Indicated proteins (0.25, 0.5, and 1 μM) were incubated with a fluorescently labeled ss oligonucleotide (250 nM) prior to gel electrophoresis in 6% PAGE gels.

(L) SPRTN deficient for DNA binding is deficient for ssDNA-dependent substrate cleavage. Recombinant GST-SPRTN-Strep (WT or 1–199, 480 nM) was incubated with recombinant histone H1 (360 nM) in the absence or presence of ss phage DNA (10 nM) for 2 hr at 25°C

See also Figure S2.

Table 1. X-Ray Data Collection and Refinement Statistics of the Wss1 Structure from *S. pombe*

	Sp_Wss1b (17–151) Anomalous	Sp_Wss1b (17–151)	Sp_Wss1b (17–151) E112Q
Crystal Parameters			
Space group	$P2_12_12_1$	$P2_12_12_1$	$P2_1$
Cell constants	a = 40.1 Å	a = 40.3 Å	a = 41.2 Å
	b = 41.4 Å	b = 41.3 Å	b = 57.3 Å
	c = 68.3 Å	c = 68.5 Å	c = 50.6 Å
			β = 113.0
Wss1b/AU[a]	1	1	1
Data Collection			
Beam line	X06DA, SLS	X06DA, SLS	X06DA, SLS
Wavelength (Å)	1.4854	0.8	1.0
Resolution range (Å)[b]	30–1.8 (1.9–1.8)	30–1.0 (1.1–1.0)	30–1.75 (1.85–1.75)
Number of observations	75,642	545,122	72,610
Number of unique reflections[c]	15,941[d]	61,720[e]	20,977[e]
Completeness (%)[b]	95.2 (92.4)	98.7 (99.7)	95.3 (94.6)
R_{merge} (%)[b,f]	4.6 (23.6)	5.9 (39.8)	4.1 (43.1)
I/σ (I)[b]	18.7 (5.1)	21.0 (4.9)	14.1 (2.7)
Refinement (REFMAC5)			
Resolution range (Å)		15.0–1.0	15.0–1.75
Number of refl. working set		58,634	19,928
Number of refl. test set		3,086	1,049
Number of non-hydrogen		1,209	2,058
Number of of Ni^{2+}		1	2
Solvent/ions		225	145
R_{work}/R_{free} (%)[g]		0.143/0.168	0.173/0.195
RMSD bond (Å)/(°)[h]		0.009/1.4	0.005/1.0
Average B-factor ($Å^2$)		11.7	39.9
Ramachandran plot (%)[i]		99.1/0.9/0.0	97.7/2.3/0.0
PDB accession code		5JIG	5LN5

Refl., reflections.
[a]Asymmetric unit.
[b]The values in parentheses for resolution range, completeness, R_{merge}, and I/σ (I) correspond to the highest resolution shell.
[c]Data reduction was carried out with XDS and from a single crystal.
[d]Friedel pairs were treated as individual reflections.
[e]Friedel pairs were treated as identical reflections.
[f]$R_{merge}(I) = \Sigma_{hkl}\Sigma_j \mid I(hkl)_j - < I(hkl) > \mid / \Sigma_{hkl} \Sigma_j I(hkl)_j$, where $I(hkl)_j$ is the jth measurement of the intensity of reflection hkl and $< I(hkl) >$ is the average intensity.
[g]$R = \Sigma_{hkl} \mid |F_{obs}| - |F_{calc}| \mid / \Sigma_{hkl} |F_{obs}|$, where R_{free} is calculated without a sigma cutoff for a randomly chosen 5% of reflections, which were not used for structure refinement, and R_{work} is calculated for the remaining reflections.
[h]Deviations from ideal bond lengths/angles (RMSD, root-mean-square deviation).
[i]Number of residues in favored region/allowed region/outlier region.

constructs of different SPRTN/Wss1 representatives from various organisms for the expression in *E. coli.* While most proteins were insoluble, we were able to purify the protease domain of Wss1b, one of the two SPRTN homologs in *Schizosaccharomyces pombe*. Wss1b was crystallized and its structure determined by single-wavelength anomalous dispersion (SAD) at a resolution of 1.0 Å and R_{free} = 16.8% (PDB: 5JIG) (Table 1). An X-ray fluorescence spectrum of crystals obtained at the synchrotron revealed the presence of nickel as the only heavy-metal atom. An anomalous dataset at the Ni^{2+} edge (z = 1.4854) confirmed that this ion occupies the active site of the enzyme, where it replaced the catalytic zinc presumably during the Ni^{2+}-affinity chromatography.

The overall architecture depicted a compact protease domain consisting of four tightly packed α helices and a four-stranded antiparallel β sheet (Figure 4A). The catalytic center was formed by three histidines, as well as two water molecules and one oxygen molecule, that jointly coordinated the active site metal ion by forming a distorted octahedron (Figure 4B). The glutamate residue E112 polarized a water molecule for the nucleophilic attack of the substrate; in agreement, its mutation to glutamine resulted in a catalytically inactive enzyme (Figure 3). Moreover,

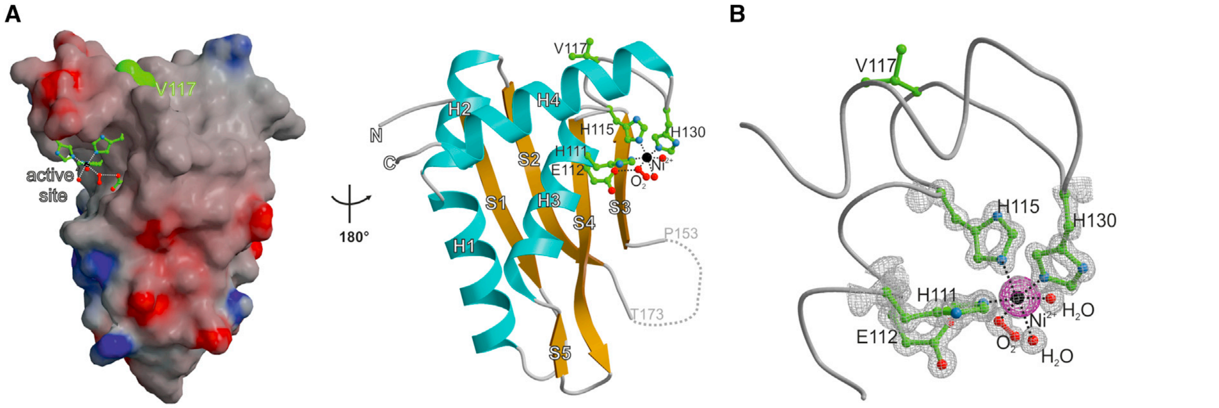

Figure 4. Crystal Structure of the Protease Domain of SPRTN's Fission Yeast Homolog

(A) Structure of the protease domain of *S. pombe* Wss1b (PDB: 5JIG) in a surface (left) or cartoon (right) representation. Active site residues are displayed as sticks. Position 117 mutated in RJALS to a cysteine is highlighted in green. Numbering of residues corresponds to the human sequence.

(B) Close-up view of the active site showing the octahedral coordination of Ni^{2+} by His111, His115, His130, as well as one oxygen and two water molecules. The $2F_O$-F_C electron density map is contoured to 1σ, whereas the anomalous density (magenta) for Ni^{2+} is contoured to 10σ. Most likely the catalytic zinc atom has been replaced during the Ni^{2+}-affinity chromatography step.

See also Figure S3.

we solved the structure of the EQ mutant (PDB: 5LN5) (Figure S3A), which confirmed that this mutation does not result in general structural alterations, thereby validating our biochemical analysis. Intriguingly, the catalytic center comprising the metal-binding motif was highly solvent exposed. This together with the absence of an obvious substrate-binding cleft or region could explain the promiscuity of SPRTN/Wss1 proteases with respect to substrate identity. A structure-based search for homologous topologies using the DALI-server revealed structures with *Z* scores < 9. All hits displayed a sequence identity of <10% and differed in at least one active site residue.

Intriguingly, the structure revealed that position 117 mutated in RJALS is in close proximity to the active site residues, but it does not seem to be involved in metal binding (Figure 4B). This position is only conserved in higher eukaryotes, suggesting that it acquired an important function only later in evolution (Figure S3B). Interestingly, this residue is solvent exposed and is followed in metazoans by a conserved insertion. This lobe is positioned next to the active site and might be required for stable substrate binding. The change to a cysteine residue at position 117 could result in a tilting of this lobe, thereby interfering with substrate binding.

DNA Binding Induces Conformational Changes in SPRTN

The exposition of the active site within the protease fold argues that the catalytic center might require structural shielding in the context of the full-length protein in order to prohibit unwanted proteolysis. In order to test if a conformational change is involved in the DNA-dependent activation of SPRTN, we probed the overall configuration of the protease by a limited proteolysis assay in the presence or absence of DNA. Strikingly, a distinctly different cleavage pattern could be observed when catalytically inactive SPRTN-EQ was digested by trypsin in the presence of DNA (Figures 5A and 5B). In the absence of DNA, digestion resulted in the formation of one major intermediate (fragment 1). In contrast, the production and/or stability of this fragment was dramatically reduced in the presence of DNA, with two distinct intermediates (fragments 2 and 3) being formed instead. Moreover, SPRTN generally was digested quicker in the presence of DNA, indicating a generally more open conformation. Ultimately, SPRTN was degraded entirely with only the GST tag remaining (fragment 4). Interestingly, the conformational change in the presence of DNA appeared to be more complete with ssDNA compared with dsDNA.

To further characterize the conformational change of SPRTN induced by DNA, we collected small angle X-ray scattering (SAXS) data on SPRTN-EQ in the absence or presence of ssDNA (15-mer) (Figures 5C, S4A, and S4B). The data indicated that the DNA-free protein is flexible, but that the addition of DNA increases flexibility significantly. The Rg of the DNA-bound SPRTN increased by 8–10 Å and the Dmax increased by 30 Å compared to DNA-free protein. Although we cannot formally exclude the possibility that the ssDNA sticks out, it is unlikely as (1) the ssDNA is small relative to the protein, (2) the ssDNA would likely be disordered and contribute less to the SAXS signal, and (3) a minimal ssDNA was used that shows an effect nevertheless. Porod analysis of the DNA-free protein indicated significant levels of flexibility, with a Porod Exponent of 2.7 (Rambo and Tainer, 2011). In the presence of DNA, the Porod Exponent changed to 2.5, indicating an increase in flexibility. This increase in flexibility also was observed in the Dimensionless Kratky, with the ssDNA-bound protein decreasing in peak height (Figure S4C) (Reyes et al., 2014). These results are consistent with the limited proteolysis data and with a model of an opening of the enzyme upon DNA binding.

To gain further insights into the regions of SPRTN involved in the conformational change, we performed hydrogen/deuterium (H/D) exchange mass spectrometry on SPRTN-EQ in the absence and presence of ssDNA (Figures 5D and S5D).

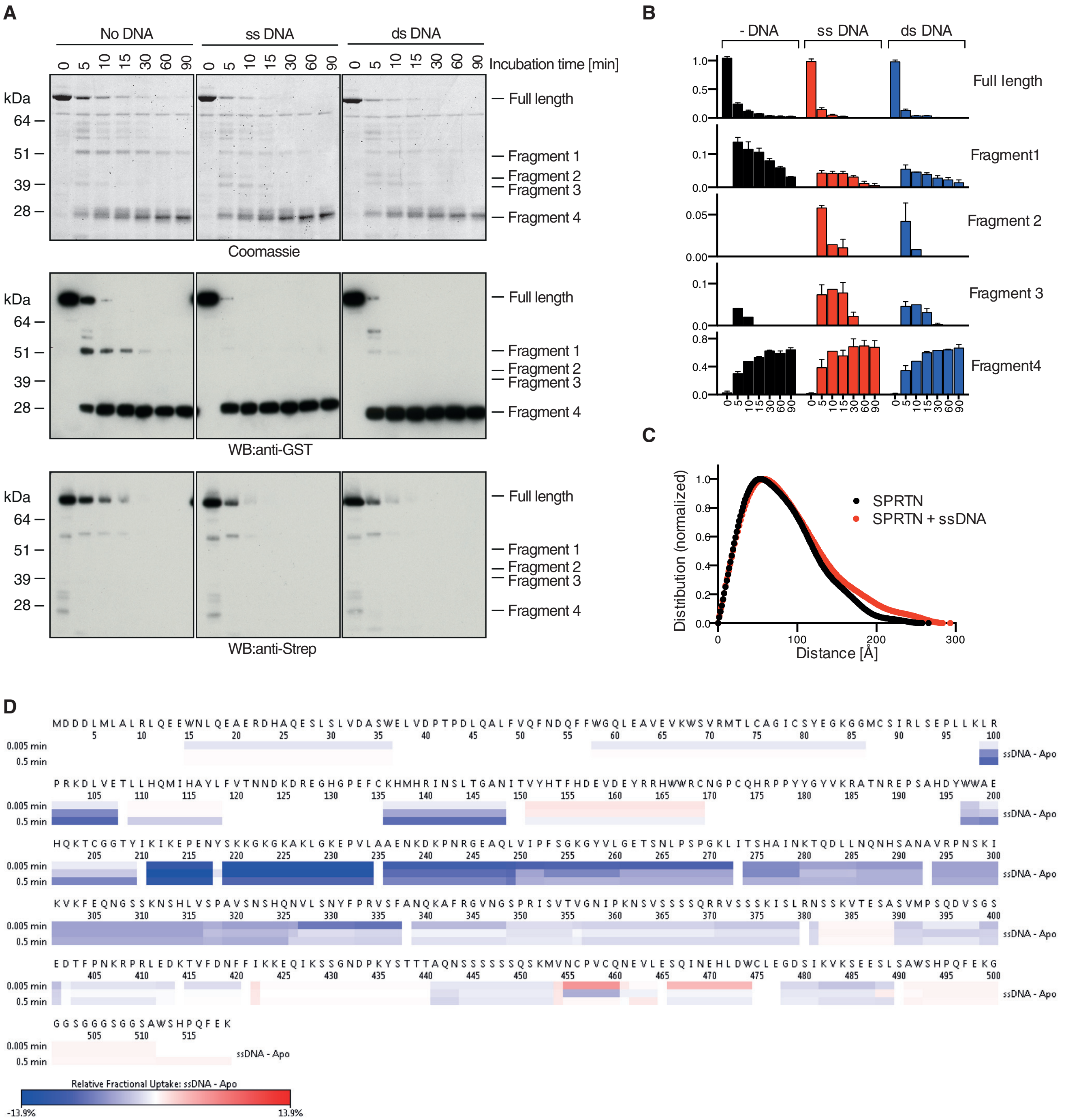

Figure 5. DNA Binding Induces a Conformational Change within SPRTN

(A) SPRTN undergoes a conformational change upon DNA binding. Catalytically inactive GST-SPRTN-Strep E112Q was subjected to limited proteolytic digestion by trypsin in the presence or absence of ssDNA or dsDNA.

(B) Quantification of specific proteolytic fragments observed in (A). Values represent mean ± SEM of three independent experiments.

(C) SAXS analysis indicates that ssDNA binding increases the flexibility of SPRTN. Electron pair distribution shows an increase in Rg and Dmax upon ssDNA (15-mer) binding.

(D) Heatmap showing H/D exchange mass spectrometry indicating differences in deuterium incorporation between SPRTN and SPRTN + ssDNA. Regions of increased protection are shown in blue and increased exposure in red. Deuterium labeling was carried out at three time points (0.3, 3, and 30 s) in triplicates. See also Figure S4.

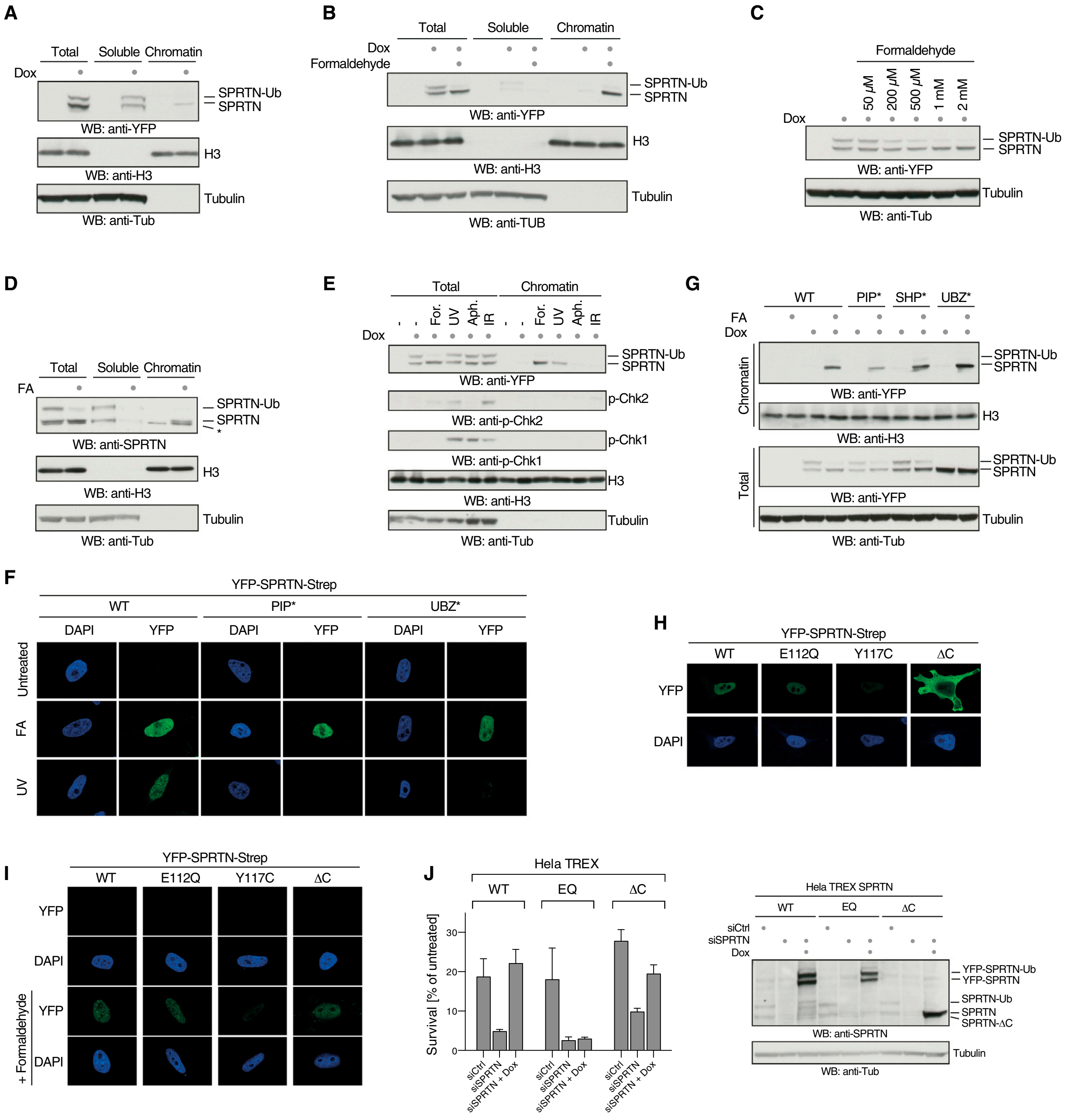

Figure 6. Chromatin Access of SPRTN Is Controlled by a DPC-Triggered Ubiquitin Switch

(A) Mono-ubiquitinated SPRTN is excluded from chromatin. Doxycycline-inducible YFP-SPRTN-Strep HeLa Flp-In TRex cells were either lysed directly in SDS-containing loading dye (total) or subjected to fractionation in soluble and chromatin components.

(B) Formaldehyde treatment induces deubiquitination of SPRTN coinciding with a complete relocalization to chromatin. Doxycycline-inducible YFP-SPRTN-Strep HeLa Flp-In TRex cells were treated with 1 mM formaldehyde (FA) for 2 hr.

(C) SPRTN is deubiquitinated upon formaldehyde exposure in a dose-dependent manner. Doxycycline-inducible YFP-SPRTN-Strep HeLa Flp-In TRex cells were treated for 2 hr with the indicated dose of formaldehyde.

(D) Endogenous SPRTN is deubiquitinated and relocalizes to chromatin formaldehyde exposure. U2OS cells were treated with 1 mM formaldehyde (FA) for 2 hr. Asterisk indicates an unspecific band.

(E) Deubiquitination of SPRTN is specifically triggered by DNA-protein crosslinks. Doxycycline-inducible YFP-SPRTN-Strep HeLa Flp-In TRex cells were treated with formaldehyde (FA, 1 mM, 2 hr), UVC light (UV, 20 J/m^2, 2 hr before lysis), aphidicolin (Aph, 1 μM, 2 hr), or IR (3 Gy, 2 hr before lysis).

(legend continued on next page)

Intriguingly, the region around the DNA-binding domain (residues 200–250) became strongly protected (i.e., less exposed to the solvent) in the presence of DNA, most likely due to direct DNA binding. In addition, the active site was less exposed, probably because it engaged with a second SPRTN molecule as a substrate. In contrast, the C-terminal part of the protein tended to be rather more solvent exposed in the presence of DNA. Collectively, these results suggest that DNA binding induces a subtle but significant conformational change that enables the active site to engage with substrates.

A Ubiquitin Switch Controls SPRTN's Access to Chromatin

DNA binding and the associated conformational change appear to be an essential step for SPRTN activation. Thus, we sought to understand how chromatin recruitment and DNA binding are controlled in vivo. SPRTN is present in cells in two forms, unmodified and mono-ubiquitinated (Mosbech et al., 2012), and a fraction of SPRTN is constitutively present on chromatin. We noticed that chromatin-associated YFP-tagged SPRTN consists only of the unmodified species, suggesting that the mono-ubiquitination regulates chromatin binding (Figure 6A). Strikingly, DPC induction by formaldehyde resulted in an almost complete deubiquitination of SPRTN coinciding with a relocalization of the entire SPRTN pool to chromatin (Figure 6B). That the mono-ubiquitinated form was indeed deubiquitinated was indicated by the fact that the amount of unmodified SPRTN increased in correspondence to the loss of modified SPRTN (Figure S5A). Moreover, the loss of modified SPRTN could not be explained by proteasomal degradation, because it still occurred in the presence of MG132 (Figure S5B). The deubiquitination of SPRTN upon DPC induction was induced in a dose- and time-dependent manner (Figures 6C and S5C). Importantly, endogenous SPRTN also was deubiquitinated upon formaldehyde exposure, which triggered its relocalization to chromatin (Figures 6D and S5D). Notably, purified mono-ubiquitinated and unmodified YFP-tagged SPRTN displayed very similar autocleavage kinetics, indicating that the modification did not influence SPRTN's activity (Figures S5E and S5F).

Using mass spectrometry, we identified four ubiquitination sites in SPRTN's C terminus (lysines 341, 376, 414, and 435), which were strongly reduced upon formaldehyde treatment and absent in an SPRTN-UBZ* mutant, which lacked mono-ubiquitination (Figure S5G) (Mosbech et al., 2012). However, an SPRTN variant with these lysines mutated to arginines (SPRTN-4KR) was still mono-ubiquitinated (Figure S5H). Mutation of six further lysines in the vicinity (361, 384, 407, 423, 424, and 427) failed to abolish mono-ubiquitination. We conclude that the modification can jump to alternative lysines, suggesting that the actual site of modification is not crucial for its function. The SPRTN-10KR was unstable and expressed at low levels, which precluded additional mutational efforts (Figure S5H).

Notably, deubiquitination appears to be specific for formaldehyde treatment, as other types of DNA damage, induced by UV, IR, or aphidicolin, did not result in strong deubiquitination of SPRTN (Figure 6E). Nonetheless, unmodified SPRTN was recruited to chromatin upon UV exposure, as has been published previously (Centore et al., 2012), but not by IR or aphidicolin (Figure 6E). Interestingly, SPRTN bears several protein-protein interaction motifs in its C-terminal tail, allowing it to associate with PCNA (PIP-box), the AAA-ATPase p97 (SHP-box), and ubiquitin (UBZ) (Figure 1A). UV-induced chromatin recruitment of SPRTN depended entirely on its PIP-box and on its UBZ domain (Figures 6F and S5I) (Centore et al., 2012). In contrast, recruitment upon formaldehyde treatment appeared to be mostly independent of PCNA binding and did not require its UBZ domain (Figures 6F and 6G). This is in line with the essential cellular function of SPRTN being independent of its C-terminal region. SPRTN variants lacking the interaction motifs for PCNA, p97, or ubiquitin binding in its C terminus rescue the growth defect of *Sprtn*$^{-/-}$ cells (Maskey et al., 2014). Moreover, patients lacking the entire C-terminal domain of SPRTN (SPRTN-ΔC) are viable, whereas a complete SPRTN knockout is lethal (Lessel et al., 2014). The SPRTN-ΔC variant displayed significantly higher expression levels and was mislocalized in cells (Figure 6H). Nonetheless, some SPRTN-ΔC could be recruited to chromatin upon formaldehyde treatment, consistent with its ability to bind DNA in vitro (Figures 6I and S5J). In agreement, SPRTN-ΔC could partially complement the formaldehyde sensitivity caused by the loss of SPRTN (Figures 6J–6L). Thus, despite lacking the protein-protein interaction motifs present in SPRTN's C-terminal part, SPRTN-ΔC retained partial functionality, perhaps explaining the viability of the patients.

Taken together, our data reveal that SPRTN is recruited to chromatin in the presence of DPCs, a process that is tightly linked to its deubiquitination and mechanistically distinct from its recruitment to UV damage. We propose that rapid

(F) SPRTN is differentially recruited to chromatin depending on the type of DNA damage. Doxycycline-induced YFP-SPRTN-Strep HeLa Flp-In TRex cells were treated with formaldehyde (FA, 0.5 mM) or UV (20 J/m^2) and subjected to pre-extraction, prior to fixation and immunofluorescence.

(G) SPRTN deubiquitination and chromatin recruitment upon DPC induction is independent of binding to PCNA, p97, or ubiquitin. Doxycycline-inducible YFP-SPRTN-Strep HeLa Flp-In TRex cells expressing the indicated SPRTN variants were treated with 1 mM formaldehyde (FA) for 2 hr.

(H) SPRTN-ΔC displays an aberrant subcellular localization. Doxycycline-induced YFP-SPRTN-Strep HeLa Flp-In TRex cells were analyzed using immunofluorescence.

(I) SPRTN-ΔC is recruited to chromatin upon the induction of DPCs. Doxycycline-induced YFP-SPRTN-Strep HeLa Flp-In TRex cells were treated with formaldehyde (FA, 0.5 mM) and subjected to pre-extraction, prior to fixation and immunofluorescence.

(J) SPRTN-ΔC complements the formaldehyde sensitivity of SPRTN-deficient cells only partially. HeLa Flp-In TRex cells bearing the indicated doxycycline-inducible YFP-SPRTN-Strep alleles were transfected with siRNA against endogenous SPRTN and incubated in the absence or presence of doxycycline for 48 hr. Cells were then treated for 48 hr with 100 μM formaldehyde, and cell numbers were determined after an additional 4-day incubation. Values indicate cell numbers relative to untreated cells. Error bars represent SD of two to four replicates. Knockdown and doxycycline induction were confirmed by western blotting. Please note that autocleavage bands appear at similar positions as endogenous SPRTN in cells expressing WT YFP-SPRTN.

See also Figure S5.

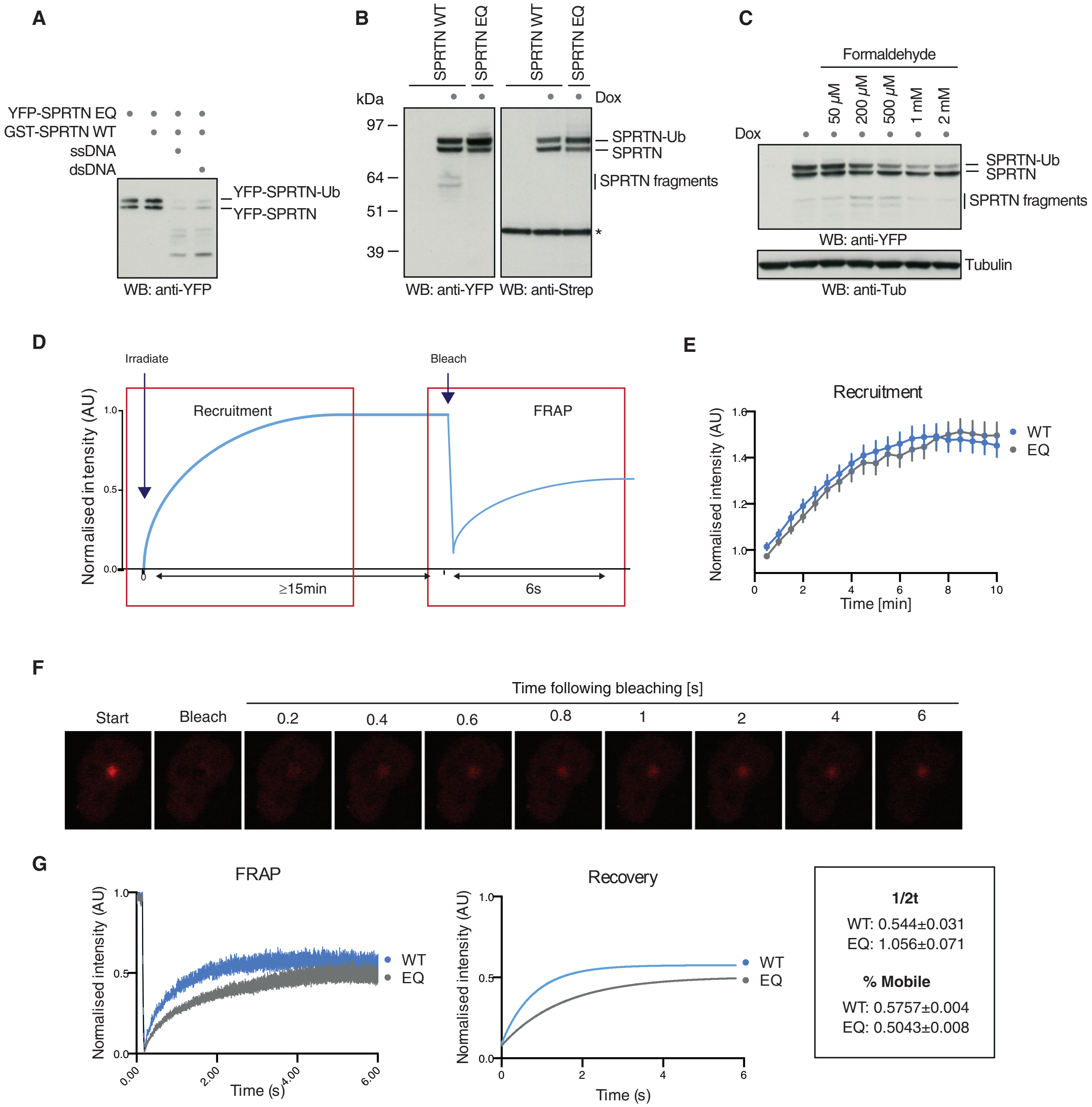

Figure 7. Autocleavage Controls SPRTN Dynamics at Sites of DNA Damage

(A) SPRTN autocleavage occurs in *trans*. Recombinant GST-SPRTN-Strep WT and catalytically inactive YFP-E112Q-Strep were incubated in the absence or presence of ss and ds phage DNA (10 nM) for 2 hr at 25°C.

(B) SPRTN autocleavage occurs in cells. Doxycycline-inducible YFP-SPRTN-Strep HeLa Flp-In TRex cells expressing the indicated SPRTN variants were lysed and subjected to SDS-PAGE followed by western blotting against the N-terminal YFP and the C-terminal Strep tag. The asterisk indicates an unspecific band serving as loading control.

(C) SPRTN autocleavage is triggered by formaldehyde. Doxycycline-inducible YFP-SPRTN-Strep HeLa Flp-In TRex cells were treated with the indicated dose of formaldehyde for 2 hr.

(D) Schematic representation shows laser microirradiation and fluorescence recovery after photobleaching (FRAP) experiments.

(E) Recruitment of YFP-SPRTN-Strep (WT or EQ) in HeLa Flp-In TRex cells after laser microirradiation. Data are from ≥20 cells ± SEM normalized to pre-irradiation fluorescence.

(legend continued on next page)

deubiquitination upon DPC induction ensures that SPRTN is only localized to chromatin when its proteolytic activity is required.

Autocatalytic Cleavage Negatively Regulates SPRTN at Damage Sites

SPRTN's recruitment to chromatin appears to be highly regulated, suggesting the existence of a mechanism that ensures that SPRTN is eventually turned off. Intrigued by the different properties of autocleavage and substrate cleavage by SPRTN in vitro (Figure 3), we speculated that this might have a regulatory function in vivo. First we asked whether autocleavage occurs in *trans* or in *cis.* GST-tagged WT SPRTN was able to process catalytically inactive YFP-tagged SPRTN-EQ in the presence of DNA, showing that autocleavage occurs in *trans* (Figure 7A). Notably, SPRTN-EQ was processed in the presence of ssDNA and dsDNA, indicating that this is a true autocleavage event. Next we tested whether autocleavage occurs in cells. Indeed, N-terminal fragments could be observed in cells expressing YFP-SPRTN-Strep. These fragments were absent in cells expressing a catalytically inactive SPRTN variant, suggesting that they are produced by autocatalytic cleavage (Figure 7B). Interestingly, the levels of autoproteolytic fragments increased when cells were exposed to formaldehyde, implying that autocleavage is functionally linked to DPC repair by SPRTN (Figure 7C).

To determine if autocleavage has a regulatory role, we conducted live-cell experiments to study the dynamics of SPRTN recruitment to sites of laser-inflicted DNA damage (Figure 7D), to which it previously has been shown to be recruited (Davis et al., 2012). Importantly, recruitment was independent of PCNA binding, as it was for formaldehyde-induced damage (Figure S6A). SPRTN-WT and SPRTN-EQ were recruited with identical kinetics to the site of damage (Figures 7E and S6B). However, the significantly slower recovery after bleaching revealed that SPRTN-EQ remained much more stably associated with the damage site once recruited (Figures 7F and 7G). We conclude that autocleavage plays a crucial role in removing SPRTN from sites of DNA damage, which likely restricts unwanted proteolysis on chromatin.

DISCUSSION

Its remarkable DNA-dependent proteolytic activity renders SPRTN ideal for efficient processing of crosslinked proteins, irrespective of their identity. Needless to say, this is a very toxic activity with the potential to degrade any chromatin protein if not properly controlled. Our structural data further highlighted the need to restrain the protease activity of the SPRTN/Wss1 family since the catalytic center is solvent exposed and bears few signs of specificity-generating features (Figure 4).

We discovered several molecular mechanisms, switches, that restrain SPRTN's activity and, consequently, control DPC repair in metazoans. The ubiquitin switch appears to be the most upstream control mechanism of SPRTN activity. Mono-ubiquitinated SPRTN is excluded from chromatin; however, the induction of DPCs by formaldehyde triggers its deubiquitination, thus allowing chromatin relocalization. Hence, this switch regulates SPRTN by adjusting the level of chromatin-accessible SPRTN in correspondence to the amount of DPC damage. Unmodified SPRTN is able to access chromatin and bind DNA, which triggers another regulatory mechanism, the DNA switch. Intriguingly, the DNA switch can be activated in two distinct modes depending on the type of DNA to which SPRTN is bound. The dsDNA binding renders the protease active, but only with respect to autocleavage. In contrast, ssDNA binding also induces substrate cleavage. Our data indicate two means by which the DNA switch regulates SPRTN. First, DNA binding causes SPRTN to adopt a more open conformation. Notably, the structural change induced by ssDNA compared to dsDNA is more stable, which may allow SPRTN to efficiently bind and, thus, process substrate proteins. Second, DNA serves as a scaffold that brings the enzyme in close proximity to its substrate, thereby increasing the mean residence time. This allows completion of the proteolysis reaction, despite SPRTN's low affinity toward substrates, which is underlined by the enzymes inability to cleave non-DNA-associated proteins, even if activated by DNA. Thus, the low specificity and the concomitant low affinity of the protease serve two purposes: enabling SPRTN to process a variety of substrates but also restraining unwanted proteolysis.

Finally, we identified an additional safeguarding mechanism that negatively regulates SPRTN's activity, the autocatalytic off switch. Induction of DPCs by formaldehyde in cells not only results in SPRTN activation, as inferred from its deubiquitination and relocalization to chromatin, but also in increased autocleavage, which is crucial for the eventual release of the enzyme (Figure 7). Intriguingly, autocleavage is most apparent with intermediate doses of formaldehyde compared to high doses. In contrary, deubiquitination is induced with a linear dose response. This perhaps reflects a balance between turning the SPRTN pathway on and off. High levels of DPCs require the entire pool of SPRTN for repair and, thus, little autocleavage is observed. Intermediate levels of formaldehyde activate the pathway, but they also result in autocleavage adjusting the amount of active enzyme corresponding to the amount of DPC damage. Furthermore, dsDNA binding induces exclusively autocleavage, thus efficiently insulating undamaged chromatin to unwanted cleavage by SPRTN. Conversely, this suggests that ssDNA needs to be present at sites of DPCs in order to allow proteolysis.

Intriguingly, the two scenarios inducing replication-coupled DPC proteolysis involve ssDNA being present in close vicinity. DPCs located on the leading strand stall progression of the replicative helicase, thereby triggering DPC proteolysis (Duxin et al., 2014). Conversely, lagging strand DPCs can be bypassed by the helicase but stall DNA synthesis by the DNA polymerase,

(F) Representative images of HeLa Flp-In TRex cells expressing WT YFP-SPRTN-Strep from FRAP time course at indicated time following bleaching. Bleaching was achieved with 0.1-s pulse of 405-nM laser (scale bar, 10 μm).

(G) FRAP from HeLa Flp-In TRex cells expressing YFP-SPRTN-Strep (WT or EQ) data are from ≥15 cells ± SEM normalized to pre-bleach fluorescence (left panel). Fitted exponential fluorescence recovery of FRAP data is shown (right panel).

which again triggers DPC proteolysis. An important issue requiring further attention is how this stalling is signaled, resulting in the recruitment and activation of DPC proteases. Classical checkpoint signaling does not seem to be strongly involved, as formaldehyde does not induce Chk1 activation and only results in very low Chk2 phosphorylation (Figure 5). Chk2 activation is presumably triggered by double-strand breaks resulting from cleavage of DPC-stalled forks. In agreement, SPRTN deficiency in mouse cells is accompanied by Chk2, but not Chk1, activation (Maskey et al., 2014). Thus, there has to be a different signaling mechanism in place to induce the deubiquitination of SPRTN. Indeed, a specific ubiquitination signal seems to be required, as a dominant-negative ubiquitin mutant inhibits DPC repair in *Xenopus* (Duxin et al., 2014). Determining the nature of this signal together with the enzymes (E3 ligase and deubiquitinating enzyme) regulating SPRTN mono-ubiquitination will be paramount to understanding the complex signaling mechanisms orchestrating DPC repair.

Our data strongly suggest that DPC repair is the main function of SPRTN. In turn it seems likely that faulty DPC repair is the molecular defect underlying RJALS. The two reported patient alleles have differing effects on SPRTN's activity. SPRTN-ΔC retains residual activity in vitro and in vivo, probably explaining the viability of the patients. Additionally, the loss of the C terminus seems to interfere with proper subcellular localization and regulated chromatin recruitment. The second disease variant SPRTN-Y117C is catalytically inactive in vitro and appears to be less stable, as indicated by its low expression levels in human cells. As a consequence, the patients develop early-onset hepatocellular carcinoma and severe premature aging (Lessel et al., 2014). Intriguingly, the liver is the major detoxifying organ where the bulk of metabolic processes producing reactive aldehydes occur. Thus, it seems likely that cells in the liver face significantly more DPCs compared to other tissues. Interestingly, some of the pathologies observed in RJALS are difficult to explain by a replicative role of SPRTN. The vast majority of liver cells are in a quiescent state, suggesting that few DPCs will challenge cells during replication. Moreover, premature cataract has been observed in RJALS and in a hypomorphic SPRTN mouse model, which is a general sign of failure to maintain postmitotic tissue homeostasis (Maskey et al., 2014; Ruijs et al., 2003). A replication-independent function of SPRTN also is indicated by results in flies, where SPRTN is recruited to chromatin independently of replication (Delabaere et al., 2014). Consistently, we found that arrested L1 worm larvae, in which no replication is occurring, are extremely sensitive to acute formaldehyde exposure if they lack SPRTN. It seems plausible that cells may not risk repairing DPCs exclusively in S-phase, where a failure to complete repair has dramatic consequences. However, further work will be required to elucidate the replication-independent function of SPRTN.

The fact that SPRTN is essential in mammals suggests that cells are challenged with significant levels of spontaneous DPCs at any given time. In contrast, ICLs seem to occur less frequently, as components of the FA pathway are generally dispensable for viability. Similarly, DPC repair seems to be more important than ICL repair for providing tolerance to the crosslinking compound cisplatin (Figure 1D), indicating that DPCs contribute significantly to its cytotoxic activity. Notably, cisplatin derivatives (carboplatin and oxaliplatin) are widely used to treat ovarian and colon cancer. Hence, interfering with DPC repair by inhibiting SPRTN may represent a potential therapeutic opportunity that could be exploited to sensitize quickly dividing cancer cells to chemotherapy. At any rate, the emerging data on DPC repair by the SPRTN/Wss1 DPC protease family highlight the importance of this DNA repair pathway for genome integrity and human health.

EXPERIMENTAL PROCEDURES

DNA-Dependent Autocleavage Assays

Reactions were performed at 25°C in 20 μl containing 6 μl SPRTN (600 nM in 50 mM HEPES [pH 7.5], 250 mM NaCl, and 10% glycerol), 2 μl DNA (concentrations indicated in figure legends, in Tris-EDTA (TE) or water), and 12 μl H_2O. Several types of DNA were used for activation: circular ssDNA (ΦX174 virion, New England Biolabs), circular dsDNA (ΦX174 RF I, New England Biolabs), and 30-bp ss and ds oligonucleotides (5′- TAGCAAGGCACTGGTAGAATT CGGCAGCGT-3′). Reactions were stopped by the addition of 4× lithium dodecyl sulfate (LDS) sample buffer (Thermo Fisher Scientific) supplemented with β-mercaptoethanol and boiling at 95°C for 5 min, resolved on 4%–12% Bis-Tris gradient gels, and stained with InstantBlue.

DNA-Dependent Cleavage of DNA-Binding Proteins

Reactions were performed at 25°C in 20 μl containing 4 μl GST-SPRTN-Strep WT or variants (2.4 μM in 50 mM HEPES [pH 7.5], 250 mM NaCl, and 10% glycerol), 2 μl substrate (3.6 μM in 50 mM HEPES [pH 7.5], 250 mM NaCl, and 10% glycerol), 2 μl DNA (100 nM in TE), and 12 μl H_2O. Either circular ssDNA (ΦX174 virion, New England Biolabs) or circular dsDNA (ΦX174 RF I, New England Biolabs) was used for activation. Reactions were stopped by the addition of 4× LDS sample buffer (Thermo Fisher Scientific) supplemented with β-mercaptoethanol and boiling at 95°C for 5 min, resolved on 4%–12% Bis-Tris gradient gels, and stained with InstantBlue.

ACCESSION NUMBERS

The accession numbers for the structural data reported in this paper are PDB: 5JIG (Wss1 WT) and 5LN5 (Wss1 EQ).

SUPPLEMENTAL INFORMATION

Supplemental Information includes Supplemental Experimental Procedures and six figures and can be found with this article online at http://dx.doi.org/10.1016/j.molcel.2016.09.031.

AUTHOR CONTRIBUTIONS

J.S. and S.J.B. conceived and supervised the study and wrote the paper. J.S., R.B., and G.H. performed experiments. F.A. and M.G. performed structural analysis. S.L.M. and J.M.S. performed H/D exchange mass spectrometry. S.E.T. and J.A.T. performed SAXS analysis. G.S. generated reagents. A.B. and S.K. produced insect cell pellets.

ACKNOWLEDGMENTS

We thank Yuichi Machida for providing *Sprtn*$^{-}$ MEFs; John Rouse for anti-SPRTN polyclonal antibody; Bjoern Schumacher, Anton Gartner, and the *Caenorhabditis* Genetics Center for *C. elegans* strains; Vesela Encheva and Bram Snijders for mass spectrometry analysis; the staff of the macromolecular crystallography beamline X06SA (PXI) of the Paul Scherrer Institute, Swiss Light Source, for help with data collection; Stefan Jentsch for discussions and support; and members of the S.J.B. laboratory for comments and discussion throughout the project. SAXS data were collected at the SIBYLS beamline

12.3.1 at the Advanced Light Source (ALS) at Lawrence Berkeley National Laboratory, supported by the Department of Energy (DOE, IDAT program) and the National Cancer Institute (NCI, PO1CA92584). J.A.T. is supported by the Cancer Prevention and Research Institute of Texas and a Robert A. Welch Chemistry Chair. J.S. is supported by a European Molecular Biology Organization (EMBO) long-term fellowship (ALTF 470-2015), and G.S. is supported by an EMBO advanced fellowship (ALTF 1656-2014). This work was supported by the Francis Crick Institute, which receives its core funding from Cancer Research UK (FC0010048), the UK Medical Research Council (FC0010048), and the Wellcome Trust (FC0010048); a European Research Council (ERC) Advanced Investigator Grant (RecMitMei); and a Wellcome Trust Senior Investigator Grant.

Received: May 17, 2016
Revised: July 13, 2016
Accepted: September 22, 2016
Published: October 27, 2016

REFERENCES

Baker, D.J., Wuenschell, G., Xia, L., Termini, J., Bates, S.E., Riggs, A.D., and O'Connor, T.R. (2007). Nucleotide excision repair eliminates unique DNA-protein cross-links from mammalian cells. J. Biol. Chem. *282*, 22592–22604.

Barker, S., Weinfeld, M., and Murray, D. (2005). DNA-protein crosslinks: their induction, repair, and biological consequences. Mutat. Res. *589*, 111–135.

Baugh, L.R. (2013). To grow or not to grow: nutritional control of development during Caenorhabditis elegans L1 arrest. Genetics *194*, 539–555.

Centore, R.C., Yazinski, S.A., Tse, A., and Zou, L. (2012). Spartan/C1orf124, a reader of PCNA ubiquitylation and a regulator of UV-induced DNA damage response. Mol. Cell *46*, 625–635.

Davis, E.J., Lachaud, C., Appleton, P., Macartney, T.J., Näthke, I., and Rouse, J. (2012). DVC1 (C1orf124) recruits the p97 protein segregase to sites of DNA damage. Nat. Struct. Mol. Biol. *19*, 1093–1100.

de Graaf, B., Clore, A., and McCullough, A.K. (2009). Cellular pathways for DNA repair and damage tolerance of formaldehyde-induced DNA-protein crosslinks. DNA Repair (Amst.) *8*, 1207–1214.

Delabaere, L., Orsi, G.A., Sapey-Triomphe, L., Horard, B., Couble, P., and Loppin, B. (2014). The Spartan ortholog maternal haploid is required for paternal chromosome integrity in the Drosophila zygote. Curr. Biol. *24*, 2281–2287.

Duxin, J.P., Dewar, J.M., Yardimci, H., and Walter, J.C. (2014). Repair of a DNA-protein crosslink by replication-coupled proteolysis. Cell *159*, 346–357.

Friedberg, E.C., Elledge, S.J., Lehmann, A.R., Lindahl, T., and Muzi-Falconi, M. (2014). DNA Repair, Mutagenesis, and Other Responses to DNA Damage (New York: Cold Spring Harbor Laboratory Press).

Fu, Y.V., Yardimci, H., Long, D.T., Ho, T.V., Guainazzi, A., Bermudez, V.P., Hurwitz, J., van Oijen, A., Schärer, O.D., and Walter, J.C. (2011). Selective bypass of a lagging strand roadblock by the eukaryotic replicative DNA helicase. Cell *146*, 931–941.

Ghosal, G., Leung, J.W., Nair, B.C., Fong, K.W., and Chen, J. (2012). Proliferating cell nuclear antigen (PCNA)-binding protein C1orf124 is a regulator of translesion synthesis. J. Biol. Chem. *287*, 34225–34233.

Jackson, S.P., and Bartek, J. (2009). The DNA-damage response in human biology and disease. Nature *461*, 1071–1078.

Juhasz, S., Balogh, D., Hajdu, I., Burkovics, P., Villamil, M.A., Zhuang, Z., and Haracska, L. (2012). Characterization of human Spartan/C1orf124, an ubiquitin-PCNA interacting regulator of DNA damage tolerance. Nucleic Acids Res. *40*, 10795–10808.

Kottemann, M.C., and Smogorzewska, A. (2013). Fanconi anaemia and the repair of Watson and Crick DNA crosslinks. Nature *493*, 356–363.

Langevin, F., Crossan, G.P., Rosado, I.V., Arends, M.J., and Patel, K.J. (2011). Fancd2 counteracts the toxic effects of naturally produced aldehydes in mice. Nature *475*, 53–58.

Lessel, D., Vaz, B., Halder, S., Lockhart, P.J., Marinovic-Terzic, I., Lopez-Mosqueda, J., Philipp, M., Sim, J.C., Smith, K.R., Oehler, J., et al. (2014). Mutations in SPRTN cause early onset hepatocellular carcinoma, genomic instability and progeroid features. Nat. Genet. *46*, 1239–1244.

Lindahl, T. (1993). Instability and decay of the primary structure of DNA. Nature *362*, 709–715.

Machida, Y., Kim, M.S., and Machida, Y.J. (2012). Spartan/C1orf124 is important to prevent UV-induced mutagenesis. Cell Cycle *11*, 3395–3402.

Maskey, R.S., Kim, M.S., Baker, D.J., Childs, B., Malureanu, L.A., Jeganathan, K.B., Machida, Y., van Deursen, J.M., and Machida, Y.J. (2014). Spartan deficiency causes genomic instability and progeroid phenotypes. Nat. Commun. *5*, 5744.

Mosbech, A., Gibbs-Seymour, I., Kagias, K., Thorslund, T., Beli, P., Povlsen, L., Nielsen, S.V., Smedegaard, S., Sedgwick, G., Lukas, C., et al. (2012). DVC1 (C1orf124) is a DNA damage-targeting p97 adaptor that promotes ubiquitin-dependent responses to replication blocks. Nat. Struct. Mol. Biol. *19*, 1084–1092.

Mullen, J.R., Das, M., and Brill, S.J. (2011). Genetic evidence that polysumoylation bypasses the need for a SUMO-targeted Ub ligase. Genetics *187*, 73–87.

Nakano, T., Morishita, S., Katafuchi, A., Matsubara, M., Horikawa, Y., Terato, H., Salem, A.M., Izumi, S., Pack, S.P., Makino, K., and Ide, H. (2007). Nucleotide excision repair and homologous recombination systems commit differentially to the repair of DNA-protein crosslinks. Mol. Cell *28*, 147–158.

Nakano, T., Katafuchi, A., Matsubara, M., Terato, H., Tsuboi, T., Masuda, T., Tatsumoto, T., Pack, S.P., Makino, K., Croteau, D.L., et al. (2009). Homologous recombination but not nucleotide excision repair plays a pivotal role in tolerance of DNA-protein cross-links in mammalian cells. J. Biol. Chem. *284*, 27065–27076.

Nakano, T., Ouchi, R., Kawazoe, J., Pack, S.P., Makino, K., and Ide, H. (2012). T7 RNA polymerases backed up by covalently trapped proteins catalyze highly error prone transcription. J. Biol. Chem. *287*, 6562–6572.

Nakano, T., Miyamoto-Matsubara, M., Shoulkamy, M.I., Salem, A.M., Pack, S.P., Ishimi, Y., and Ide, H. (2013). Translocation and stability of replicative DNA helicases upon encountering DNA-protein cross-links. J. Biol. Chem. *288*, 4649–4658.

Pommier, Y. (2006). Topoisomerase I inhibitors: camptothecins and beyond. Nat. Rev. Cancer *6*, 789–802.

Pommier, Y., Huang, S.Y., Gao, R., Das, B.B., Murai, J., and Marchand, C. (2014). Tyrosyl-DNA-phosphodiesterases (TDP1 and TDP2). DNA Repair (Amst.) *19*, 114–129.

Rambo, R.P., and Tainer, J.A. (2011). Characterizing flexible and intrinsically unstructured biological macromolecules by SAS using the Porod-Debye law. Biopolymers *95*, 559–571.

Reyes, F.E., Schwartz, C.R., Tainer, J.A., and Rambo, R.P. (2014). Methods for using new conceptual tools and parameters to assess RNA structure by small-angle X-ray scattering. Methods Enzymol. *549*, 235–263.

Rosado, I.V., Langevin, F., Crossan, G.P., Takata, M., and Patel, K.J. (2011). Formaldehyde catabolism is essential in cells deficient for the Fanconi anemia DNA-repair pathway. Nat. Struct. Mol. Biol. *18*, 1432–1434.

Ruijs, M.W.G., van Andel, R.N.J., Oshima, J., Madan, K., Nieuwint, A.W.M., and Aalfs, C.M. (2003). Atypical progeroid syndrome: an unknown helicase gene defect? Am. J. Med. Genet. A. *116A*, 295–299.

Sczepanski, J.T., Wong, R.S., McKnight, J.N., Bowman, G.D., and Greenberg, M.M. (2010). Rapid DNA-protein cross-linking and strand scission by an abasic site in a nucleosome core particle. Proc. Natl. Acad. Sci. USA *107*, 22475–22480.

Shi, Y., Lan, F., Matson, C., Mulligan, P., Whetstine, J.R., Cole, P.A., Casero, R.A., and Shi, Y. (2004). Histone demethylation mediated by the nuclear amine oxidase homolog LSD1. Cell *119*, 941–953.

Stingele, J., and Jentsch, S. (2015). DNA-protein crosslink repair. Nat. Rev. Mol. Cell Biol. *16*, 455–460.

Stingele, J., Schwarz, M.S., Bloemeke, N., Wolf, P.G., and Jentsch, S. (2014). A DNA-dependent protease involved in DNA-protein crosslink repair. Cell *158*, 327–338.

Stingele, J., Habermann, B., and Jentsch, S. (2015). DNA-protein crosslink repair: proteases as DNA repair enzymes. Trends Biochem. Sci. *40*, 67–71.

Swenberg, J.A., Lu, K., Moeller, B.C., Gao, L., Upton, P.B., Nakamura, J., and Starr, T.B. (2011). Endogenous versus exogenous DNA adducts: their role in carcinogenesis, epidemiology, and risk assessment. Toxicol. Sci. *120 Suppl 1*, S130–145.

Zhitkovich, A., and Costa, M. (1992). A simple, sensitive assay to detect DNA-protein crosslinks in intact cells and in vivo. Carcinogenesis *13*, 1485–1489.

Molecular Cell, Volume *64*

Supplemental Information

Mechanism and Regulation

of DNA-Protein Crosslink Repair

by the DNA-Dependent Metalloprotease SPRTN

Julian Stingele, Roberto Bellelli, Ferdinand Alte, Graeme Hewitt, Grzegorz Sarek, Sarah L. Maslen, Susan E. Tsutakawa, Annabel Borg, Svend Kjær, John A. Tainer, J. Mark Skehel, Michael Groll, and Simon J. Boulton

Figure S1 (related to Figure 1)

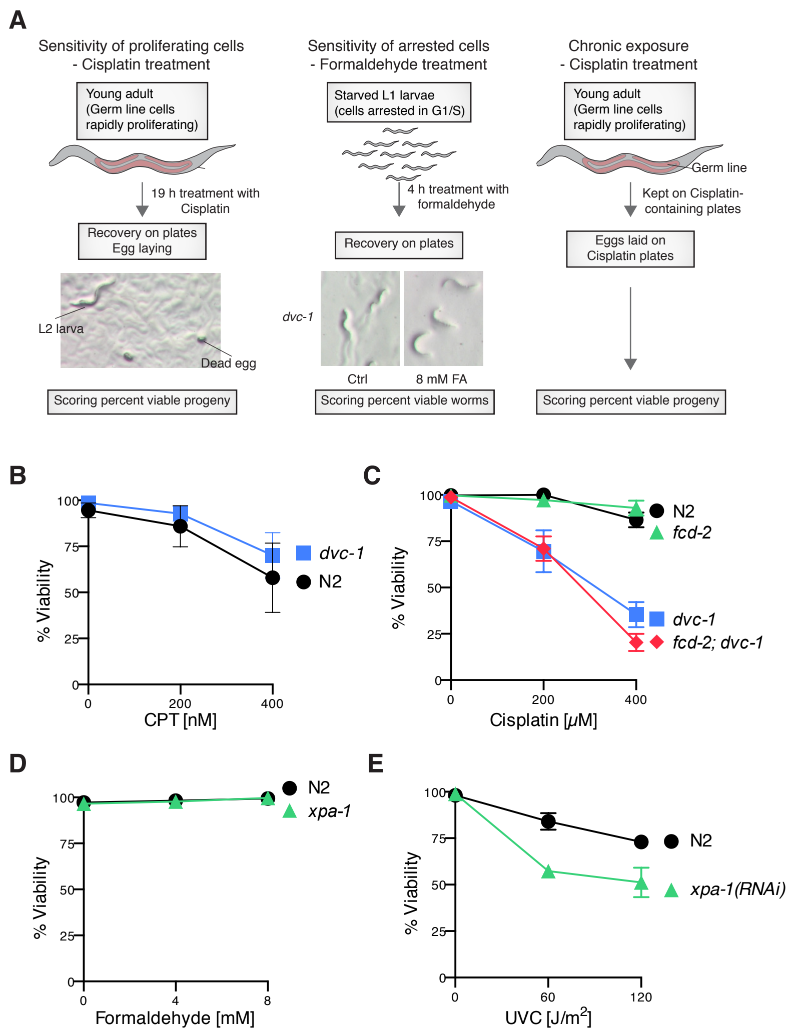

Figure S1 (related to Figure 1).

(A) Schematic representation of treatment regimens used to determine sensitivity towards DNA damage inducing agents in *C. elegans*.

(B) *C. elegans* mutant strains lacking functional SPRTN (*dvc-1*) are not sensitive towards camptothecin. Sensitivity was assessed by measuring embryonic survival of progeny after exposure of adult animals to the indicated doses. Error bars indicate SEM of two independent experiments.

(C) Progeny of FANCD2-deficient worms (*fcd-2*) do not show increased viability defects after exposure to cisplatin even in the absence of SPRTN (dvc-1) using the standard treatment regimen. Cisplatin sensitivity was assessed by measuring embryonic survival of progeny after exposure of young adult animals to the indicated doses. Error bars indicate SEM of 2 independent experiments.

(D) L1 larvae with a mutated *xpa-1* allele are not sensitive towards formaldehyde. Error bars indicated SEM of 2 independent experiments.

(E) Depletion of XPA (Xpa-1) by RNAi results in UV sensitivity in *C. elegans*. Error bars indicate SEM of 2 independent experiments.

Figure S2 (related to Figure 3)

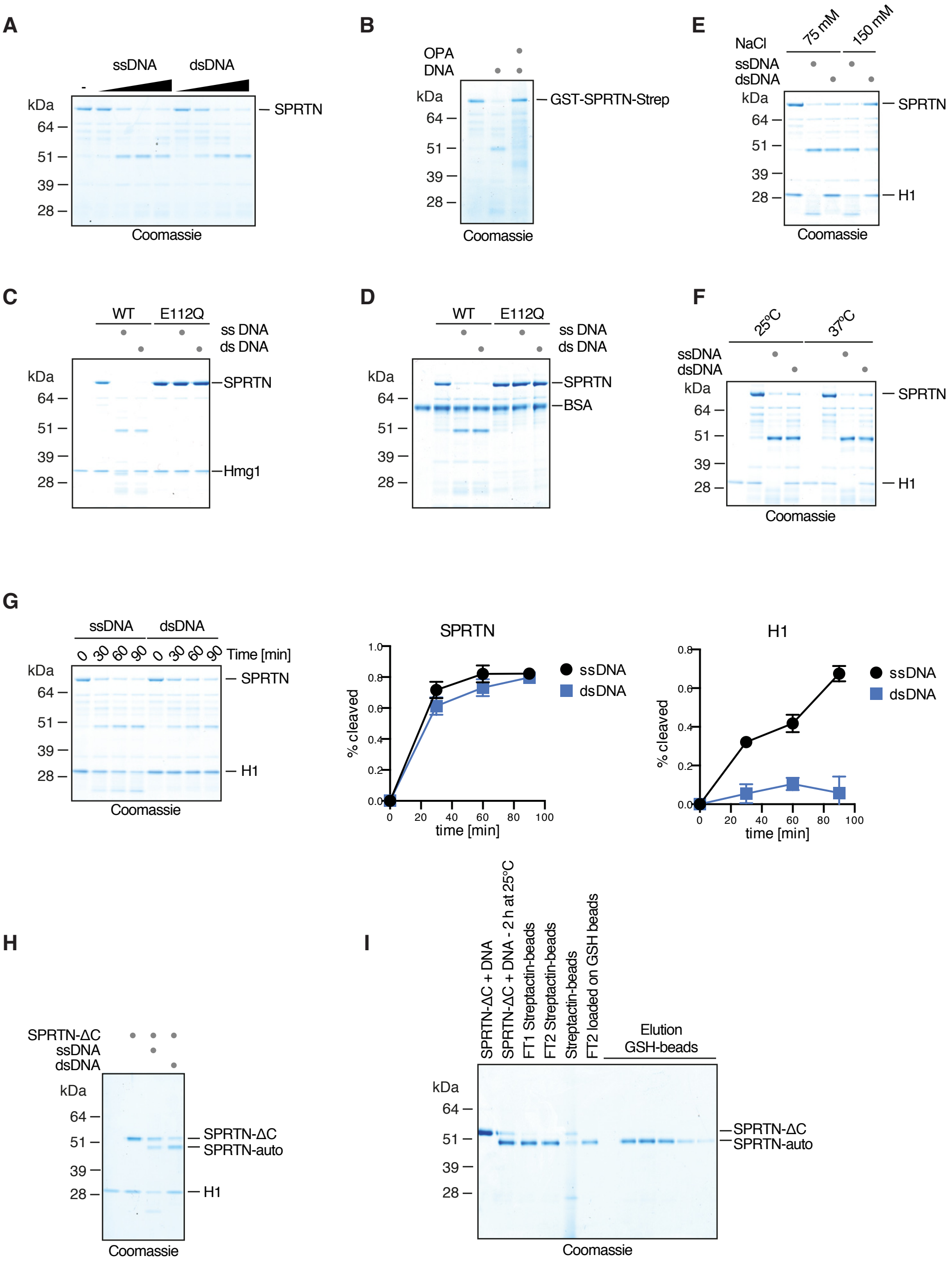

Figure S2 (related to Figure 3).

(A) Autocatalytic cleavage of SPRTN is induced similarly by single- and double-stranded DNA. Recombinant GST-SPRTN-Strep (180 nM) was incubated with different concentrations (0, 0.01, 0.1, 1 and 10 nM) of DNA (single- or double-stranded circular phage DNA). Reactions were stopped by addition of SDS-containing loading dye after a 2 h incubation at 25°C.

(B) DNA-dependent autocatalytic cleavage of SPRTN is inhibited by the metalloprotease inhibitor 1,10-phenanthroline (OPA). Recombinant GST-SPRTN-Strep (180 nM) was incubated in the absence or presence of DNA (single-stranded circular phage DNA, 10 nM) and with or without OPA (3 mM). Reactions were stopped by addition of SDS-containing loading dye after a 2 h incubation at 25°C.

(C-D) SPRTN cleaves DNA binding proteins in a single-stranded DNA-dependent manner. Recombinant GST-SPRTN-Strep (WT or the catalytically inactive E112Q variant, 480 nM) was incubated with the indicated recombinant substrates (360 nM) in the absence or presence of single- and double stranded phage DNA (10 nM). Reactions were stopped by addition of SDS-containing loading dye after a 2 h incubation at 25°C.

(E-F) Specificity of single-stranded DNA dependent substrate cleavage is independent of salt concentration and temperature. Recombinant GST-SPRTN-Strep (480 nM) was incubated with the recombinant histone H1 (360 nM) in the absence or presence of single- and double stranded phage DNA (10 nM). Reactions contained 75 mM NaCl if not indicated otherwise. Reactions were stopped by addition of SDS-containing loading dye after a 2 h incubation at 25°C (E) or in the indicated temperature (F).

(G) Kinetic analysis of auto- and substrate cleavage. Recombinant GST-SPRTN-Strep (240 nM) was incubated with the recombinant histone H1 (360 nM) in the absence or presence of single- and double stranded phage DNA (10 nM). Reactions contained 75 mM NaCl if not indicated otherwise. Reactions were stopped by addition of SDS-containing loading dye after the indicated time at 25°C. Left panel shows a representative gel; right panel shows quantification of two independent experiments.

(H) Residual substrate cleavage by SPRTN-ΔC is specifically induced by single-stranded DNA. Recombinant GST-SPRTN-Strep ΔC (480 nM) was incubated with recombinant Histone H1 (360 nM) in the absence or presence of single- and double stranded phage DNA (10 nM). Reactions were stopped by addition of SDS-containing loading dye after a 2 h incubation at 25°C.

(I) Purification of SPRTN-auto. GST-SPRTN-Strep ΔC was subjected to autocleavage by addition of DNA, which results in removal of the C-terminal Strep-tag. The reaction was stopped after 2 hours by DNA digestion through addition of micrococcal nuclease. Undigested SPRTN-ΔC was removed by passing the reaction twice over Streptactin-beads. The second flow-through (FT2) was collected and passed over GSH-beads. Finally, SPRTN-auto was eluted using reduced glutathione.

Figure S3 (related to Figure 4)

A

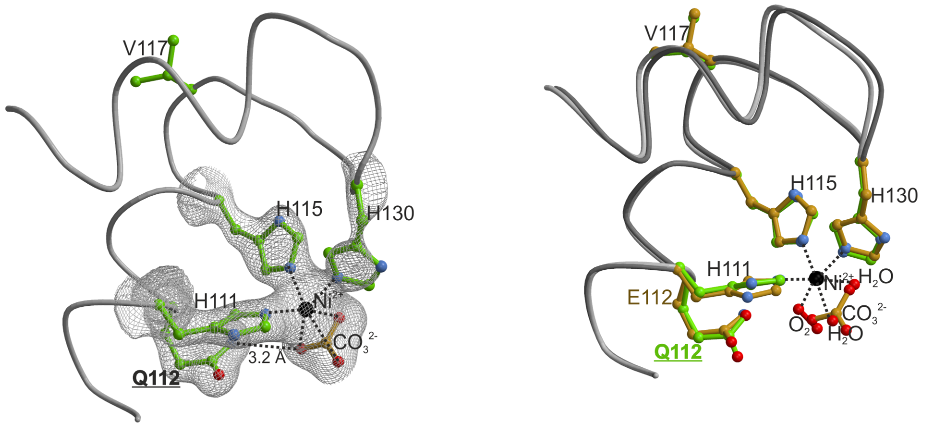

B

	100 110 120 130
H. sapiens	LRPRKDLVETLLHEMIHAYLFVTNNDKDREGHGPEFCKH
M. musculus	LRPRKDLVETLLHEMIHAYLFVTNNDKDREGHGPEFCKH
D. melanogaster	LRPRKDLVETLLHEMIHAYCFVLNIREGNGGHGPNFKRI
C. elegans	LRPRSDLVETLLHEMIHAYLFVKERNRDRDGHGPQFQAH
S. pombe b	FRDYKTVKSTLIHELTHNVHG.........EHDSSFWEL
S. pombe a	WLPFEFVMDVFLHELCHIWQG.........PHDRRFFSH
S. cerevisiae	FLPMECIMGTMLHELTHNLFG.........PHDKKFYNK
A. thaliana	FLSYHEILDTMLHELCHNAHG.........PHNASFYKL

Figure S3 (related to Figure 4).

(A) Close-up view of the active site of the *pombe* Wss1-E112Q mutant (PDB: 5LN5) illustrated in cartoon representation. The $2F_O$-F_C-electron density map (grey) of the active site is contoured to 1σ (left panel). Carbon atoms of His111, His115, His130 as well as Val117 are displayed in green. The general architecture of the active site is unaffected by the EQ mutation, as indicated by the overlay of WT and E112Q Wss1 (right panel). Notable, in the E112Q-mutant a carbonate completes the octahedral coordination of the Ni^{2+} atom.

(B) Alignment of active site sequences of members of the Wss1/SPRTN protease familiy. Catalytic residues are in red, Tyr117, which is mutated in Ruijs-Aalfs syndrome, is shown in blue and conserved residues are shaded in grey. Numbering corresponds to the human sequence.

Figure S4 (related to Figure 5)

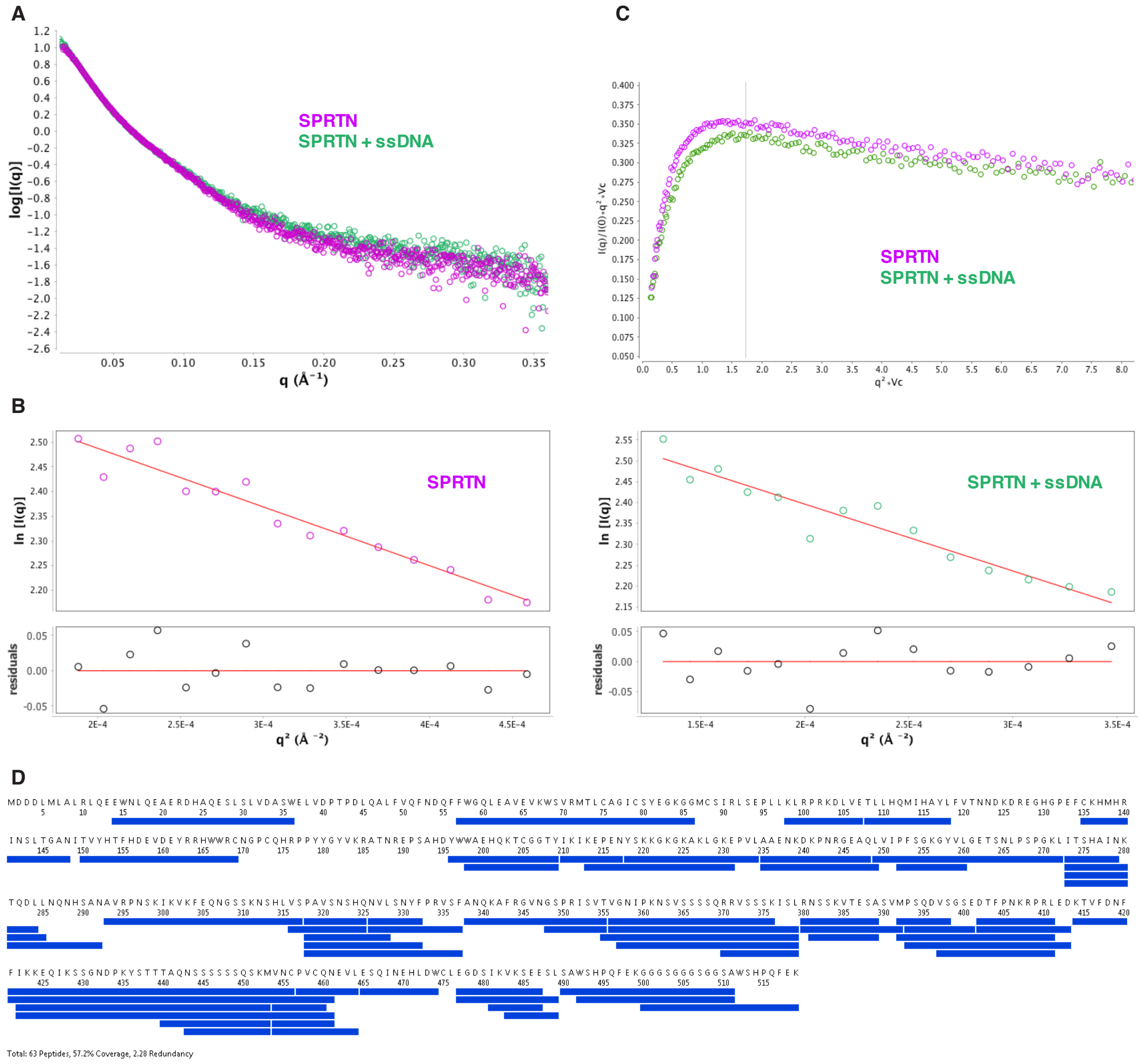

Figure S4 (related to Figure 5).

(A) SAXS curve. SAXS analysis indicates that binding of ssDNA increases the flexibility GST-SPRTN.

(B) Guinier analysis does not show aggregation.

(C) In the dimensionless Kratky, the decrease in peak height in the ssDNA-bound sample is consistent with increased flexibility.

(D) Sequence coverage of SPRTN obtained in hydrogen/deuterium exchange mass spectrometry experiments.

Figure S5 (related to Figure 6)

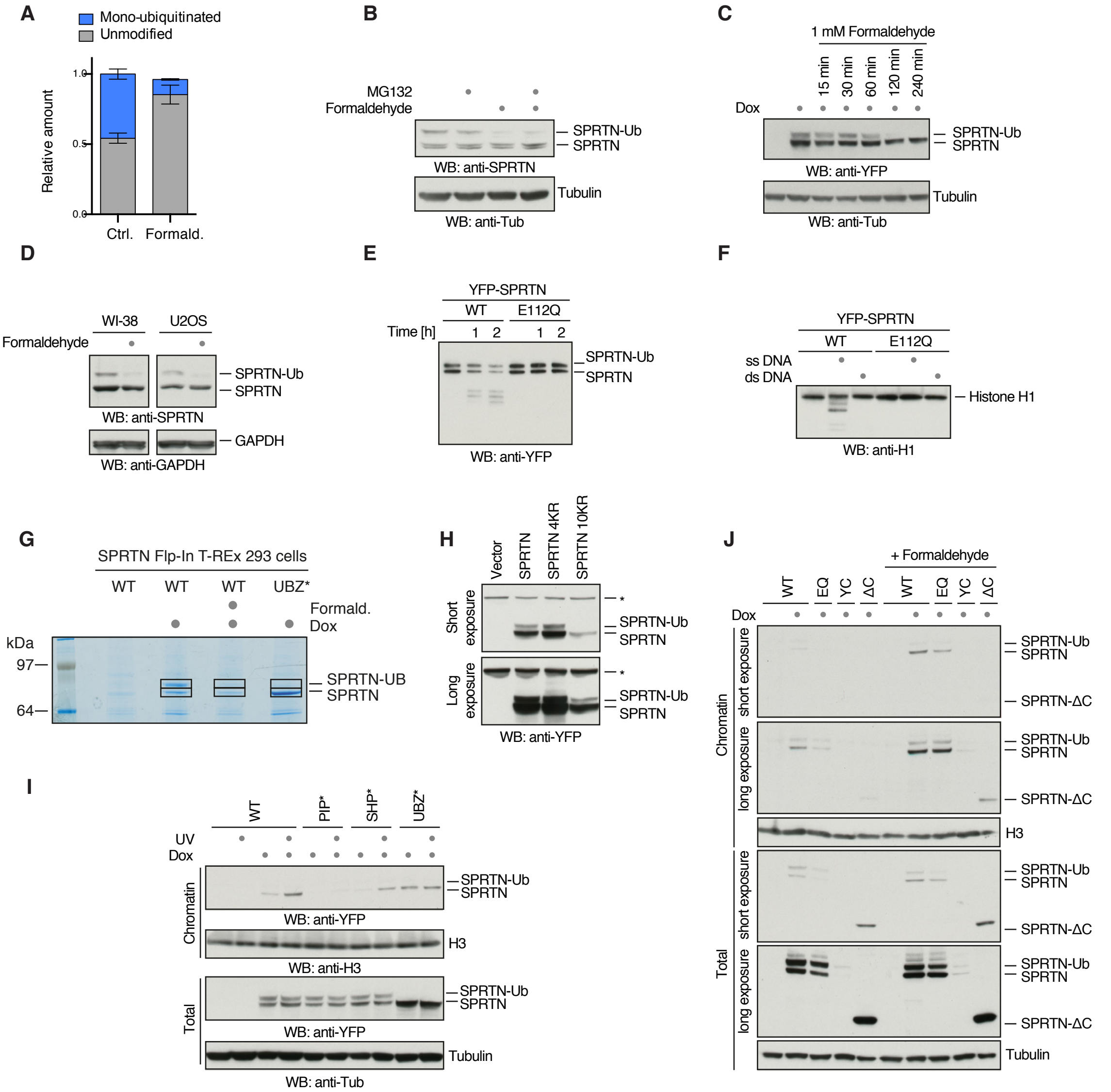

Figure S5 (related to Figure 6).

(A) Quantification of mono-ubiquitinated and unmodified SPRTN-WT before and after exposure to 1 mM formaldehyde. Western blots of three independent experiments were quantified with error bars representing SEM.

(B) SPRTN deubiquitination is independent of proteasomal degradation. U2OS cells were untreated or treated with 20 μM MG132 for 2 hours prior to addition of 1 mM formaldehyde (FA) for 2 hours. Cells were lysed directly in SDS-containing loading dye and subjected to SDS-PAGE followed by Western blotting using the indicated antibodies.

(C) SPRTN is deubiquitinated upon DPC induction by formaldehyde in a time-dependent manner. Doxycycline-inducible YFP-SPRTN-Strep HeLa Flp-In TRex cells were treated for the indicated amount of time with 1 mM formaldehyde prior to lysis in SDS-containing loading dye followed by SDS-PAGE and Western blotting using the indicated antibodies.

(D) Endogenous SPRTN is deubiquitinated upon DPC induction by formaldehyde. U2OS and WI-38 cells were treated with 1 mM formaldehyde (FA) for 2 hours. Cells were lysed directly in SDS-containing loading dye and subjected to SDS-PAGE followed by Western blotting using the indicated antibodies.

(E) Mono-ubiquitinated and unmodified SPRTN autocleave with similar kinetics. The indicated YFP-SPRTN-Strep variants were purified from 293 T-REx cells using Streptactin beads. Purified proteins were then incubated in the presence or absence of single-stranded phage DNA (10 nM) at 25ºC for the indicated amount of time. Reactions were stopped by addition of SDS-containing loading dye and analyzed by SDS-PAGE and Western blotting using the indicated antibodies.

(F) YFP-SPRTN-Strep cleaves histone H1 in a single-stranded DNA-dependent manner. Purified YFP-SPRTN-Strep, as in (E), (WT or the catalytically inactive E112Q variant) was incubated with recombinant histone H1 (100 nM) in the absence or presence of single- and double stranded phage DNA (10 nM). Reactions were stopped by addition of SDS-containing loading dye after a 4 h incubation at 25°C and analyzed by SDS-PAGE and Western blotting.

(G) Indicated YFP-SPRTN-Strep variants were purified from doxycycline-inducible YFP-SPRTN-Strep 293 Flp-In TRex using GFP-Trap agarose and subjected to mass spectrometry analysis. Deubiquitination was induced by a 2 hour formaldehyde exposure (1 mM).

(H) Analysis of SPRTN ubiquitination in KR variants. Plasmids coding for WT SPRTN or the indicated variants were transiently transfected in 293 Flp-In cells. Cells were lysed 24 hours after transfection and analyzed by Western blotting. Asterisk indicates an unspecific band serving as loading control.

(I) SPRTN's relocalization to recruitment upon UV-induced DNA damage depends on its binding to PCNA and ubiquitin. Doxycycline-inducible YFP-SPRTN-Strep HeLa Flp-In TRex cells expressing the indicated SPRTN variants were treated with UVC light (20 J/m^2) 2 hours before lysis in SDS-containing loading dye (total) or fractionation in soluble and chromatin components.

(J) SPRTN-ΔC is able to relocalize to chromatin upon DPC-induction by formaldehyde. Doxycycline-inducible YFP-SPRTN-Strep HeLa Flp-In TRex cells expressing the indicated SPRTN variants were treated with 1 mM formaldehyde (FA) for 2 hours prior to lysis in SDS-containing loading dye (total) or fractionation in soluble and chromatin components.

Figure S6 (related to Figure 7)

A

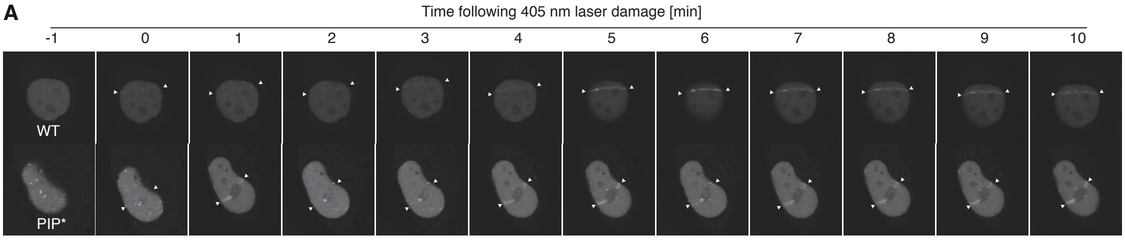

B

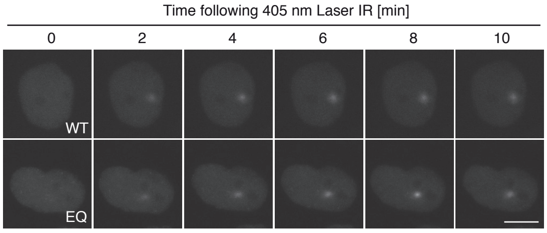

Figure S6 (related to Figure 7).

(A) Representative images of HeLa Flp-In TRex cells expressing WT or PIP* YFP-SPRTN-Strep after microirradiation (scale bar = 10 μm).

(B) Representative images of HeLa Flp-In TRex cells expressing WT or EQ YFP-SPRTN-Strep after microirradiation (scale bar = 10 μm).

EXTENDED EXPERIMENTAL PROCEDURES

C. elegans Strains and Methods

Strains were maintained using standard techniques on OP50 seeded MYOB plates supplemented with nystatin. N2 (Bristol) wild type, *fcd-2(tm1298)*, *him-6(ok412)* and *xpa-1(ok698)* mutant strains were as described previously. The *dvc-1(ok260)* mutant allele was obtained from Caenorhabditis Genetics Center, and outcrossed six times to N2 worms. All strains bearing *dvc-1(ok260)*, *fcd-2(tm1298) or him-6(ok412)* alleles were maintained as heterozygotes balanced by the *nT1(GFP)* balancer. Homozygotic mutants were selected by picking worms not expressing GFP in the pharynx.

C. elegans DNA damage sensitivity assays

Cisplatin. A cisplatin stock solution (2 mM, P4394, Sigma) was prepared in 150 mM NaCl. *Standard treatment*: Randomly picked young adult animals were treated with the indicated dose of cisplatin in 2 ml total volume M9 medium containing 300 µl of a stationary culture of OP50 bacteria at room temperature for 19 h on a slow shaker protected from light. Animals were washed three times in M9-T (M9 + 100 µl/l Triton X-100) and recovered for 1 h on OP50 seeded MYOB plates. Animals (typically 12-20 per dose and genotype) were then allowed to lay eggs on OP50 seeded MYOB plates for 4 hours. Dead eggs were counted 20 h after removing the parent animals; living animals were counted 24 h later (typically progeny of 12-15 animals was analyzed per dose and genotype). *Chronic treatment*: Randomly picked young adult animals were placed on MYOB plates containing 200 µM cisplatin or control plates (six per biological replicate). Worms were moved every 24 hours to new drug-containing plates. Embryonic survival of progeny was then determined by determining the number of hatched eggs on the 0-24, 24-48 and 48-72 hour plates.

Formaldehyde. Synchronized L1 larvae populations were obtained by bleaching gravid adult worms in M9 containing 0.5 N NaOH and 1 % sodium hypochlorite for 7 minutes. After three washes with M9, eggs were incubated in M9 for 17.5 h on a slowly rotating wheel. Synchronized L1 larvae were then splitted into several tubes and treated with the indicated dose of formaldehyde (28906, Thermofisher). After 4 hours of incubation L1 larvae were washed twice with M9 and transferred to OP50 seeded MYOB plates. Dead and living worms (typically 40-400 per dose and genotype) were scored after an incubation over night at 23 °C.

UV. Randomly picked young adult animals were exposed to the indicated dose of UVC light on OP50 seeded MYOB plates. After irradiation plates were incubated for 24 h 23 °C. Animals (typically 12-20 per dose and genotype) were then allowed to lay eggs on OP50 seeded MYOB plates for 4 hours. Dead eggs were counted 20 h after removing the parent animals; living animals were counted 24 h later (typically progeny of 12-15 animals was analyzed per dose and genotype).

Ionizing radiation. Randomly picked young adult animals were exposed to the indicated dose of ionizing radiation on OP50 seeded MYOB plates in a Cs-137 irradiator. After irradiation plates were incubated for 24 h 23 °C. Animals (typically 12-20 per dose and genotype) were then allowed to lay eggs on OP50 seeded MYOB plates for 4 hours. Dead eggs were counted 20 h after removing the parent animals; living animals were counted 24 h later (typically progeny of 12-15 animals was analyzed per dose and genotype).

Cell culture and generation of stable cell lines.

U2OS, WI-38, HeLa and MEF cells were grown in Dulbecco's modified Eagle Medium (DMEM) supplemented with 10% (v/v) fetal bovine serum (FBS), 100 Units/ml penicillin, 100 mg/mL streptomycin and 1% L-glutamine (GIBCO, Invitrogen). *Sprtn*$^{F/-}$ and *Sprtn*$^{F/+}$ MEFs were provided by Yuichi Machida (Mayo Clinic). *Fandc2*$^{-/-}$ and *Fandc2*$^{+/+}$ MEFs have been described previously (Adelman et al., 2013). HeLa and 293 cells expressing SPRTN WT and mutants were generated using the Flp-In-T-Rex system (Invitrogen) according to manufacturer's instructions and grown in DMEM supplemented with tetracycline free FBS (Clontech). Protein expression was induced by

overnight (16h) incubation with doxycycline (final concentration 1 mg/mL). Cells were transfected with Lipofectamine 2000 (Invitrogen) according to manufacturer's instructions.

Detection of formaldehyde-induced DNA-protein crosslinks

SPRTN knockout was induced in immortalized *Sprtn*$^{F/-}$ MEFs (clone H7) by treatment with 2 μM 4-hydroxy tamoxifen for 48 hours with untreated cells serving as control (Maskey et al., 2014). DPC were induced by treating cells with 200 μM formaldehyde for 1 hour. Cells were washed twice before recovery in fresh media. DPCs were measured using a KCl/SDS precipitation assay essentially as described before (Zhitkovich and Costa, 1992). To this end, cells were lysed at the respective time points by scraping in 400 μl denaturing lysis buffer (2 % SDS, 20 mM Tris/HCl pH 7.5), frozen in liquid nitrogen and stored at -80°C until further processing. After collecting all samples, lysates were thawed at 55°C for 5 min and sonicated (5 cycles, 30''/30''). Cellular protein was then precipitated by adding 400 μl 200 mM KCl, 20 mM Tris pH 7.5 and incubation on ice for 5 min. The precipitate was pelleted by centrifugation at 4°C at maximum speed using a microcentrifuge. Supernatant was saved and used for quantifying soluble DNA. The pellet was resuspended in 400 μl 200 mM KCl, 20 mM Tris pH 7.5 and resolved by shaking at 55°C for 5 min. The solution was cooled on ice for 5 min, and precipitate was again pelleted by centrifugation at maximum speed for 5 min. This wash procedure was repeated three times prior to final resuspension in 400 μl 200 mM KCl, 20 mM Tris pH 7.5. Proteins were digested by adding 0.2 mg/ml Proteinase K and incubation at 55°C for 45 min. After addition of 10 μl ultrapure BSA (50 mg/ml, Ambion), the solution was cooled on ice for 5 min followed again by centrifugation. The final supernatant, containing the initially crosslinked DNA, and the supernatant from the first wash were treated with 0.2 mg/ml RNase for 30 min at 37°C. DNA concentrations were determined using the Qubit dsDNA HS assay. The amount of DPCs was calculated as the ratio between DNA precipitated by SDS/KCl to total DNA (SDS/KCl precipitated plus soluble DNA). Relative values were obtained by subtracting basal DPC levels and normalization to values after FA addition.

Sensitivity assays mammalian cells

U2OS cells were transfected with SPRTN-specific (5'-UCAAGGAACCAGAGAAUUA-3') or control siRNA using Lipofectamine RNAimax. 48 hours after transfection cells were treated with different concentrations of DNA damaging agents in 6-well plates.

The media was exchanged 24 hours later and cells incubated for further 5 days. HeLa Flp-In TRex cells bearing the doxycycline-inducible YFP-SPRTN-Strep alleles were transfected with siRNA against endogenous SPRTN (5'- GUCAGGAAGUUCUGGUUAA-3') and incubated in the absence or presence of doxycycline for 48 hours. Cells were then treated for 48 hours with 100 μM formaldehyde and counted after an additional 4 day incubation. Cell numbers were then determined using a Countess automated cell counter.

Chromatin fractionation and immunofluorescence staining.

Chromatin fractionation experiments were performed as described before (Bellelli et al., 2014). In brief, cells in the mid-exponential phase of growth were collected by scraping in ice-cold 1X phosphate-buffered saline (PBS). Cell pellets were then directly resuspended in 1X Laemli buffer or incubated for 10 min in ice-cold CSK buffer (10 mM PIPES, pH 6.8, 100mM NaCl, 300 mM sucrose, 1mM MgCl2, 1 mM EGTA, 1mM DTT, 1 mM phenylmethylsufonyl fluoride, 10 µg/ml aprotinin) containing 0.5% Triton X-100. Chromatin-bound and soluble proteins were separated by low speed centrifugation (3,000 rpm, 3 min at 4°C). For each fraction, protein amounts deriving from comparable number of cells were analysed by SDS-PAGE and Western blotting.

For indirect immunofluorescence, cells were pre-extracted in CSK buffer containing 0.5% Triton X-100 (5 min on ice) and/or fixed in 4% paraformaldehyde, permeabilized with 0.5% Triton X-100 (5 min on ice), and then incubated with anti-GFP antibody (Abcam) for 1h at room temperature. Coverslips were washed and incubated with Alexa Fluor 488 goat anti-rabbit antibody (Invitrogen) for 30 min at room

temperature. After 5 min of DAPI counterstaining, coverslips were mounted in Glycerol/PBS (1:1) and pictures were acquired with a FV1000 Olympus confocal microscope.

Expression and Purification of Recombinant GST-SPRTN-STREP

A human SPRTN-Strep gene codon-optimized for expression in insect cells was purchased (ThermoFisher) and subcloned into pDEST20 plasmid. Viruses expressing GST-SPRTN-Strep variants were obtained using the Bac-to-Bac system. pDEST20 SPRTN plasmids were transformed into DH10Bac or DH10EMBacY *E coli* cells for transposition into the bacmid. After 48h, blue/white selection of colonies was used to identify the recombinants. The bacmids were extracted from a 2 ml overnight culture and checked by PCR. Postitive bacmids were transfected into *Spodoptera frugiperda (*Sf21) cells using Fugene HD as transfection reagent. 0.8 x 10^6 cells were plated in a 6-well plate and let to attach at 27°C for one hour. In the meantime, 500 ng of bacmid and 5 μl of Fugene HD were added to 1 ml of Sf900 III medium, free of antibiotics/supplements. The medium was removed and the DNA-lipid mix added. After 5 h at 27°C, the DNA/lipid mix was removed and 2 ml of Sf900III medium supplemented with Fungizone was added. After 3 days at 27°C, 1.5 ml of the P1 virus was added to 25 ml Sf21 culture at 10^6 cells/ml. From day 1 to day 3, the culture was monitored for signes of infection (swollen cells). The spun supernatant (P2 virus) was titered using qPCR. The P2 pellet was checked for expression of GST-SPRTN-STREP. If positive, the P2 virus was used to infect Sf21 at high density (over 5x10^6 cells/ml) at MOI 3 in Sf900III medium supplemented with Glucose, Lactalbumin and Yeastolate. After 3 days, the culture was harvested and stored at -80°C. Cell pellets were lysed on ice in 0.5 - 2 ml lysis buffer (50 mM HEPES pH 7.5, 1 M NaCl, 1 % IGEPAL CA-630, 1 mM $MgCl_2$, 10 % glycerol, 0.04 mg/ml Pefabloc SC, cOmplete EDTA-free protease inhibitor cocktail tablets (1 tablet/50 ml)) per 10^7 cells. After addition of 4 U Benzonase/ml and sonication (2 x 20 pulses) with a large flat tip using a Branson Sonifier 450 (duty cycle 80%, output control 8), lysates were incubated for 30 min on ice. Lysates, typically 45 ml, were cleared by centrifugation at 4°C and applied to batch purification columns containing 1 ml bed volume of Strep-Tactin Superflow (Qiagen) resin, which had been prewashed using 4 x 1 ml lysis buffer. The flowthrough was discarded and the beads were washed with 4 CVs lysis buffer and 2 CVs wash buffer (50 mM HEPES pH 7.5, 250 mM NaCl, 10 % glycerol). Finally, proteins were eluted using 6 x 0.5 CVs of elution buffer (50 mM HEPES pH 7.5, 250 mM NaCl, 10 % glycerol, 10 mM d-Desthiobiotin). Fraction 2-5 were pooled and dialyzed twice against 50 mM HEPES pH 7.5, 250 mM NaCl, 10 % glycerol, before freezing in liquid nitrogen and storage at -80°C. GST-SPRTN 200-250 was expressed in *E. coli* using according to standard expression and purification protocols.

Purification of GST-SPRTN-auto

GST-SPRTN-auto was generated by incubating 1 mg of recombinant GST-SPRTN-ΔC-Strep with 16 μg single stranded DNA (ΦX174 virion, NEB) at 25 °C for 2 h to induce autocleavage. DNA was then digested by addition of 16.5 U/ml Benzonase (Millipore) and MgCl2 (final concentration 1 mM). After an incubation for 20 minutes on ice NaCl was added to a final concentration of 1 M. The solution was then passed twice through 0.5 ml bead volume Strep-Tactin Superflow resin to remove uncleaved GST-SPRTN-ΔC-Strep. The second flow through was incubated with 0.25 ml Glutathione Sepharose 4 Fast Flow (GE) for 1 h at 4 °C and applied to a disposable batch purification column. The resin was washed with 50 mM HEPES pH 7.5, 250 mM NaCl, 10 % glycerol before elution of GST-SPRTN-auto with 6 times 0.125 ml elution buffer (50 mM HEPES pH 7.5, 250 mM NaCl, 10 % glycerol, 10 mM reduced glutathione). Fractions containing GST-SPRTN-auto were pooled and dialyzed twice against 50 mM HEPES pH 7.5, 250 mM NaCl, 10 % glycerol.

Expression and Purification of Recombinant Wss1 (*S. cerevisiae*)

Wss1 was purified as described previously (Stingele et al., 2014).

DNA binding assays

Electrophoretic mobility shift assays (EMSAs) were used to analyze DNA binding of recombinant proteins. *Oligos:* Proteins were prepared at different concentrations in 250 mM NaCl, 50 mM HEPES pH 7.5, 10 % glycerol and mixed with an equal volume of DNA solution (0.5 μM fluorescently-labeled DNA (Alexa488-5'- ACGCTGCCGAATTCTACCAGTGCCTTGCTA-3'), 0.2 mg/ml BSA, 15 μM HEPES pH 7.5). Following an incubation for 20 min on ice, protein-DNA complexes were resolved on 6 % retardation gels (ThermoFisher) at 80 V for 80 min at 4°C and visualized on a Typhoon imager. Contrast of scanned images was adjusted using Adobe Photoshop software. *Phage DNA:* Proteins were prepared at different concentrations in 250 mM NaCl, 50 mM HEPES pH 7.5, 10 % glycerol and mixed with an equal volume of DNA solution (50 nM single- or double stranded ΦX174 DNA in TE). Following an incubation for 20 min on ice, protein-DNA complexes were resolved on 0.8 % agarose gels (containing EtBr) at 125 V for 300 min at 4°C.

Expression and Purification of Recombinant Wss1 (*S. pombe*)

Schizosaccharomyces pombe carries two homologous *Wss1*-genes, which we termed *SpWss1a* and *SpWss1b*. SpWss1a (UniProt-ID: Q9P7B5) corresponds to Wss1 of *Saccharomyces cerevisiae*; SpWss1b (UniProt-ID: O94580) possesses an additional N-terminal ubiquitin-like domain. Both SpWss1a and SpWss1b contain a WLM-domain (Wss1 like metalloprotease). Beside the full-length genes (applied primers: Wss1_Sp_for CTAGGATCCGAGTTGAAATTTAGTTGCAGAGG; Wss1_Sp_rev CTACTGCAGTTACTCCTTTTGGACTTTACTACC), truncated constructs were amplified by PCR-techniques based on secondary structure predictions calculated with *Jalview* to remove terminal, putative flexible regions that might be deleterious for crystallization tendency. Resulting gene versions were cloned into the plasmid pRSETA_His_Tev using *Bam*HI and *Pst*I restriction sites, expressed and tested for soluble expression in the *E. coli* strain BL21 (DE3). For three liter large scale cultivations, the expression strain was grown to an OD_{600} of 0.5 to 0.7. At this stage, the temperature of the culture was adjusted to 20 °C in a cold water bath and IPTG was added to a final concentration of 0.5 mM. After overnight expression at room temperature, cells were harvested by centrifugation. Protein purifications were carried out with ÄKTA™ chromatography platforms. For this purpose, the supernatant originating from the preceding cell disruption by using a French Press System was loaded on a HisTrap™ FF Ni-NTA column. After that, the column was washed with 100 mM Tris/HCl (pH 8.0), 500 mM NaCl, 20 mM imidazole, the protein was eluted by applying a linear gradient with a final concentration of 100 % 100 mM Tris/HCl (pH 8.0), 500 mM NaCl, 500 mM imidazole. In order to increase the crystallization tendency of the target protein, the affinity tag was removed by enzymatic digestion. To this end, the protein was transferred into 10 mM Tris/HCl (pH 8.0) and TEV-protease was added to the target protein in a mass ratio of 1:100 until cleavage was completed. An additional Ni-NTA chromatography was used to separate the cleaved protein from the tag and the His-tagged TEV protease. Size exclusion chromatography (Superdex75) with 50 mM Tris/HCl pH 8.0 and 200 mM NaCl yielded pure Wss1 protein of at least 10 mg, which was stored at -20 °C for further use.

Crystallization and Structure Determination of SpWss1b (17 - 151)

Initial crystallization trials were carried out for all constructs, however, only the SpWss1b short WLM-domain (17 – 151, sequence numbering according to human SPRTN) resulted in ordered crystals. The protein concentration varied from 10-17 mg/ml and crystal droplets consisted of 1 volume of reservoir solution (100 mM Tris/HCl (pH 7.5), 20% PEG 3350) and 1 volume of protein suspension. SpWss1b (17 – 151) crystallized after few days with a typical size of about 150 × 70 × 30 μm^3, whereas crystals of the E112Q mutant (108 - 282) grew within three months. Crystals were cryoprotected by a 1:1 mixture of mother liquor and 20% (v/v) glycerol and subsequently supercooled in a stream of nitrogen gas at 100 K. The structure of Wss1 was determined by single-wavelength anomalous dispersion (SAD) at a resolution of 1.0 Å. To this end, an anomalous data set had been collected at the SLS synchrotron (Villigen, Switzerland). A fluorescence energy scan prior to anomalous data set collection at 1.8 Å resolution (Table 1) identified the central metal ion not as Zn, but as Ni. It seems very likely that the Ni-ion occurs as an artifact of the Ni-IMAC purification procedure. Data processing for SpWss1b (17 – 151) yielded the orthorhombic space group $P2_12_12_1$

with the unit cell axes of a = 40.3 Å, b = 41.3 Å, c = 68.5 Å. SHELXD located 1 strong heavy atom site and subsequent SHARP-SAD phasing as well as SOLOMON solvent flattening resulted in appropriate phases of about 1.8 Å (Bricogne et al., 2003; Sheldrick, 2010). The calculated electron density revealed well defined secondary structure elements including defined side chains, thus allowing automated protein chain tracing and model building with ARP/wARP (Langer et al., 2008). Positional refinement with REFMAC (Murshudov et al., 1997; Vagin et al., 2004) further improved phases, so that missing structural parts could be completed. Subsequently, the model was refined against the native dataset collected at 1.0 Å resolution (Table 1). Finally, Translation/Libration/Screw vibrational motion refinement yielded current crystallographic values of R_{crys} = 14.3 %, R_{free} = 16.9 %, r.m.s. bond length = 0.009 Å, and r.m.s. angles = 1.4°. The geometry of the whole molecule, including the two N-terminal amino acids (Gly and Ser), originating from the *Bam*HI restriction site, is well defined in the electron density map, except for a loop of 9 amino acids (Pro66 – Thr76; numbers according to full-length SpWss1b), which is structurally distorted. Table 1 gives an overview of the refinement statistics. Notably, His111, His115, His130, as well as 2 water molecules and 1 oxygen molecule coordinate the Ni^{2+} ion by forming a distorted octahedron. The E112Q-mutant of SpWss1b (17 – 151) crystallized in the space group $P2_1$ within 3 month. The structure was determined by molecular replacement using the coordinates of SpWss1b (17 – 151) for Patterson Search calculations at a resolution of 1.75 Å. The asymmetric unit contains 2 molecules. Final refinement yielded crystallographic values of R_{crys} = 17.3 %, R_{free} = 19.5 %, r.m.s. bond length = 0.005 Å, and r.m.s. angles = 1.0° (Table 1). Interestingly, the Ni^{2+} atom in the mutant is complexed to a CO_3^{2-}, which completes the octahedral coordination of the ion.

Analysis of conformational changes

***Limited proteolysis*.** Conformational changes of SPRTN upon DNA-binding were determined using a limited proteolysis assay. Reactions were performed in 20 *μ*l containing 6 *μ*l catalytic inactive GST-SPRTN-EQ-Strep (1.2 *μ*M in 50 mM HEPES pH 7.5, 250 mM NaCl, 10 % glycerol), 2 *μ*l DNA (100 nM single-stranded circular DNA (ΦX174 virion, NEB) or double-stranded circular DNA (ΦX174 RF I, NEB) in TE), 10 *μ*l H_2O and 2 *μ*l Trypsin Gold (Promega, 5 ng/*μ*l in 50 mM HEPS pH 7.5). Reactions were stopped at the indicated time points by addition of 4 x LDS sample buffer (ThermoFisher) supplemented with β-mercaptoethanol and boiling at 95°C for 10 min. Samples were resolved on 12% Bis-Tris gradient gels, stained with InstantBlue and scanned on a Licor Odyssey imager. Bands were quantified using ImageJ. Contrast of scanned images was adjusted using Adobe Photoshop software to highlight low abundant cleavage fragments. Alternatively, gels were subjected to western blotting with GST-or Strep-specific antibodies.

SAXS analysis. SAXS data was collected on catalytically inactive GST-SPRTN-EQ-Strep in the presence and absence of DNA. SPRTN protein was exchanged into 50 mM HEPES pH 7.5, 250 mM KCl, and 1% glycerol with a final concentration 0.7 mg/ml. For the DNA complex, SPRTN (370 ul 0.63 mg/ml protein) was mixed 1:1.2 molar ratio with 15mer ssDNA (5'-ACGCTGCCGAATTCT-3'); diluted with 2 mL 50 mM HEPES pH 7.5, 75 mM KCl, and 1% glycerol; and concentrated to a similar concentration as DNA-free SPRTN, based on final volume and I(0). Data was collected on a Pilatus 2M detector at the SIBYLS beamline 12.3.1 at the Advanced Light Source (Classen et al., 2013; Dyer et al., 2014). The sample to detector distance was 1.6 m. Data was collected at 11 keV at 10° C. Scattering data were analyzed with the program SCÅTTER (https://bl1231.als.lbl.gov/scatter/), except for the electron pair distribution plot which was calculated with the Gnom program (Svergun, 1992). For protein and protein/DNA respectively, the real space Rg was 65 and 73 Å, I(0) was 16 and 15 (arbitrary detector units), and the Dmax was 265 and 293 Å. The Guinier Rg was 59 and 69 Å and I(0) was 15 and 15 (arbitrary detector units), respectively. The molecular mass calculated from the experimental data was respectively 140 and 150 kD for protein and protein/DNA, within 20% error with the GST fusion (theoretical molecular weight 85 kD) dimerizing and in the case of the complex, a dimer with two 4.5 kD ssDNA. The Porod exponent was 2.7 for DNA-free protein and 2.5 for DNA-bound protein.

Hydrogen/deuterium exchange mass spectrometry. Deuterium exchange reactions of SPRTN were initiated by diluting the protein in D_2O (99.8% D_2O ACROS, Sigma, UK) in 50 mM Tris, 250mM NaCl,1mM TCEP pH 7.5 buffer to give a final D_2O percentage of 90%. For all experiments, deuterium labelling was carried out at 23°C (unless otherwise stated) at three time points (3 sec on ice (0.3 sec), 3 sec, and 30 sec in triplicate. The labelling reaction was quenched by the addition of chilled 2.4% v/v formic acid in 2 M guanidinium hydrochloride and immediately frozen in liquid nitrogen. Samples were stored at -80°C prior to analysis. The quenched protein samples were rapidly thawed and subjected to proteolytic cleavage by pepsin followed by reversed phase HPLC separation. Briefly, the protein was passed through an Enzymate BEH immobilized pepsin column, 2.1 x 30 mm, 5 μm (Waters, UK) at 200 μL/min for 2 min and the peptic peptides trapped and desalted on a 2.1 x 5 mm C18 trap column (Acquity BEH C18 Van-guard pre-column, 1.7 μm, Waters, UK). Trapped peptides were subsequently eluted over 12 min using a 5-36% gradient of acetonitrile in 0.1% v/v formic acid at 40 μL/min. Peptides were separated on a reverse phase column (Acquity UPLC BEH C18 column 1.7 μm, 100 mm x 1 mm (Waters, UK). Peptides were detected on a SYNAPT G2-Si HDMS mass spectrometer (Waters, UK) acquiring over a m/z of 300 to 2000, with the standard electrospray ionization (ESI) source and lock mass calibration using [Glu1]-fibrino peptide B (50 fmol/μL). The mass spectrometer was operated at a source temperature of 80°C and a spray voltage of 2.6 kV. Spectra were collected in positive ion mode. Peptide identification was performed by MS^e (Silva et al., 2005) using an identical gradient of increasing acetonitrile in 0.1% v/v formic acid over 12 min. The resulting MS^e data were analyzed using Protein Lynx Global Server software (Waters, UK) with an MS tolerance of 5 ppm. Mass analysis of the peptide centroids was performed using DynamX sotware (Waters, UK). Only peptides with a score >6.4 were considered. The first round of analysis and identification was performed automatically by the DynamX software, however, all peptides (deuterated and non-deuterated) were manually verified at every time point for the correct charge state, presence of overlapping peptides, and correct retention time. Deuterium incorporation was not corrected for back-exchange and represents relative, rather than absolute changes in deuterium levels. Changes in H/D amide exchange in any peptide may be due to a single amide or a number of amides within that peptide. All time points in this study were prepared at the same time and individual time points were acquired on the mass spectrometer on the same day.

Laser microirradiation induced DNA damage

In order to detect local recruitment of SPRTN to laser induced DPCs YFP-SPRTN-Strep-expressing HeLa Flp-In TRex cells were seeded on 35 mm glass bottom dish (Ibidi, 81158). Cells were pre-sensitized for 48h with 10 μM BrdU and expression of YFP-tagged protein was induced with 24h of 1 μg/ml doxycycline. Immediately prior to imaging DMEM was exchanged for C02 independent media (ThermoFisher Scientific #18045-045). Cells were transferred to Olympus FV1000 confocal LSM with heated stage. Laser microirradition was performed with a 405 nm laser focused through 40x objective (400mW at objective, 50 scans). Time-course was acquired on the same system, imaging every 30s for 10 min.

FRAP

DNA damage was generated using laser microirradiation on an Olympus FV1000 confocal LSM as described above. FRAP experiments were performed on damage sites after initial recruitment had plateaued ≥15 min following induction. FRAP and image acquisition was performed on the same system. Bleaching was performed following 15 frames of pre-bleach acquisition with a 0.1 s pulse from 405 nm Laser, images were acquired for a further 600 frames (1 frame = 0.01 s). Analysis was performed on ≥15 cells using FRAP profiler plugin for imageJ (http://rsb.info.nih.gov/ij/). 1/2t and %mobile were calculated using GraphPad prism.

Antibodies ab76949

Polyclonal anti-SPRTN antibody was a gift from John Rouse, anti-GST (ab92), anti-H3 (ab10799), anti-GAPDH (ab8245), anti-H1.10 (ab11079), anti-H2B (ab1790) and anti-Strep (ab76949) antibodies were purchased from Abcam, anti-Tub (T6074) and anti-GFP (11814460001, used for detection of

YFP) (11079) from Sigma, anti-Chk2 (phospho-T68, 2661S) from NEB, anti-Chk1 (phospho-S345, 2348L) from Cell Signaling, anti-H2A (07-146) from Millipore.

SUPPLEMENTARY REFERENCES

Adelman, C.A., Lolo, R.L., Birkbak, N.J., Murina, O., Matsuzaki, K., Horejsi, Z., Parmar, K., Borel, V., Skehel, J.M., Stamp, G., *et al.* (2013). HELQ promotes RAD51 paralogue-dependent repair to avert germ cell loss and tumorigenesis. Nature *502*, 381-384.

Bellelli, R., Castellone, M.D., Guida, T., Limongello, R., Dathan, N.A., Merolla, F., Cirafici, A.M., Affuso, A., Masai, H., Costanzo, V., *et al.* (2014). NCOA4 transcriptional coactivator inhibits activation of DNA replication origins. Molecular cell *55*, 123-137.

Bricogne, G., Vonrhein, C., Flensburg, C., Schiltz, M., and Paciorek, W. (2003). Generation, representation and flow of phase information in structure determination: recent developments in and around SHARP 2.0. Acta Crystallographica Section D: Biological Crystallography *59*, 2023-2030.

Classen, S., Hura, G.L., Holton, J.M., Rambo, R.P., Rodic, I., McGuire, P.J., Dyer, K., Hammel, M., Meigs, G., Frankel, K.A., *et al.* (2013). Implementation and performance of SIBYLS: a dual endstation small-angle X-ray scattering and macromolecular crystallography beamline at the Advanced Light Source. J Appl Crystallogr *46*, 1-13.

Dyer, K.N., Hammel, M., Rambo, R.P., Tsutakawa, S.E., Rodic, I., Classen, S., Tainer, J.A., and Hura, G.L. (2014). High-throughput SAXS for the characterization of biomolecules in solution: a practical approach. Methods in molecular biology *1091*, 245-258.

Langer, G., Cohen, S.X., Lamzin, V.S., and Perrakis, A. (2008). Automated macromolecular model building for X-ray crystallography using ARP/wARP version 7. Nat Protoc *3*, 1171-1179.

Maskey, R.S., Kim, M.S., Baker, D.J., Childs, B., Malureanu, L.A., Jeganathan, K.B., Machida, Y., van Deursen, J.M., and Machida, Y.J. (2014). Spartan deficiency causes genomic instability and progeroid phenotypes. Nature communications *5*, 5744.

Murshudov, G.N., Vagin, A.A., and Dodson, E.J. (1997). Refinement of macromolecular structures by the maximum-likelihood method. Acta Crystallogr D Biol Crystallogr *53*, 240-255.

Sheldrick, G.M. (2010). Experimental phasing with SHELXC/D/E: combining chain tracing with density modification. Acta Crystallogr D Biol Crystallogr *66*, 479-485.

Silva, J.C., Denny, R., Dorschel, C.A., Gorenstein, M., Kass, I.J., Li, G.Z., McKenna, T., Nold, M.J., Richardson, K., Young, P., *et al.* (2005). Quantitative proteomic analysis by accurate mass retention time pairs. Anal Chem *77*, 2187-2200.

Stingele, J., Schwarz, M.S., Bloemeke, N., Wolf, P.G., and Jentsch, S. (2014). A DNA-Dependent Protease Involved in DNA-Protein Crosslink Repair. Cell *158*, 327-338.

Svergun, D. (1992). Determination of the regularization parameter in indirect-transform methods using perceptual criteria. Journal of applied crystallography *25*, 495-503.

Vagin, A.A., Steiner, R.A., Lebedev, A.A., Potterton, L., McNicholas, S., Long, F., and Murshudov, G.N. (2004). REFMAC5 dictionary: organization of prior chemical knowledge and guidelines for its use. Acta Crystallogr D Biol Crystallogr *60*, 2184-2195.
Zhitkovich, A., and Costa, M. (1992). A simple, sensitive assay to detect DNA-protein crosslinks in intact cells and in vivo. Carcinogenesis *13*, 1485-1489.
